# Synthesis of sterically shielded piperidine nitroxides via acid-catalyzed heterocyclization of β-aminoketone derivatives with ketones

**DOI:** 10.3762/bjoc.22.74

**Published:** 2026-06-17

**Authors:** Mark M Gulman, Yurii I Glazachev, Sergey A Dobrynin

**Affiliations:** 1 N. N. Vorozhtsov Novosibirsk Institute of Organic Chemistry, Siberian Branch of the Russian Academy of Sciences, Novosibirsk, 630090, Russia; 2 Novosibirsk State University, Pirogova Str. 2, Novosibirsk, 630090, Russia; 3 Voevodsky Institute of Chemical Kinetics and Combustion, Siberian Branch of the Russian Academy of Sciences, Novosibirsk, 630090, Russia

**Keywords:** heterocyclization, piperidine nitroxide, TEEPONE synthesis

## Abstract

The capabilities of modern methods for the synthesis of sterically shielded piperidine nitroxides with acyclic substituents are largely limited to symmetrical tetraethyl structures and do not allow the introduction of functional groups into position 2. We propose an alternative approach that allows for the variation of substituents adjacent to the nitroxyl group, which significantly expands the potential of sterically hindered nitroxides for promising applications in materials science and structural biology. The new heterocyclization strategy implies the construction of a 2,2,6-trisubstituted piperidine scaffold from β-aminoketone acetals and dialkyl ketones under acid catalysis. The resulting amines were oxidized to the corresponding ketonitrones and subsequent reaction with moderately basic organometallic reagents, such as 2-alkynyl- and 2-allylmagnesium halides, enables the facile introduction of diverse substituents, including those with functional groups. If necessary, the multiple carbon–carbon bonds in the side chain can be subjected to hydrogenation to give saturated alkyl or functionalized alkyl groups. The study of reduction kinetics for alkyl and allyl-substituted piperidine nitroxides in ascorbate/glutathione media (30% EtOH, pH 7.5) yielded second-order rate constants of ≈10^−2^ M^−1^·s^−1^, which is close to that earlier reported for 2,2,6,6-tetraethylpiperidine (TEEPONE).

## Introduction

Since their discovery in 1959 by Lebedev and Kazarnovsky, stable nitroxides of the piperidine series (2,2,6,6-tetramethylpiperidine-1-oxyl (TEMPO) derivatives) hold a prominent position as compounds of significant practical importance. Their applications range widely, from fundamental research to industrial technologies. In the spin-labeling technique, these radicals have been extensively used in biophysics and structural biology [1]. Piperidine-based radicals were the first used for measuring distances in proteins by the PELDOR method at room temperature [2]. Piperidine nitroxyl biradicals form the basis of the most efficient commercial agents for dynamic nuclear polarization (DNP) in NMR studies of biomolecules and solid samples [3]. They are also employed as contrast agents for magnetic resonance imaging (MRI) [4–5] and as spin probes for Overhauser MRI in vivo [6]. Piperidin-1-oxyls played a key role in the development and industrial implementation of controlled polymerization of vinyl monomers (nitroxide-mediated polymerization, NMP) [7]. The unique redox properties of these radicals underpin their use as catalysts for alcohol oxidation [8], electrode active materials in energy storage devices (batteries) [9], as well as antioxidants and superoxide dismutase (SOD) mimetics [10].

Their sterically shielded analogs, such as 2,2,6,6-tetraethylpiperidine-1-oxyl (TEEPO), were initially developed for nitroxide-mediated polymerization (NMP), because shielding with ethyl or bulkier alkyl groups decreases accessibility of the nitroxide moiety and improves the equilibrium parameters in the reversible trapping of alkyl radical of the growing polymer chain [11]. This shielding also alters the redox properties of nitroxides and increases their lifetimes in biological systems [12], which makes these radicals promising MRI contrast agents [13], spin probes for in vivo EPRI [14–15] and antioxidant tissue protectors [16].

Existing methods for the synthesis of such sterically hindered piperidine nitroxide radicals are based on approaches employing either the tetraethyl analog of phorone or acetonine [11,15,17–18] or the desulfurization of dispirothiapyranone derivatives [19–22]. Consequently, the structural diversity of radicals bearing acyclic substituents is practically limited to derivatives of 2,2,6,6-tetraethylpiperidine-1-oxyl. Given the significant interest in this class of compounds, there is a clear need for new synthetic strategies enabling the introduction of diverse acyclic substituents at positions 2 and/or 6 of the piperidine ring.

In our previous works we have shown how the reactions of cyclic nitrones with organometallic compounds is a fruitful approach for the synthesis of sterically shielded radicals of the pyrrolidine, imidazoline, and imidazolidine series [23], including those with functional groups in the side chains [24]. Here we applied this strategy for the synthesis of sterically hindered piperidine nitroxyl radicals. Six-membered cyclic nitrones – 2,2,6-trialkyl-substituted 2,3,4,5-tetrahydropyridine-1-oxides were prepared via the acid-catalyzed reaction of 2-(2-aminoalkyl)-2-methyl-1,3-dioxolanes with ketones in analogy to literature procedures [25–28], followed by oxidation in the tungstate/hydrogen peroxide system [29]. It should be noted that no case of heterocyclization has ever been described where the resulting piperidine cycle contained three substituents at the 2- and 6-positions of the heterocycle. Subsequent treatment of these nitrones with alkynyl- or allylmagnesium halides gave 2-alkynyl or 2-allyl-substituted nitroxides. Finally, carbon–carbon multiple bonds were removed via Pd-catalyzed hydrogenation and the nitroxide group was recovered by mild oxidation. This approach was used to prepare both symmetric 2,2,6,6-tetraethyl- and 2,2,6,6-tetrapropyl-substituted piperidine nitroxides, and sterically shielded nitroxides with functional groups in the side chain of the heterocycle.

## Results and Discussion

**Scheme 1:**
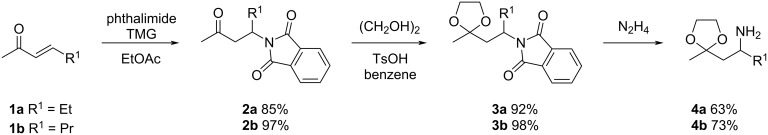
Synthesis of aminodioxolanes **4a**,**b**. TMG = 1,1,3,3-tetramethylguanidine.

Aminodioxolanes **4a**,**b** were obtained from enones **1a**,**b** via a modified literature sequence [30] involving phthalimide Michael addition, dioxolane formation, and hydrazinolysis (Scheme 1). The target 2-(2-aminoalkyl)-1,3-dioxolanes **4a**,**b** were obtained in pure form after vacuum distillation.

Conversion of α,β-unsaturated ketones **1a**,**b** to aminodioxolanes **4a**,**b** proceeded in 49% and 69% overall yield, respectively. In the ^1^H NMR spectrum of **4a**, characteristic signals of the dioxolane fragment protons (multiplet, 3.72–3.79 ppm, 4H) and an isolated methyl group (singlet, 1.13 ppm, 3H) were observed. The NH_2_ protons appeared as a broad signal at 1.77–1.89 ppm. In addition, an eight-spin system comprising the ethyl group resonances (0.71, 1.13, 1.19 ppm), the N-adjacent methine (2.71 ppm), and diastereotopic methylene protons (1.39, 1.58 ppm) supported the assigned structure. Compound **4b** exhibited spectral data identical to those previously described [31].

**Scheme 2:**
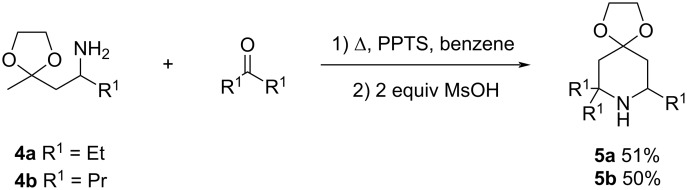
Acid-catalyzed heterocyclization of β-aminoketones **4a**,**b** with diethyl and dipropyl ketones.

The aminodioxolanes **4a**,**b** were subjected to acid-catalyzed heterocyclization with the corresponding diethyl and dipropyl ketones following a reported procedure [29]. In a typical experiment, a mixture of aminodioxolane and ketone was heated in benzene in the presence of pyridinium *p*-toluenesulfonate (PPTS) using a Dean–Stark apparatus to facilitate Schiff base formation. After cessation of water evolution, methanesulfonic acid (2.0 equiv) was added dropwise to the refluxing mixture, and heating was continued for an additional hour. The reaction was quenched with aqueous sodium carbonate, and the resulting piperidines **5a**,**b** were purified by vacuum distillation. The target 2,2,6-triethyl- and 2,2,6-tripropyl-substituted piperidines **5a**,**b** were isolated in approximately 50% yield (Scheme 2).

The full-line shape analysis of the ^1^H NMR spectrum of piperidine **5a** was simulated using the gNMR software (version 5.0) [32] and the extracted spin-system parameters were consistent with the proposed structure (see Supporting Information File 1). For compound **5b**, such analysis was not feasible due to extensive overlap of methylene proton resonances associated with the propyl substituents; nevertheless, the spectral characteristics were analogous to those observed for **5a**. Notably, HRMS analysis of **5a**,**b** exhibited fragment ions corresponding to α-cleavage adjacent to the nitrogen atom, resulting in loss of the 2- or 6-alkyl substituent ([M−R]^+^) in agreement with established fragmentation patterns for cyclic amines [33].

**Scheme 3:**
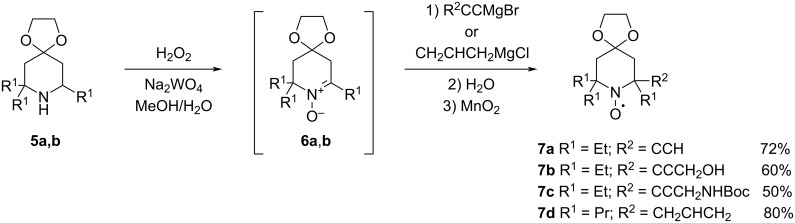
Synthesis of piperidine nitroxides from piperidines **5a**,**b** and organometallic reagents.

The 2,2,6-trisubstituted piperidines **5a**,**b** were oxidized to the corresponding ketonitrones **6a**,**b** using a sodium tungstate/hydrogen peroxide system. Attempted purification led to decomposition; therefore, crude **6a** and **6b** were used directly in the next step. It is known that the reaction of sterically hindered nitrones may lead to metalation or deoxygenation of the alkylnitrone group instead of nucleophilic addition [34–35]. Conversely, less basic organometallic reagents, such as vinylmagnesium bromide [34], ethynylmagnesium bromide [23], or allylmagnesium chloride [36], readily undergo addition, yielding the corresponding nitroxides. Ketonitrone **6a** was treated with various alkynylmagnesiun bromides, generated by deprotonation of acetylene, propargyl alcohol, or *N*-Boc-protected propargylamine with ethylmagnesium bromide, whereas nitrone **6b** was treated with allylmagnesium bromide. Subsequent quenching of the reaction mixtures with water, followed by oxidation with MnO_2_, afforded the corresponding nitroxides **7a**–**d** in 50–80% overall yield from piperidines **5a**,**b** (Scheme 3).

The structures of nitroxides **7a**–**d** were confirmed by ^1^H NMR spectroscopy of their amine derivatives, obtained via Zn/CF_3_COOH reduction in CD_3_OD, following a reported procedure [37]. The full line-shape analysis of the ^1^H NMR spectra for reduced nitroxides **7a**–**c** (see Supporting Information File 1) confirmed the 7,7,9-triethyl-1,4-dioxa-8-azaspiro[4.5]decane core and revealed distinct alkynyl signatures: a singlet at 3.29 ppm (1H) for **7a**, a singlet at 4.28 ppm (2H) for **7b**, and two singlets at 3.89 ppm (2H) and 1.46 ppm (9H) for **7c**. For the allyl derivative **7d**, extensive signal overlap prevented rigorous simulation, but characteristic terminal vinyl protons at 5.83, 5.28, 5.27 ppm were clearly observed. Elemental analysis and HRMS data were in agreement with the proposed structures.

**Scheme 4:**
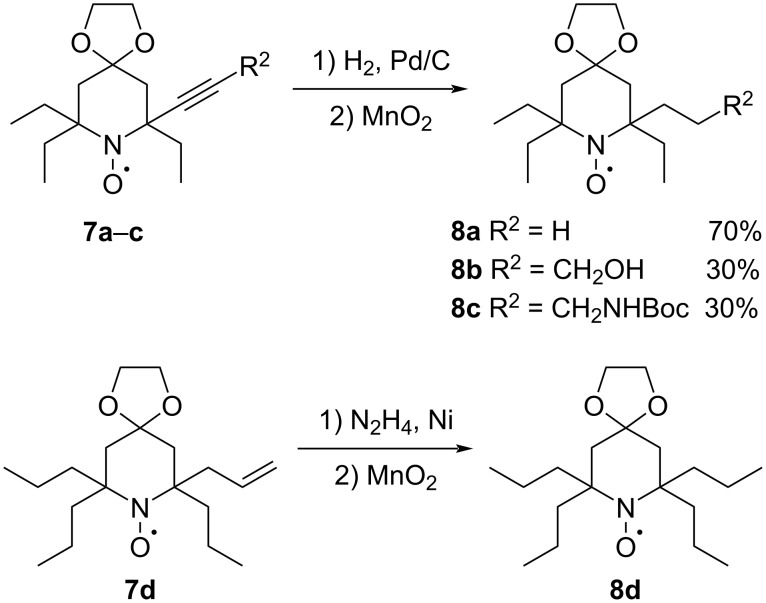
Hydrogenation of carbon–carbon multiple bonds of nitroxides **7a**–**d**.

Subsequent hydrogenation of the unsaturated carbon–carbon bonds afforded the corresponding saturated derivatives **8a**–**d**. Specifically, alkynyl derivatives **7a**–**c** were hydrogenated with H_2_ over a palladium catalyst, while the allyl derivative **7d** was reduced with hydrazine over Raney nickel (Scheme 4). The absence of signals corresponding to alkene protons in the ^1^H NMR spectra of **8a–d** confirms the lack of partially hydrogenated impurities. Elemental analysis and HRMS data were consistent with the proposed molecular formulas.

Notably, among the alkynyl derivatives, high hydrogenation yields 70% were observed exclusively for the terminal ethynyl derivative **7a**. The lower hydrogenation yields for **7b** and **7c** presumably stem from labile hydrogens in the substituents that promote N–O bond cleavage under hydrogenation conditions, leading to the corresponding amines [37]. It should be noted, however, that this proposed pathway remains speculative and requires further investigation.

**Scheme 5:**
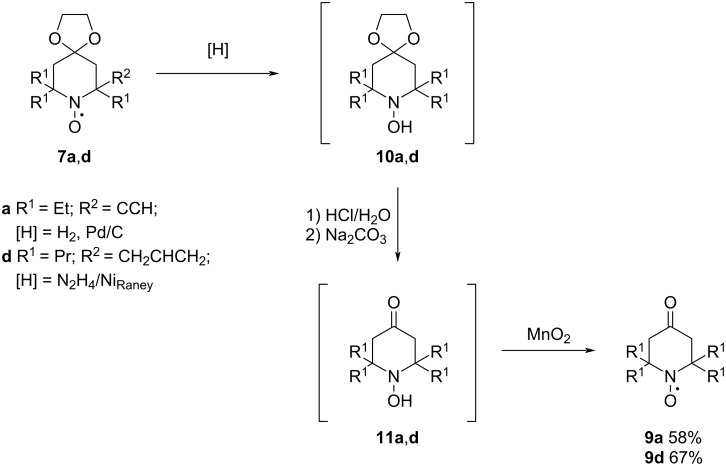
Sequential hydrogenation and dioxolane deprotection.

In a separate set of experiments, aqueous hydrochloric acid was added to the reaction mixtures after the hydrogenation of **7a**,**d** was complete. Under these conditions the dioxolane protecting group was removed and nitroxides **9a**,**d** were isolated in 58% and 67% yield, respectively (Scheme 5).

The IR spectrum of the nitroxide **9a** (TEEPONE) was identical to that reported in the literature [21], and the IR spectrum of **9d** was similar, with a strong absorption band at 1716 cm^−1^, characteristic of the carbonyl (C=O) stretching vibrations. The ^1^H NMR spectra of the reduced derivatives of **9a**,**d** showed no signals of the dioxolane moiety protons, confirming complete deprotection. The signal intensities for the methylene protons at the 3- and 5-positions of the heterocycle were remarkably diminished due to H/D exchange under the acidic conditions employed for sample preparation.

All EPR spectra were consistent with a single nitroxide species with a g-factor in the range of 2.0055–2.0057 (a_N_ = 15–16 G) (Table 1); no additional hyperfine splitting was resolved. The slightly higher a_N_ values in the alkynyl-substituted compounds likely stem from the electron-withdrawing influence of the alkyne group and/or conformational changes of the piperidine ring. Reduction rate constants for **7d**, **8a**,**d**, and **9a**,**d** were measured in 30% EtOH (pH 7.5) using the ascorbate/glutathione system, yielding values of ≈10^−2^ M^−1^·s^−1^, which are comparable to that of TEEPONE. Notably, the reduction rate constants determined for TEEPONE in 30% aqueous ethanol are virtually indistinguishable from those reported for pure aqueous solution [12,21]. Notably, compound **7a** was reduced by glutathione alone in the absence of ascorbate, precluding accurate second-order rate determination under standard conditions. This enhanced reactivity likely stems from the electron-withdrawing character of the proximal ethynyl group, which renders the nitroxide moiety a stronger oxidant and enables its reduction by glutathione (Table 1).

**Table 1:** EPR spectra parameters and reduction rate constants.

Nitroxide	a_N_, G ± 0.05 G	Hp-p^a^, G ± 0.05 G	*k*_2_, 10^2^ M^−1^·s^−1^ in water/ethanol solution

**7a**	15.48	1.78	nd^b^
**7b**	16.00	1.75	^c^
**7c**	16.00	1.85	^c^
**7d**	15.48	1.88	7.8 ± 0.3
**8a**	15.63	2.00	4.5 ± 0.5
**8b**	15.73	1.95	^c^
**8c**	15.73	1.98	^c^
**8d**	15.52	1.80	5.9 ± 0.1
**9a**	14.88	1.93	6.2 ± 0.5
**9d**	15.19	1.66	7.6 ± 0.4

^a^Peak-to-peak linewidth of the central EPR line. ^b^Accurate quantification of the reduction rate constant proved unattainable owing to the fast reduction process. ^c^Values were not determined.

## Conclusion

Here we provided yet another demonstration of the synthetic advantages of a previously proposed general approach to the synthesis of sterically shielded nitroxides based on the reaction of sterically hindered nitrones with moderately basic organometallic reagents followed by hydrogenation of carbon–carbon multiple bonds.

In this work we applied this method to the synthesis of sterically shielded nitroxides of the piperidine series. Along with TEEPONE and its dioxolane-protected derivative, a number of new nitroxides were prepared, including those with four larger alkyl groups (propyl ones) adjacent to the nitroxide moiety and those with a functional group in the side chain. These functionalized nitroxides can hardly be prepared by any of the previously used methods. Taking into account the fact that piperidine nitroxides are traditionally used as precursors of nitroxyl radicals of other types, especially the pyrroline and pyrrolidine series, this method may open up new possibilities in the chemistry of nitroxide spin labels and nitroxide-based functional materials.

## Supporting Information

File 1 Experimental section and characterization data of synthesized compounds.

## Data Availability

All data that supports the findings of this study is available in the published article and/or the supporting information of this article.
