# Supplementary material for: Synthesis of sterically shielded piperidine nitroxides via acid-catalyzed heterocyclization of β-aminoketone derivatives with ketones
Source: Beilstein J Org Chem. 2026 Jun 17;22:948–54. doi: 10.3762/bjoc.22.74 (PMC13284749; doi:10.3762/bjoc.22.74)
Supplement: File 1 — Experimental section and characterization data of synthesized compounds. [file Beilstein_J_Org_Chem-22-948-s001.pdf]

BEILSTEIN JOURNAL OF ORGANIC CHEMISTRY

Supporting Information

for

Synthesis of sterically shielded piperidine nitroxides via acid-catalyzed heterocyclization of β-aminoketone derivatives with ketones

Mark M. Gulman, Yurii I. Glazachev and Sergey A. Dobrynin

Beilstein J. Org. Chem. **2026,** *22,* 948–954. doi:10.3762/bjoc.22.74

Experimental section and characterization data of synthesized compounds

License and Terms: This is a supporting information file under the terms of the Creative Commons Attribution License (https://creativecommons.org/licenses/by/4.0). Please note that the reuse, redistribution and reproduction in particular requires that the author(s) and source are credited and that individual graphics may be subject to special legal provisions.
The license is subject to the *Beilstein Journal of Organic Chemistry* terms and conditions: (https://www.beilstein-journals.org/bjoc/terms)

Table of contents

Experimental S5

Materials and methods S5

Synthesis........ S6

General method of Michael reaction S6

3-(1,3-Dioxoisoindolin-2-yl)-hexan-5-one (**2a**) S7

4-(1,3-Dioxoisoindolin-2-yl)-heptan-6-one (**2b**)........ S7

Dioxolanation S7

1-(2-Methyl-1,3-dioxolan-2-yl)-2-(1,3-dioxoisoindolin-2-yl)butane (**3a**) S7

1-(2-Methyl-1,3-dioxolan-2-yl)-2-(1,3-dioxoisoindolin-2-yl)pentane (**3b**) S8

General method of hydrazinolysis S8

1-(2-Methyl-1,3-dioxolan-2-yl)butan-2-amine (**4a**)........ S8

1-(2-Methyl-1,3-dioxolan-2-yl)pentan-2-amine (**4b**)........ S9

General method of heterocyclization S9

7,7,9-Triethyl-1,4-dioxa-8-azaspiro[4.5]decane (**5a**)........ S9

7,7,9-Tripropyl-1,4-dioxa-8-azaspiro[4.5]decane (**5b**) S10

General method for 2-alkynylpiperidine-1-oxyl synthesis........ S10

7,7,9-Triethyl-9-ethynyl-1,4-dioxa-8-azaspiro[4.5]decane-8-oxyl (**7a**)........ S11

7,7,9-Triethyl-9-(3-hydroxyprop-1-yn-1-yl)-1,4-dioxa-8-azaspiro[4.5]decan-8-oxyl (**7b**)........ S11

7-(3-((*tert*-Butoxycarbonyl)amino)prop-1-yn-1-yl)-7,9,9-triethyl-1,4-dioxa-8-azaspiro[4.5]decan-8-oxyl (**7c**)........ S12

7,7,9-Tripropyl-9-(prop-2-en-1-yl)-1,4-dioxa-8-azaspiro[4.5]decane-8-oxyl (**7d**)........ S12

7,7,9,9-Tetraethyl -1,4-dioxa-8-azaspiro[4.5]decane-8-oxyl (**8a**) S13

7,7,9,9-Tetraethyl-1,4-dioxa-8-azaspiro[4.5]decane- 8-oxyl (**8a**) S13

7,7,9-triethyl-9-(3-hydroxypropyl)-1,4-dioxa-8-azaspiro[4.5]decan-8-oxyl (**8b**) S14

7-(3-((*tert*-Butoxycarbonyl)amino)propyl)-7,9,9-triethyl-1,4-dioxa-8-azaspiro[4.5]decan-8-oxyl (**8c**) S14

7,7,9,9-Tetrapropyl-1,4-dioxa-8-azaspiro[4.5]decane-8-oxyl (**8d**)........ S15

2,2,6,6-Tetraethylpiperidin-4-one-1-oxyl (**9a**) S15

2,2,6,6-Tetrapropylpiperidin-4-one-1-oxyl (9d)........ S16

NMR spectra S17

^{1}H NMR(400 MHz, $CDCl_3$) of 3-(1,3-dioxoisoindolin-2-yl)-hexanone-5 (**2a**)........ S17

^{13}C {^{1}H} NMR(100 MHz, $CDCl_3$) of 3-(1,3-dioxoisoindolin-2-yl)-hexanone-5 (**2a**). S17

^{1}H NMR (500 MHz, $CDCl_3$) of 1-(2-methyl-1,3-dioxolan-2-yl)-2-(1,3-dioxoisoindolin-2-yl)butane (**3a**)........ S18

^{13}C {^{1}H} NMR (125 MHz, $CDCl_3$) of 1-(2-methyl-1,3-dioxolan-2-yl)-2-(1,3-dioxoisoindolin-2-yl)butane (**3a**) S18

^{1}H NMR (500 MHz, $CDCl_3$) of 1-(2-methyl-1,3-dioxolan-2-yl)butan-2-amine (**4a**) S19

^{13}C {^{1}H} NMR (125MHz, $CDCl_3$) of 1-(2-methyl-1,3-dioxolan-2-yl)butan-2-amine (**4a**) S19

^{1}H NMR (500MHz, $CDCl_3$) of 4-(1,3-dioxoisoindolin-2-yl)-heptanone-6 (**2b**) S20

^{13}C {^{1}H} NMR (125MHz, $CDCl_3$) of 4-(1,3-dioxoisoindolin-2-yl)-heptanone-6 (**2b**) S20

^{1}H NMR (600 MHz, $CDCl_3$) of 1-(2-methyl-1,3-dioxolan-2-yl)-2-(1,3-dioxoisoindolin-2-yl)pentane (**3b**) S21

^{13}C {^{1}H} NMR (150 MHz, $CDCl_3$) of 1-(2-methyl-1,3-dioxolan-2-yl)-2-(1,3-dioxoisoindolin-2-yl)pentane (**3b**) S21

^{1}H NMR (500 MHz, $CDCl_3$) of 1-(2-methyl-1,3-dioxolan-2-yl)pentan-2-amine (**4b**) S22

^{13}C {^{1}H} NMR (500 MHz, $CDCl_3$) of 1-(2-methyl-1,3-dioxolan-2-yl)pentan-2-amine (**4b**) S22

^{1}H NMR (500 MHz, $CDCl_3$) of 7,7,9-triethyl-1,4-dioxa-8-azaspiro[4.5]decane (**5a**) S23

^{13}C {^{1}H} NMR (125 MHz, $CDCl_3$) of 7,7,9-triethyl-1,4-dioxa-8-azaspiro[4.5]decane (**5a**) S23

^{1}H NMR (300 MHz, $CDCl_3$) of 7,7,9-tripropyl-1,4-dioxa-8-azaspiro[4.5]decane (**5b**) S24

^{13}C {^{1}H} NMR (75 MHz, $CDCl_3$) of 7,7,9-tripropyl-1,4-dioxa-8-azaspiro[4.5]decane (**5b**) S24

^{1}H NMR (300MHz, CD_3OD, Zn/CF_3COOH system) of 7,7,9-triethyl-9-ethynyl-1,4-dioxa-8-azaspiro[4.5]decane-8-oxyl (**7a**) S25

^{1}H NMR (300 MHz, CD_3OD, Zn/CF_3COOH) of 7,7,9-triethyl-9-(3-hydroxyprop-1-yn-1-yl)-1,4-dioxa-8-azaspiro[4.5]decan-8-oxyl (**7b**) S25

^{1}H NMR (300 MHz, CD_3OD, Zn/CF_3COOH) of 7-(3-((*ter*t-butoxycarbonyl)amino)prop-1-yn-1-yl)-7,9,9-triethyl-1,4-dioxa-8-azaspiro[4.5]decan-8-oxyl (**7c**) S26

^{1}H NMR (400 MHz, CD_3OD, Zn/CF_3COOH system) of 7,7,9,9-tetraethyl-1,4-dioxa-8-azaspiro[4.5]decane-8-oxyl (**8a**) S26

^{1}H NMR (400 MHz, CD_3OD, Zn/CF_3COOH system) of 2,2,6,6-tetraethylpiperidin-4-one-1-oxyl (**9a**) S27

^{1}H NMR (400 MHz, CD_3OD, Zn/CF_3COOH) of 7,7,9-triethyl-9-(3-hydroxypropyl)-1,4-dioxa-8-azaspiro[4.5]decan-8-oxyl (**8b**) S27

^{1}H NMR (300 MHz, CD_3OD, Zn/CF_3COOH) of 7-(3-((*tert*-butoxy-carbonyl)amino)propyl)-7,9,9-triethyl-1,4-dioxa-8-azaspiro[4.5]decan-8-oxyl (**8c**) S28

^{1}H NMR (300 MHz, CD_3OD, Zn/CF_3COOH system) of 7,7,9-tripropyl-9-(prop-2-en-1-yl)-1,4-dioxa-8-azaspiro[4.5]decane-8-oxyl (**7d**) S28

^{1}H NMR (300 MHz, CD_3OD, Zn/CF_3COOH system) of 7,7,9,9-tetrapropyl-1,4-dioxa-8-azaspiro[4.5]decane-8-oxyl (**8d**) S29

^{1}H NMR (300 MHz, CD_3OD, Zn/CF_3COOH system) of 2,2,6,6-tetrapropylpiperidin-4-one-1-oxyl (**9b**) S29

Line shape analysis of NMR multiplets S30

Line shape analysis of multiplets for 7,7,9-triethyl-1,4-dioxa-8-azaspiro[4.5]decane (**5a**) S30

Table S1. Spin system parameters for 7,7,9-triethyl-1,4-dioxa-8-azaspiro[4.5]decane (**5a**) S31

Line shape analysis of multiplets for 7,7,9-triethyl-9-ethynyl-1,4-dioxa-8-azaspiro[4.5]decane-8-oxyl (**7a**) S32

Table S2. Spin system parameters for 7,7,9-triethyl-9-ethynyl-1,4-dioxa-8-azaspiro[4.5]decane-8-oxyl (**7a**) S33

Line shape analysis of multiplets for 7,7,9-triethyl-9-(3-hydroxyprop-1-yn-1-yl)-1,4-dioxa-8-azaspiro[4.5]decan-8-oxyl (**7b**) S34

Table S3. Spin system parameters for 7,7,9-triethyl-9-(3-hydroxyprop-1-yn-1-yl)-1,4-dioxa-8-azaspiro[4.5]decan-8-oxyl (**7b**) S35

Line shape analysis of multiplets for 7-(3-((*tert*-butoxycarbonyl)amino)prop-1-yn-1-yl)-7,9,9-triethyl-1,4-dioxa-8-azaspiro[4.5]decan-8-oxyl (**7c**) S36

Table S4. Spin system parameters for 7-(3-((*tert*-butoxycarbonyl)amino)prop-1-yn-1-yl)-7,9,9-triethyl-1,4-dioxa-8-azaspiro[4.5]decan-8-oxyl (**7c**) S37

IR spectra S38

IR (neat) of 3-(1,3-dioxoisoindolin-2-yl)-hexanone-5 (**2a**) S38

IR (neat) of 1-(2-methyl-1,3-dioxolan-2-yl)-2-(1,3-dioxoisoindolin-2-yl)butane (**3a**) S38

IR (neat) of 1-(2-methyl-1,3-dioxolan-2-yl)butan-2-amine (**4a**) S38

IR (neat) of 4-(1,3-dioxoisoindolin-2-yl)-heptanone-6 (**2b**). S39

IR (neat) of 1-(2-methyl-1,3-dioxolan-2-yl)-2-(1,3-dioxoisoindolin-2-yl)pentane (**3b**) S39

IR (neat) of 1-(2-methyl-1,3-dioxolan-2-yl)pentan-2-amine (**4b**) S39

IR (neat) of 7,7,9-triethyl-1,4-dioxa-8-azaspiro[4.5]decane (**5a**) S40

IR (KBr) of 7,7,9-triethyl-9-ethynyl-1,4-dioxa-8-azaspiro[4.5]decane-8-oxyl (**7a**) S40

IR (neat) of 7,7,9-triethyl-9-(3-hydroxyprop-1-yn-1-yl)-1,4-dioxa-8-azaspiro[4.5]decan-8-oxyl (**7b**) ..S40

IR (neat) of 7-(3-((*tert*-butoxycarbonyl)amino)prop-1-yn-1-yl)-7,9,9-triethyl-1,4-dioxa-8-azaspiro[4.5]decan-8-oxyl (**7c**) S41

IR (KBr) of 7,7,9,9-tetraethyl -1,4-dioxa-8-azaspiro[4.5]decane-8-oxyl (**8a**) S41

IR (neat) of 7,7,9-triethyl-9-(3-hydroxypropyl)-1,4-dioxa-8-azaspiro[4.5]decan-8-oxyl (**8b**) S41

IR (neat) of 7-(3-((*tert*-butoxycarbonyl)amino)propyl)-7,9,9-triethyl-1,4-dioxa-8-aza-spiro[4.5]decan-8-oxyl (**8c**) S42

IR (KBr) of 2,2,6,6-tetraethylpiperidin-4-one-1-oxyl (**9a**) S42

IR (neat) of 7,7,9-tripropyl-1,4-dioxa-8-azaspiro[4.5]decane (**5b**) S42

IR (neat) of 7,7,9-triprorpyl-9-(prop-2-en-1-yl)-1,4-dioxa-8-azaspiro[4.5]decane-8-oxyl (**7d**) S43

IR (neat) of 7,7,9,9-tetrapropyl-1,4-dioxa-8-azaspiro[4.5]decane-8-oxyl (**8d**) S43

IR (KBr) of 2,2,6,6-tetrapropylpiperidin-4-one-1-oxyl (**9d**) S43

Gas chromatography–mass spectrometry S44

GC–MS of 3-(1,3-dioxoisoindolin-2-yl)-hexanone-5 (**2a**) S44

GC–MS of 1-(2-methyl-1,3-dioxolan-2-yl)-2-(1,3-dioxoisoindolin-2-yl)butane (**3a**) S45

GC–MS of 1-(2-methyl-1,3-dioxolan-2-yl)butan-2-amine (**4a**)....S46

GC–MS of 4-(1,3-dioxoisoindolin-2-yl)-heptanone-6 (**2b**). S47

GC–MS of 1-(2-methyl-1,3-dioxolan-2-yl)-2-(1,3-dioxoisoindolin-2-yl)pentane (**3b**). S48

GC–MS of 1-(2-methyl-1,3-dioxolan-2-yl)pentan-2-amine (**4b**) S49

GC–MS of 7,7,9-triethyl-1,4-dioxa-8-azaspiro[4.5]decane (**5a**) S50

GC–MS of 7,7,9-tripropyl-1,4-dioxa-8-azaspiro[4.5]decane (**5b**)....S51

EPR measurements S52

EPR spectrum of 7,7,9-triethyl-9-ethynyl-1,4-dioxa-8-azaspiro[4.5]decane-8-oxyl (**7a**) S52

EPR spectrum of 7,7,9-triethyl-9-(3-hydroxyprop-1-yn-1-yl)-1,4-dioxa-8-azaspiro[4.5]decan-8-oxyl (**7b**) S52

EPR spectrum of 7-(3-((tert-butoxycarbonyl)amino)prop-1-yn-1-yl)-7,9,9-triethyl-1,4-dioxa-8-azaspiro[4.5]decan-8-oxyl (**7c**) S53

EPR spectrum of 7,7,9-triprorpyl-9-(prop-2-en-1-yl)-1,4-dioxa-8-azaspiro[4.5]decane-8-oxyl (**7d**) S53

EPR spectrum of 7,7,9,9-tetraethyl-1,4-dioxa-8-azaspiro[4.5]decane-8-oxyl (**8a**) S54

EPR spectrum of 7,7,9-triethyl-9-(3-hydroxypropyl)-1,4-dioxa-8-azaspiro[4.5]decan-8-oxyl (**8b**)....S54

EPR spectrum of 7-(3-((*tert*-butoxycarbonyl)amino)propyl)-7,9,9-triethyl-1,4-dioxa-8-azaspiro[4.5]decan-8-oxyl (**8c**) S55

EPR spectrum of 7,7,9,9-tetrapropyl-1,4-dioxa-8-azaspiro[4.5]decane-8-oxyl (**8d**)....S55

EPR spectrum of 2,2,6,6-tetraethylpiperidin-4-one-1-oxyl (**9a**) S56

EPR spectrum of 2,2,6,6-tetrapropylpiperidin-4-one-1-oxyl (**9d**) S56

Table S5. Reduction rate constants for nitroxide **7a**,**d**; **8a**,**d**; **9a**,**d** S57

Experimental

Materials and methods

All commercially available solvents and reagents were of highest quality and used without further purification. Reactions were monitored by TLC carried out on Macherey-Nagel ALUGRAM Xtra SIL G/UV_{254} using UV light 254 nm, 1% aqueous permanganate and 10% solution of phosphomolybdic acid in ethanol and/or Dragendorff reagent as visualizing agents. Column chromatography was performed on Macherey-Nagel silica 60 (70–230 mesh).

^{1}H NMR spectra were recorded at 300, 400, 500 or 600 MHz on Bruker Avance AV 300, AV 400, DRX 500 and AV 600 spectrometers. ^{13}C NMR spectra were recorded at 75, 100, or 150 MHz on Bruker Avance AV 300, AV 400 and AV 600 spectrometers, as indicated next to each NMR analysis. ^{1}H and ^{13}C chemical shifts (δ) were internally referenced to the residual solvent peak. The *J* values in ^{1}H NMR spectra are given in Hz. For the NMR analysis of nitroxides, the samples were subjected to reduction using the zinc/CF_3COOH system before recording of the NMR spectra as described in [S1]. The IR spectra were recorded on a Bruker Vector 22 FT-IR spectrometer (Bruker, Billerica, MA, USA) in KBr pellets (1:150 ratio) or in neat samples. HRMS analyses were performed with a high resolution mass spectrometer DFS (Thermo Electron, Waltham, MA, USA).

[S1] Dobrynin, S. A.; Usatov, M. S.; Zhurko, I. F.; Morozov, D. A.; Polienko, Y. F.; Glazachev, Y. I.; Parkhomenko, D. A.; Tyumentsev, M. A.; Gatilov, Y. V.; Chernyak, E. I.; Bagryanskaya, E. G.; Kirilyuk, I. A. A. *Molecules* **2021,** *26,* 5761–5761. https://doi.org/10.3390/molecules26195761

Chromatography–mass spectrometric analysis was performed on an Agilent Technologies instrument, including an Agilent 6890N gas chromatograph and an Agilent 5973N chromatographic mass spectrometric system (EI, 70 eV). Separation was achieved using an HP-5MS capillary column (5% diphenyl 95% dimethylpolysiloxane; 30 m × 0.25 mm i.d. × 0.25 µm film thickness). Helium was used as the carrier gas at a constant flow rate of 1.0 mL/min. The column temperature program was as follows: initial hold at 50 °C for 2 min, ramped to 280 °C at a rate of 10 °C /min, followed by a final hold at 280 °C for 30 min. The injector temperature was set to 280 °C. The ion source temperature was maintained at 230 °C. Mass spectra were acquired in scan mode at a rate of 2.4 scans/s, covering a mass range of *m*/*z* 30–650.

EPR experiments were performed at room temperature (approx. 20–22 °C) on a CW EPR X-band spectrometer Bruker ER-200D (9.87 GHz). Spectra were recorded in oxygen-free conditions (via bubbling with argon) using the following settings: microwave power 5 mW,

modulation amplitude 0.8 G; time constant 50 ms; conversion time 50.12 ms. Kinetics were recorded at modulation amplitude of 1.25 G. For kinetic measurements, two stock solutions were prepared: (1) 300 mM ascorbic acid and 10 mM GSH; (2) 10 mM of GSH. All solutions were prepared in deoxygenated phosphate-citrate-borate buffer (0.5 mM × 3) at pH 7.5 with 30% of ethanol (v/v). At such contents of ethanol all radicals are totally soluble. The phosphate-citrate-borate buffer was prepared by a 100-fold dilution of a mixture containing 1 mL of 50 mM disodium phosphate solution, 1 mL of 50 mM sodium acetate solution, and 1 mL of 50 mM sodium borate solution, followed by titration with phosphoric acid to pH 7.5.

Nitroxides were prepared in stock solution (20 mM in DMSO). Just before the experiment a small aliquot of radical (to provide finally 0.2 mM) was added in the solution, quickly mixed, and placed into the EPR capillary (50 μL). Oxygen-free conditions were kept permanently. Capillaries were sealed and placed into the EPR resonator. The decay of amplitude of the low field component of the EPR spectrum was followed in kinetics measurements. The first order rate constants were calculated as the normalized initial slopes of kinetics. The kinetics measurements were performed at ascorbate concentrations of 50, 100 and 150 mM. The calculated first reaction constants were plotted versus ascorbate concentration and fitted with linear dependence. The slope corresponds to second order reaction rate constant.

Synthesis

General method of Michael reaction

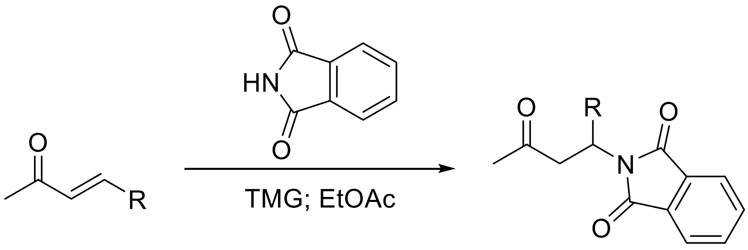

1a R= Et
1b R=Pr

2a 85%
2b 97%

A mixture of α,β-unsuturated ketone (0.1 mol), phthalimide (14.71 g, 0.1 mol), 1,1,3,3-tetramethylguanidine (1.15 g, 0.01 mol) and 100 mL ethyl acetate was heated under reflux for 3 days. Then, the mixture was cooled by freezer to −18 °C, followed by filtration to remove the precipitate. The filtrate was washed with sodium dihydrogen phosphate solution (1 M), dried with Na_2SO_4 and filtered off. After that, the solvent was evaporated and the product was used for the next step without purification.

3-(1,3-Dioxoisoindolin-2-yl)-hexan-5-one (**2a**)

Yellow oil 20.85g (85%); IR(neat) ν_{max}: 1708 (C=O). HRMS (EI/DFS) m/z: [M]+ calcd for $C_{14}H_{15}NO_3$ 245.1047, found 245.1048; ^{1}H NMR (400 MHz; $CDCl_3$, δ): 0.79 (t, J_t=7.4 Hz, 3H), 1.67 (ddq, J_{d1}=13.5 Hz, J_{d2}=5.5 Hz, J_q=7.4 Hz, 1H), 1.90 (ddq, J_{d1}=10.0 Hz, J_{d2}=13.5 Hz, J_q=7.4 Hz, 1H), 2.05 (s, 3H), 2.89 (dd, J_{d1}=17.7 Hz, J_{d2}=5.7 Hz, 1H), 3.25 (dd, J_{d1}=17.7 Hz, J_{d2}=8.8 Hz, 1H), 4.54 (dddd, J_{d1}=10.0 Hz, J_{d2}=8.8 Hz, J_{d3}=5.7 Hz, J_{d4}=5.5 Hz, 1H), 7.60–7.66 (m, 2H), 7.70–7.76 (m, 2H). ^{13}C {^{1}H} NMR (100 MHz; $CDCl_3$, δ): 10.5, 25.4, 29.9, 45.2, 48.2, 122.9, 131.5, 133.7, 168.2, 205.7.

4-(1,3-Dioxoisoindolin-2-yl)-heptan-6-one (**2b**)

Yellow oil 25.13 g (97%); IR(neat) ν_{max}: 1708 (C=O); ^{1}H NMR (500 MHz; $CDCl_3$, δ): 0.83 (t, J_t=7.4 Hz, 3H), 1.13–1.29 (m, 2H), 1.57 (dddd, J_{d1}=13.7 Hz, J_{d2}=9.9 Hz, J_{d3}=6.5 Hz, J_{d4}=5.1 Hz, 1H), 1.93 (dddd, J_{d1}=13.7 Hz, J_{d2}=9.9 Hz, J_{d3}= 9.2 Hz, J_{d4}=5.4 Hz, 1H), 2.06 (s, 3H), 2.89 (dd, J_{d1}=17.5 Hz, J_{d2}=5.8 Hz, 1H), 3.24 (dd, J_{d1}=17.5 Hz, J_{d2}=8.6 Hz, 1H), 4.65 (dddd, J_{d1}=9.9 Hz, J_{d2}=8.6 Hz, J_{d3}=5.8 Hz, J_{d4}=5.4 Hz, 1H), 7.60–7.66 (m,2H), 7.70–7.76 (m, 2H). ^{13}C {^{1}H} NMR (125 MHz; $CDCl_3$, δ): 13.3, 19.3, 29.9, 34.4, 45.5, 46.5, 122.9, 131.6, 133.7, 168.2, 205.6. Anal. Calcd for $C_{15}H_{17}NO_3$: C, 69.48; H, 6.61; N, 5.40; found: C, 69.23; H, 6.62; N, 5.51.

Dioxolanation

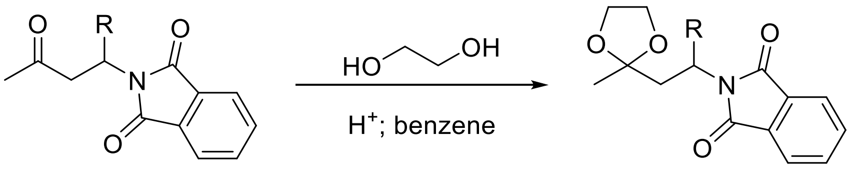

2a R = Et
2b R = Pr

3a 92%
3b 98%

A mixture of γ-(1,3-dioxoisoindolin-2-yl)ketone (0.05 mol), ethylene glycol (3.3 mL, 0.06 mol), *p*-toluenesulfonic acid dihydrate (0.31 g, 1.5 mmol) and benzene (100 mL) was placed into a Dean–Stark apparatus and heated under reflux until water stops separating. The mixture was washed with sodium bicarbonate solution, dried with Na_2SO_4 and filtered off. After that, the solvent was evaporated and the product was used for the next step without purification.

1-(2-Methyl-1,3-dioxolan-2-yl)-2-(1,3-dioxoisoindolin-2-yl)butane (**3a**)

Yellow oil 13.30 g (92%); IR(neat) ν_{max}: 1706 (C=O); HRMS (EI/DFS) m/z: [M-15]+ calcd for $C_{15}H_{16}NO_4$ 274.1071, found 274.1074; ^{1}H NMR (500 MHz; $CDCl_3$, δ): 0.81 (t, J_t=7.4 Hz, 3H), 1.25 (s, 3H), 1.68 (ddq, J_{d1}=13.8 Hz, J_{d2}=5.6 Hz, J_q=7.4 Hz, 1H), 1.83 (dd, J_{d1}=14.9

Hz, J_{d2}=2.8 Hz, 1H), 1.99 (ddq, J_{d1}=13.8 Hz, J_{d2}=10.2 Hz, J_q=7.4 Hz, 1H), 2.67 (dd, J_{d1}=14.9 Hz, J_{d2}=10.8 Hz, 1H), 3.68–3.83 (m, 4H), 4.31 (dddd, J_{d1}=10.8 Hz, J_{d2}=10.2 Hz, J_{d3}=5.6 Hz, J_{d4}=2.8 Hz, 1H), 7.59–7.68 (m, 2H), 7.72–7.79 (m, 2H). ^{13}C {^{1}H} NMR (125 MHz; $CDCl_3$, δ): 10.7, 23.7, 26.3, 39.0, 48.7, 64.0, 64.5, 109.0, 122.8, 131.9, 133.5, 168.7.

1-(2-Methyl-1,3-dioxolan-2-yl)-2-(1,3-dioxoisoindolin-2-yl)pentane (**3b**)

Yellow oil 14.87 g (98%); IR(neat) ν_{max}: 1706 (C=O); Anal. Calcd for $C_{17}H_{21}NO_4$: C, 67.31; H, 6.98; N, 4.62; found: C, 67.45; H, 7.10; N, 4.63; ^{1}H NMR (600 MHz; $CDCl_3$, δ): 0.84(t, J_t=7.4 Hz, 3H), 1.21 (qddd, J_q=7.4 Hz, J_{d1}=13.7 Hz, J_{d2}=9.3 Hz, J_{d3}=6.2 Hz, 1H), 1.24 (qddd, J_q=7.4 Hz, J_{d1}=13.7 Hz, J_{d2}=7.0 Hz, J_{d3}=8.9 Hz, 1H), 1.24 (s, 3H), 1.58 (dddd, J_{d1}=7.0 Hz, J_{d2}=9.3 Hz, J_{d3}=13.6 Hz, J_{d4}=5.5 Hz, 1H), 1.82 (dd, J_{d1}=15.0 Hz, J_{d2}=2.8 Hz, 1H), 2.01 (dddd, J_{d1}=8.9 Hz, J_{d2}=6.2 Hz, J_{d3}=13.6 Hz, J_{d4}=10.1 Hz, 1H), 2.68 (dd, J_{d1}=15.0 Hz, J_{d2}=10.6 Hz, 1H), 3.70–3.74 (m, 2H), 3.75–3.82 (m, 2H), 4.42 (dddd, J_{d1}=5.5 Hz, J_{d2}=2.8 Hz, J_{d3}=10.1 Hz, J_{d4}=10.6 Hz, 1H), 7.61–7.65 (m, 2H), 7.73–7.77 (m, 2H). ^{13}C {^{1}H} NMR (150 MHz; $CDCl_3$, δ): 13.4, 19.4, 23.7, 35.3, 39.2, 46.8, 64.0, 64.5, 109.0, 122.7, 131.9, 133.5, 168.7.

General method of hydrazinolysis

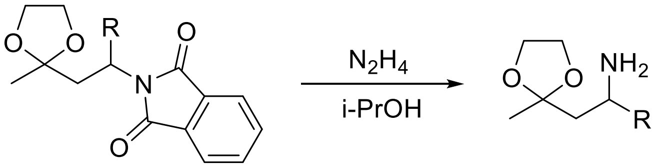

3a R = Et
3b R = Pr

4a 63%
4b 73%

A mixture of phthalimide derivative (0.05 mol), hydrazine hydrate (4.8 mL, 0.1 mol) and 2-propanol (100 mL) was heated at 60 °C for 3 days. The slurry was filtered, and 2-propanol was evaporated. The product was purified by steam distillation, followed by extraction with ether. The ethereal solution was dried with Na_2CO_3 and filtered off. After that, the solvent was evaporated and the residue was distilled in vacuo.

1-(2-Methyl-1,3-dioxolan-2-yl)butan-2-amine (**4a**)

Colorless oil 5.05g (63%); b.p. 96–98 °C at 26 mmHg; IR(neat) ν_{max}: 1039 (C-O-C); ^{1}H NMR (500 MHz; $CDCl_3$, δ): 0.71 (t, J_t=7.4 Hz, 3H), 1.13 (s, 3H), 1.13 (qdd, J_q=7.4 Hz, J_{d1}=13.4 Hz, J_{d2}=7.4 Hz, 1H), 1.19 (qdd, J_q=7.4 Hz, J_{d1}=13.4 Hz, J_{d2}=5.9 Hz, 1H), 1.39 (dd, J_{d1}=14.5 Hz, J_{d2}=9.7 Hz, 1H), 1.58 (dd, J_{d1}=14.5 Hz, J_{d2}=2.3 Hz, 1H), 1.77–1.89 (br, 2H), 2.71 (dddd, J_{d1}=5.9

Hz, J_{d2}=7.4 Hz, J_{d3}=9.7 Hz, J_{d4}=2.3 Hz, 1H), 3.72–3.79 (m, 4H). ^{13}C {^{1}H} NMR (125 MHz; $CDCl_3$, δ): 9.9, 23.7, 30.9, 45.0, 48.5, 63.7, 64.1, 109.7.

1-(2-Methyl-1,3-dioxolan-2-yl)pentan-2-amine (**4b**)

Colorless oil 6.31g (73%); b.p. 130–140 °C at 26 mmHg; IR(neat) ν_{max}: 1043 (C-O-C); Anal. Calcd for $C_9H_{19}NO_2$: C, 62.39; H, 11.05; N, 8.08; found: C, 61.65; H, 10.73; N, 8.58.; HRMS (EI/DFS) m/z: [M-43]+ calcd for $C_6H_{12}NO_2$ 130.0836, found 130.0865; ^{1}H NMR (500 MHz; $CDCl_3$, δ): 0.81 (t, J_t=7.0 Hz, 3H), 1.14–1.32 (m, 4H), 1.24 (s, 3H), 1.45–1.54 (br, 2H), 1.49 (dd, J_{d1}=14.4 Hz, J_{d2}= 9.6 Hz, 1H), 1.66 (dd, J_{d1}=14.4 Hz, J_{d2}=2.1 Hz, 1H), 2.89 (dddd, J_{d1}=9.6 Hz, J_{d2}=2.1 Hz, J_{d3}=7.3 Hz, J_{d4}=5.8 Hz, 1H), 3.81–3.89 (m, 4H). ^{13}C {^{1}H} NMR (125 MHz; $CDCl_3$, δ): 13.9, 18.9, 23.9, 40.8, 45.8, 46.9, 63.9, 64.3, 109.9.

General method of heterocyclization

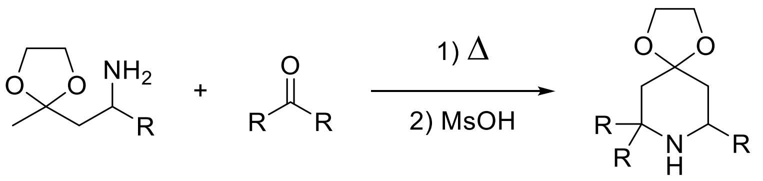

A mixture of amine (0.05 mol), ketone (0.06 mol), and pyridinium *p*-toluenesulfonate (1.25 g, 0.005 mol) in benzene (500 mL) was heated under reflux using a Dean–Stark apparatus for 3 d. Methanesulfonic acid (9.6 g, 0.1 mol) was then added to the boiling mixture and heated under reflux for 1 h. The mixture was washed with a saturated sodium carbonate solution (500 mL). The organic phase was separated, and the solvent was evaporated. The residue was distilled in vacuo.

7,7,9-Triethyl-1,4-dioxa-8-azaspiro[4.5]decane (**5a**)

Colorless oil 5.8g (51%) b.p. 101–110 °C at 3.5 mmHg; IR(neat) ν_{max}: 2962 (C-H), 1068 (C-O-C); HRMS (EI/DFS) m/z: [M-29]+ calcd for $C_{11}H_{20}NO_2$ 198.1489, found 198.1486; ^{1}H NMR (400 MHz; $CDCl_3$, δ): 0.67 (t, J_t=7.4 Hz, 3H), 0.75 (t, J_t=7.4 Hz, 3H), 0.86 (t, J_t=7.4 Hz, 3H), 1.12 (dd, J_{d1}=12.8 Hz, J_{d2}=11.9 Hz, 1H), 1.21 (dq, J_d=13.8 Hz, J_q=7.4 Hz, 1H), 1.29 (ddq, J_{d1}=14.2 Hz, J_{d2}=6.5 Hz, J_q=7.4 Hz, 1H), 1.32 (d, Jd=12.7 Hz, 1H), 1.33 (ddq, J_{d1}=14.2 Hz, J_{d2}=6.5 Hz, J_q=7.4 Hz, 1H), 1.33 (dq, J_d=13.8 Hz, J_q=7.4 Hz, 1H), 1.39 (dq, J_d=14.9 Hz, J_q=7.4 Hz, 1H), 1.62 (dd, J_{d1}=12.8 Hz, J_{d2}=2.3 Hz, 1H), 1.67 (ddd, J_{d1}=12.7 Hz, J_{d2}=2.4 Hz, J_{d3}=2.1 Hz, 1H), 1.82 (dq, J_d=14.9 Hz, J_q=7.4 Hz, 1H), 2.67 (dddd, J_{d1}=11.9 Hz, J_{d2}= 2.3 Hz, J_{d3}=6.5 Hz,

J_{d4}=6.5 Hz, 1H), 3.79–3.85 (m, 2H), 3.87–3.93 (m, 2H). $^{13}C\{^1H\}$ NMR (100 MHz; $CDCl_3$, δ): 7.29, 7.31, 10.3, 23.5, 29.6, 33.2, 41.6, 42.3, 49.2, 54.9, 63.2, 64.5, 108.8.

7,7,9-Tripropyl-1,4-dioxa-8-azaspiro[4.5]decane (**5b**)

Colorless oil 6.9g (50%) b.p. 135–140 °C at 3.5 mmHg; IR(neat) ν_{max}: 2956 (C-H), 1070 (C-O-C); Anal. Calcd for $C_{16}H_{31}NO_2$: C, 71.33; H, 11.60; N, 5.20; found: C, 71.55; H, 11.68; N, 5.20.; HRMS (EI/DFS) m/z: [M-43]+ calcd for $C_{13}H_{24}NO_2$ 226.1802, found 226.1799; ^1H NMR (300 MHz; $CDCl_3$, δ): 0.82–0.69 (m, 9H), 1.03–1.51 (m, 15H), 1.63–1.83 (m,3H), 2.85 (dddd, J_{d1}=11.5 Hz, J_{d2}=5.9 Hz, J_{d3}=5.9 Hz, J_{d4}=2.4 Hz, 1H), 3.83–3.91 (m, 2H), 3.92–4.00 (m, 2H). ^{13}C $\{^1H\}$ NMR (75 MHz; $CDCl_3$, δ): 13.7, 14.29, 14.34, 15.8, 16.3, 18.7, 34.3, 38.9, 41.8, 42.7, 44.1, 47.1, 54.6, 62.9, 64.2, 108.5.

General method for 2-alkynylpiperidine-1-oxyl synthesis

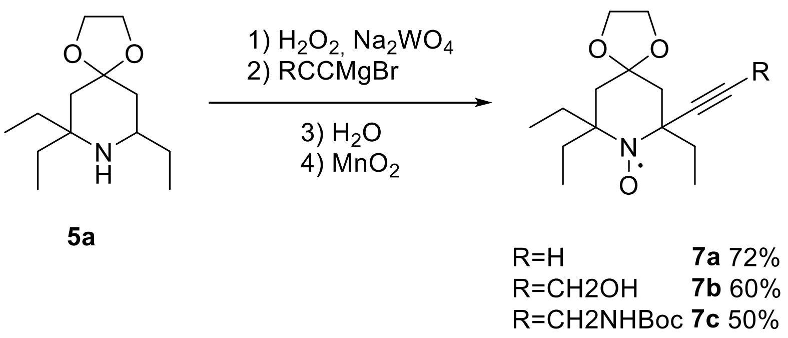

To a solution of amine **5a** (2.27 g, 0.01 mol) in methanol (50 mL), a solution of sodium tungstate (0.29 g, 0.001 mol) and Trilon B (0.34 g, 0.001 mol) in water (10 mL) was added, and water was added until a homogeneous solution formed. Then, 30% hydrogen peroxide solution (5 mL, 0.05 mol) was added to the mixture, and it was left in a dark place for 3 d. Upon completion of the reaction (monitored by TLC, silica gel, ethyl acetate), the reaction mixture was diluted with water (50 mL), and methanol was evaporated under reduced pressure. The product was extracted with dichloromethane (3 × 20 mL) and dried over sodium sulfate. The drying agent was filtered off, and the solution was evaporated under reduced pressure. To the residue, a 0.5 M solution of corresponding alkynylmagnesium bromide* in THF (200 mL, 0.1 mol) was added, and the mixture was stirred for 2 d. The reaction was then quenched with water, the organic phase was separated and dried over sodium sulfate. The drying agent was filtered off, and manganese dioxide (10 g, 0.115 mol) was added to the mixture. After completion of the oxidation, manganese dioxide was filtered off, the solvent was evaporated under reduced pressure, and the residue was chromatographed.

7,7,9-Triethyl-9-ethynyl-1,4-dioxa-8-azaspiro[4.5]decane-8-oxyl (**7a**)

*The solution of ethynylmagnesium bromide was prepared as described in literature [S2].

Pink crystals 1.9 g (72%). m.p. 53–55 °C; IR(KBr) ν_{max}: 3259 (≡C-H), 2106 (C≡C); Anal. Calcd for $C_{15}H_{24}NO_3$: C, 67.64; H, 9.08; N, 5.26. Found: C, 68.28; H, 9.11; N, 5.28.; HRMS (EI/DFS) m/z: [M]+ calcd for $C_{15}H_{24}NO_3$ 266.1751, found 266.1754. 1H NMR (400 MHz, CD_3OD, Zn/CF_3COOH system): 0.97 (t, J_t=7.4 Hz, 3H), 0.98 (t, J_t=7.4 Hz, 3H), 1.14 (t, J_t=7.4 Hz, 3H), 1.73 (dq, J_d=14.4 Hz, J_q=7.4 Hz, 1H), 1.77 (d, J_d=14.5 Hz, 1H), 1.85 (d, J_d=14.7 Hz, 1H), 1.87 (dq, J_d=14.4 Hz, J_q=7.4 Hz, 1H), 1.99 (dq, J_d=12.7 Hz, J_q=7.4 Hz, 1H), 2.07 (dq, J_d=12.7 Hz, J_q=7.4 Hz, 1H), 2.15 (dd, J_d=14.7 Hz, J_d=2.3 Hz, 1H), 2.35 (dq, J_d=15.3 Hz, J_q=7.4 Hz, 1H), 2.35 (dd, J_d=14.5 Hz, J_d=2.3 Hz, 1H), 2.57 (dq, J_d=15.3 Hz, J_q=7.4 Hz, 1H), 3.29 (s, 1H), 3.33 (ddd, J_{d1}=7.7 Hz, J_{d2}=6.5 Hz, J_{d3}=6.7 Hz, 1H), 3.95 (ddd, J_{d1}=7.7 Hz, J_{d2}=6.8 Hz, J_{d3}=5.5 Hz, 1H), 4.08 (ddd, J_{d1}=7.9 Hz, J_{d2}=6.8 Hz ,J_{d3}=6.5 Hz, 1H), 4.09 (ddd, J_{d1}=7.9 Hz, J_{d2}=6.7 Hz, J_{d3}=5.5 Hz, 1H).

[S2] Skattebol L., Jones E. R. H., Whiting M. C. 1-PHENYL-1-PENTEN-4-YN-3-OL. Organic Syntheses 1959, 39, 56. https://doi.org/10.15227/orgsyn.039.0056.

7,7,9-Triethyl-9-(3-hydroxyprop-1-yn-1-yl)-1,4-dioxa-8-azaspiro[4.5]decan-8-oxyl (**7b**)

*The solution of alkynylmagnesium bromide was prepared by addition of 100 mL of a 2 M ethylmagnesium bromide (0.2 mol) solution in THF to a solution of propargyl alcohol (5.6 g, 0.1 mol) in 100 mL THF.

Yellow oil 1.8 g (60%). IR (neat) ν_{max}: 3410 (O-H), 2970 (C-H), 2939 (C-H), 2883 (C-H); Anal. Calcd for $C_{16}H_{26}O_4N$: C, 64.34; H, 8.84; N, 4.73; found: C, 64.19; H, 8.53; N, 4.62; HRMS (EI/DFS) m/z $[M-28]^+$ Calcd for $C_{14}H_{22}O_4N$: 268.1543; found: 268.1541. ^{1}H NMR (300 MHz, CD_3OD, Zn/CF_3COOH, δ (ppm)): 0.97 (t, J_t=7.3 Hz, 3H), 0.97 (t, J_t=7.3 Hz, 3H), 1.13 (t, J_t=7.3 Hz, 3H), 1.72 (dq, J_d=14.4 Hz, J_q=7.3 Hz, 1H), 1.76 (d, J_d=14.7 Hz, 1H), 1.85 (d, J_d=14.4 Hz, 1H), 1.87 (dq, J_d=14.4 Hz, J_q=7.3 Hz, 1H), 1.98 (dq, J_d=12.6 Hz, J_q=7.3 Hz, 1H), 2.07 (dq, J_d=12.6 Hz, J_q=7.3 Hz, 1H), 2.14 (d, J_d=14.7 Hz, 1H), 2.30 (dq, J_d=14.7 Hz, J_q=7.3 Hz, 1H), 2.33 (d, J_d=14.4 Hz, 1H), 2.57 (dq, J_d=14.7 Hz, J_q=7.3 Hz, 1H), 4.42 (d, J_d=6.3 Hz, 2H), 4.51 (d, J_d=6.3 Hz, 2H), 4.28 (s, 2H).

7-(3-((*tert*-Butoxycarbonyl)amino)prop-1-yn-1-yl)-7,9,9-triethyl-1,4-dioxa-8-azaspiro[4.5]decan-8-oxyl (**7c**)

*The solution of alkynylmagnesium bromide was prepared by addition of 100 mL of a 2 M ethylmagnesium bromide (0.2 mol) solution in THF to a solution of *N*-Boc-propargylamine (15.5 g, 0.1 mol) in 100 mL THF.

Yellow oil 2.0 g (50%). IR (neat) ν_{max}: 3348 (N-H), 2975 (C-H), 2937 (C-H), 2881 (C-H), 1719 (C=O); Anal. Calcd for $C_{21}H_{35}O_5N_2$: C, 63.77; H, 8.92; N, 7.08; Found: C, 63.97; H, 9.18; N, 7.22; HRMS (EI/DFS) m/z $[M]^+$ Calcd for $C_{21}H_{35}O_5N_2$: 395.2541; found: 395.2542. ^{1}H NMR (300 MHz, CD_3OD, Zn/CF_3COOH, δ (ppm)): 0.96 (t, J_t=7.3 Hz, 3H), 0.96 (t, J_t=7.3 Hz, 3H), 1.10 (t, J_t=7.3 Hz, 3H), 1.46 (s, 9H), 1.72 (dq, J_d=14.4 Hz, J_q=7.3 Hz, 1H), 1.75 (d, J_d=14.7 Hz, 1H), 1.83 (d, J_d=14.3 Hz, 1H), 1.85 (dq, J_d=14.4 Hz, J_q=7.3 Hz, 1H), 1.95 (dq, J_d=12.6 Hz, J_q=7.3 Hz, 1H), 2.05 (dq, J_d=12.6 Hz, J_q=7.3 Hz, 1H), 2.12 (d, J_d=14.7 Hz, 1H), 2.35 (dq, J_d=15.3 Hz, J_q=7.3 Hz, 1H), 2.30 (d, J_d=14.3 Hz, 1H), 2.48 (dq, J_d=15.3 Hz, J_q=7.3 Hz, 1H), 3.89 (s, 2H), 3.93 (d, J_d=6.3 Hz, 2H), 4.08 (d, J_d=6.3 Hz, 2H).

7,7,9-Tripropyl-9-(prop-2-en-1-yl)-1,4-dioxa-8-azaspiro[4.5]decane-8-oxyl (**7d**)

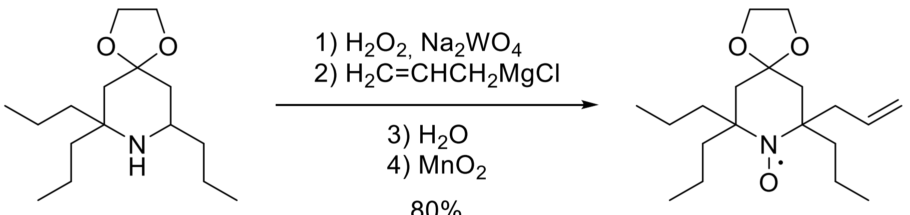

5b **7d**

To a solution of amine **5b** (2.7 g, 0.01 mol) in methanol (50 mL), a solution of sodium tungstate (0.29 g, 0.001 mol) and Trilon B (0.34 g, 0.001 mol) in water (10 mL) was added, and water was added until a homogeneous solution formed. Then, 30% hydrogen peroxide solution (5 mL, 0.05 mol) was added to the mixture, and it was left in a dark place for 3 d. Upon completion of the reaction (monitored by TLC, silica gel, ethyl acetate), the reaction mixture was diluted with water (50 mL), and methanol was evaporated under reduced pressure. The product was extracted with dichloromethane (3 × 20 mL) and dried over sodium sulfate. The drying agent was filtered off, and the solution was evaporated under reduced pressure. To the residue, a 2 M solution of allylmagnesium chloride in THF (50 mL, 0.1 mol) was added, and the mixture was stirred for 1 h. The reaction was then quenched with water, the organic phase was separated and dried over sodium sulfate. The drying agent was filtered off, and manganese dioxide (10 g, 0.115 mol) was added to the mixture. After completion of the oxidation, manganese dioxide was filtered off, the solvent was evaporated under reduced pressure, and the residue was

chromatographed. Red oil 2.6 g were obtained (80%). IR(KBr) ν_{max}: 3076 (sp^{2}C-H), 1637 (C=C); Anal. Calcd for $C_{19}H_{34}NO_3$: C, 70.33; H, 10.56; N, 4.32. Found: C, 70.38; H, 10.23; N, 4.27.; HRMS (EI/DFS) m/z: $[M]^+$ calcd for $C_{19}H_{34}NO_3$ 324.2533, found 324.2531; ^{1}H NMR (300 MHz, CD_3OD, Zn/CF_3COOH system): 0.96 (t, J_t=7.4 Hz, 3H), 0.98 (t, J_t=7.4 Hz, 3H), 0.98 (t, J_t=7.4 Hz, 3H), 1.28–1.56 (m, 6H), 1.74–2.06 (m, 10H), 2.77 (d, J_d=7.2 Hz, 2H), 3.94–4.06 (m, 4H), 5.27 (dd, J_{d1}=17.1 Hz, J_{d2}=1.8 Hz, 1H), 5.28 (dd, J_{d1}=9.9 Hz, J_{d2}=1.8 Hz, 1H), 5.83 (ddt, J_{d1}=17.1 Hz, J_{d2}=9.9 Hz, J_t=7.3 Hz, 1H)

7,7,9,9-Tetraethyl -1,4-dioxa-8-azaspiro[4.5]decane-8-oxyl (**8a**)

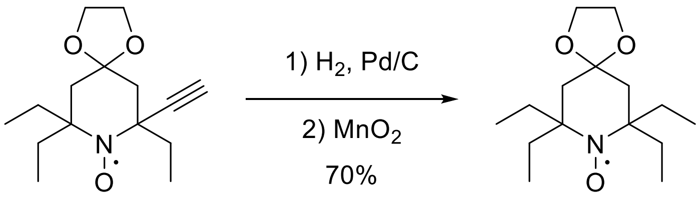

7a **8a**

To a solution of nitroxide **7a** (1.33 g, 0.005 mol) in THF (50 mL), 4% palladium on carbon (100 mg) was added, and the mixture was hydrogenated with stirring at room temperature and atmospheric pressure until hydrogen uptake ceased. The catalyst was then filtered off.

(*Optional dioxolan cleavage*): Concentrated hydrochloric acid (10 mL) was added to the filtrate, and the mixture was allowed to stand for 2 d. After completion of the reaction, the mixture was diluted with water (50 mL), and THF was evaporated under reduced pressure. The reaction mixture was neutralized with sodium carbonate and extracted with ether (3 × 25 mL). The organic phase was separated and dried over sodium carbonate.

To the solution (ethereal or residual THF after hydrogenation if cleavage omitted) was added manganese dioxide (4.35 g, 50.0 mmol). Upon completion of the oxidation, the manganese dioxide was filtered off, and the solvent was evaporated under reduced pressure. The residue was chromatographed.

7,7,9,9-Tetraethyl-1,4-dioxa-8-azaspiro[4.5]decane- 8-oxyl (**8a**)

Orange crystals 0.8 g were obtained (70%). m.p. 42–43 °C; IR(KBr) ν_{max}: 2966 (C-H); Anal. Calcd for $C_{15}H_{28}NO_3$: C, 66.63; H, 10.44; N, 5.18. Found: C, 67.01; H, 10.58; N, 5.21.; HRMS (EI/DFS) m/z: [M]+ calcd for $C_{15}H_{28}NO_3$ 270.2064, found 270.2061.; ^{1}H NMR (400 MHz, CD_3OD, Zn/CF_3COOH system): 0.97 (t, J_t=7.4 Hz, 12H), 1.94 (dq, J_d=14.4 Hz, J_q=7.4 Hz, 4 H), 1.95 (s, 4H), 2.05 (dq, J_d=14.4 Hz, J_q=7.4 Hz, 4H), 4.00 (s, 4H).

7,7,9-triethyl-9-(3-hydroxypropyl)-1,4-dioxa-8-azaspiro[4.5]decan-8-oxyl (**8b**)

To a solution of nitroxide **7b** (1.5 g, 0.005 mol) in THF (50 mL), 4% palladium on carbon (100 mg) was added, and the mixture was hydrogenated with stirring at room temperature and atmospheric pressure until hydrogen uptake ceased. The catalyst was then filtered off. To the solution (ethereal or residual THF after hydrogenation if cleavage omitted) was added manganese dioxide (4.35 g, 50.0 mmol). Upon completion of the oxidation, the manganese dioxide was filtered off, and the solvent was evaporated under reduced pressure. The residue was chromatographed. Yellow oil 0.45 g (30%). IR (neat) ν_{max}: 3441 (O-H), 2964 (C-H), 2941 (C-H), 2881 (C-H); Anal. Calcd for $C_{16}H_{30}O_4N$: C, 63.97; H, 10.07; N, 4.66; Found: C, 64.05; H, 10.09; N, 4.60; HRMS (EI/DFS) m/z $[M]^+$ Calcd for $C_{16}H_{30}O_4N$: 300.2169; found: 300.2164. ^{1}H NMR (4 00 MHz, CD_3OD, Zn/CF_3COOH, δ (ppm)): 0.97 (t, J_t=7.0 Hz, 9H), 1.66 (ddddd, J_{d1}=3.9 Hz, J_{d2}=4.4 Hz, J_{d3}=7.0 Hz, J_{d4}=7.0 Hz, J_{d5}=14.0 Hz, 1H), 1.69 (ddddd, J_{d1}=6.1 Hz, J_{d2}=8.0 Hz, J_{d3}=8.0 Hz, J_{d4}=8.1 Hz, J_{d5}=14.0 Hz, 1H), 1.91 (d, J_d=15.0 Hz, 1H), 1.93 (d, J_d=11.5 Hz, 1H), 1.96 (q, J_q=7.0 Hz, 4H), 1.99 (dd, J_{d1}=7.0 Hz, J_{d2}=8.0 Hz, 2H), 2.02 (d, J_d=15.0 Hz, 1H), 2.04 (d, J_d=11.5 Hz, 1H), 2.07 (dq, J_d=7.0 Hz, J_q=14.2 Hz, 1H), 2.13 (dq, J_d=7.0 Hz, J_q=14.2 Hz, 1H), 3.62 (ddd, J_{d1}=4.4 Hz, J_{d2}=8.1 Hz, J_{d3}=11.1 Hz, 1H), 1.99 (ddd, J_{d1}=3.9 Hz, J_{d2}=6.1 Hz, J_{d3}=11.1 Hz, 1H), 3.99 (d, J_d=6.3 Hz, 2H), 4.02 (d, J_d=6.3 Hz, 2H).

7-(3-((*tert*-Butoxycarbonyl)amino)propyl)-7,9,9-triethyl-1,4-dioxa-8-azaspiro[4.5]decan-8-oxyl (**8c**)

To a solution of nitroxide **7c** (2 g, 0.005 mol) in THF (50 mL), 4% palladium on carbon (100 mg) was added, and the mixture was hydrogenated with stirring at room temperature and atmospheric pressure until hydrogen uptake ceased. The catalyst was then filtered off. To the solution (ethereal or residual THF after hydrogenation if cleavage omitted) was added manganese dioxide (4.35 g, 50.0 mmol). Upon completion of the oxidation, the manganese dioxide was filtered off, and the solvent was evaporated under reduced pressure. The residue was chromatographed. Yellow oil 0.6 g (30%). Yellow oil. IR (neat) ν_{max}: 3358 (N-H), 2972 (C-H), 2937 (C-H), 2881 (C-H), 1711 (C=O); HRMS (EI/DFS) m/z $[M]^+$ Calcd for $C_{21}H_{39}O_5N_2$: 399.2854; found: 399.2858. ^{1}H NMR (300 MHz, CD_3OD, Zn/CF_3COOH, δ) 0.96 (t, J_t=7.3 Hz, 9H), 1.44 (s, 9H), 1.47–1.58 (m, 1H); 1.85–2.10 (m, 12H), 3.08 (t, J_t=6.5 Hz, 2H), 3.95–4.05 (m, 4H).

7,7,9,9-Tetrapropyl-1,4-dioxa-8-azaspiro[4.5]decane-8-oxyl (**8d**):

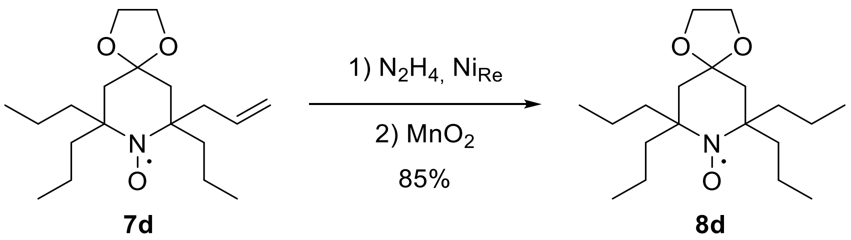

A solution of nitroxide **7d** (3.24 g, 0.01 mol) in methanol (20 mL) was treated with hydrazine hydrate (5 g, 0.1 mol) and freshly prepared skeletal nickel (ca. 200 mg). The reaction mixture was stirred at room temperature for 48 h, until gas evolution ceased. The catalyst was then filtered off.

(Optional dioxolan cleavage): Concentrated hydrochloric acid (10 mL) was added to the filtrate, and the mixture was allowed to stand for 2 d. After completion of the reaction, the mixture was diluted with water (50 mL), and the methanol was evaporated under reduced pressure. The reaction mixture was neutralized with sodium carbonate and extracted with ether (3 × 25 mL). The organic phase was separated and dried over sodium carbonate.

The mixture was filtered off and the filtrate was evaporated in vacuo. The residue was dissolved in chloroform and manganese dioxide (8.7 g, 0.1 mol) was added. The resulting mixture was stirred at room temperature until oxidation was complete (monitored by TLC). The manganese dioxide was removed by filtration, and the solvent was evaporated in vacuo. The crude product was purified by column chromatography on silica gel (eluent hexane/ethyl acetate 2 :1).

Orange oil 2.8 g (85%); IR(neat) ν_{max}: 2960 (C-H); Anal. Calcd for $C_{19}H_{36}NO_3$: C, 69.90; H, 11.11; N, 4.26. Found: C, 70.21; H, 11.02; N, 4.31.; HRMS (EI/DFS) m/z: $[M-1]^+$ calcd for $C_{19}H_{35}NO_3$ 325.2612, found 325.2614. 1H NMR (300 MHz, CD3OD, Zn/CF3COOH system): 0.90 (t, Jt=7.4 Hz, 12H), 1.31 (qdd, Jq=7.4 Hz, Jd1=7.6 Hz, Jd2=9.9 Hz, 8H), 1.77 (ddd, Jd1=14.7 Hz, Jd2=9.6 Hz, Jd3=7.6 Hz, 4H), 1.90 (s, 4H), 1.91 (ddd, Jd1=14.7 Hz, Jd2=9.9 Hz , Jd3=7.6 Hz, 4H), 3.93 (s, 4H).

2,2,6,6-Tetraethylpiperidin-4-one-1-oxyl (**9a**)

Orange crystal (58%). IR(KBr) ν_{max}: 1720 (C=O); 1H NMR (400 MHz, CD_3OD, Zn/CF_3COOH system): 1.00 (t, J_t=7.4 Hz, 12H), 1.86 (dq, J_d=14.8 Hz, J_q=7.4 Hz, 4H), 2.03 (dq, J_d=14.8 Hz, J_q=7.4 Hz, 4H), 2.75 (s, 4H).

2,2,6,6-Tetrapropylpiperidin-4-one-1-oxyl (9d)

Orange crystals 1.9g (67%); m.p. 47–51 °C; IR(KBr) ν_{max}: 1716 (C=O); Anal. Calcd for $C_{17}H_{32}NO_2$: C, 72.29; H, 11.33; N, 4.96. Found: C, 72.32; H, 11.47; N, 5.01.; HRMS (EI/DFS) m/z: $[M-1]^+$ calcd for $C_{17}H_{32}NO_2$ 282.2428, found 282.2426.; ^{1}H NMR (400 MHz, CD_3OD, Zn/CF_3COOH system): 0.95 (t, J_t=7.4 Hz, 12H), 1.27–1.47 (m, 8H), 1.56–1.95 (m, 8H), 2.73 (s, 4H).

NMR spectra

^{1}H NMR (400 MHz, $CDCl_3$) of 3-(1,3-dioxoisoindolin-2-yl)-hexanone-5 (**2a**).

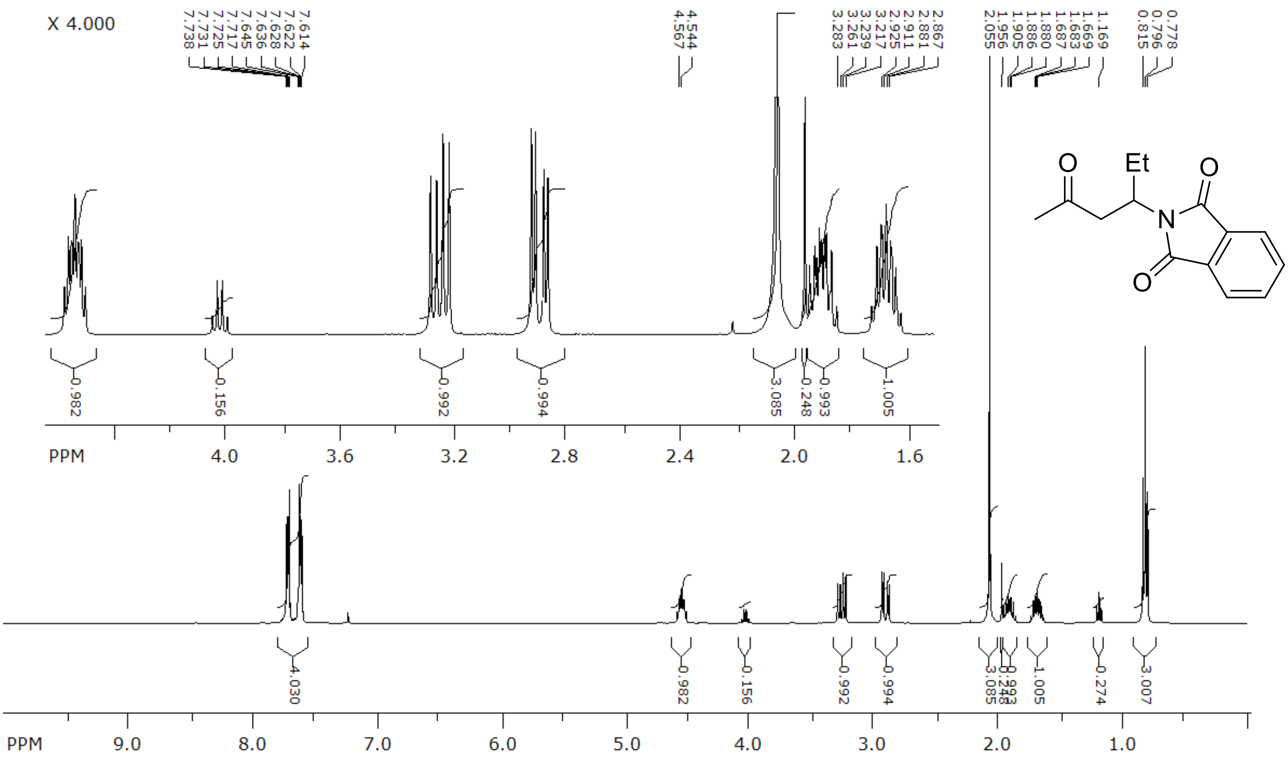

^{13}C {^{1}H} NMR (100 MHz, $CDCl_3$) of 3-(1,3-dioxoisoindolin-2-yl)-hexanone-5 (**2a**).

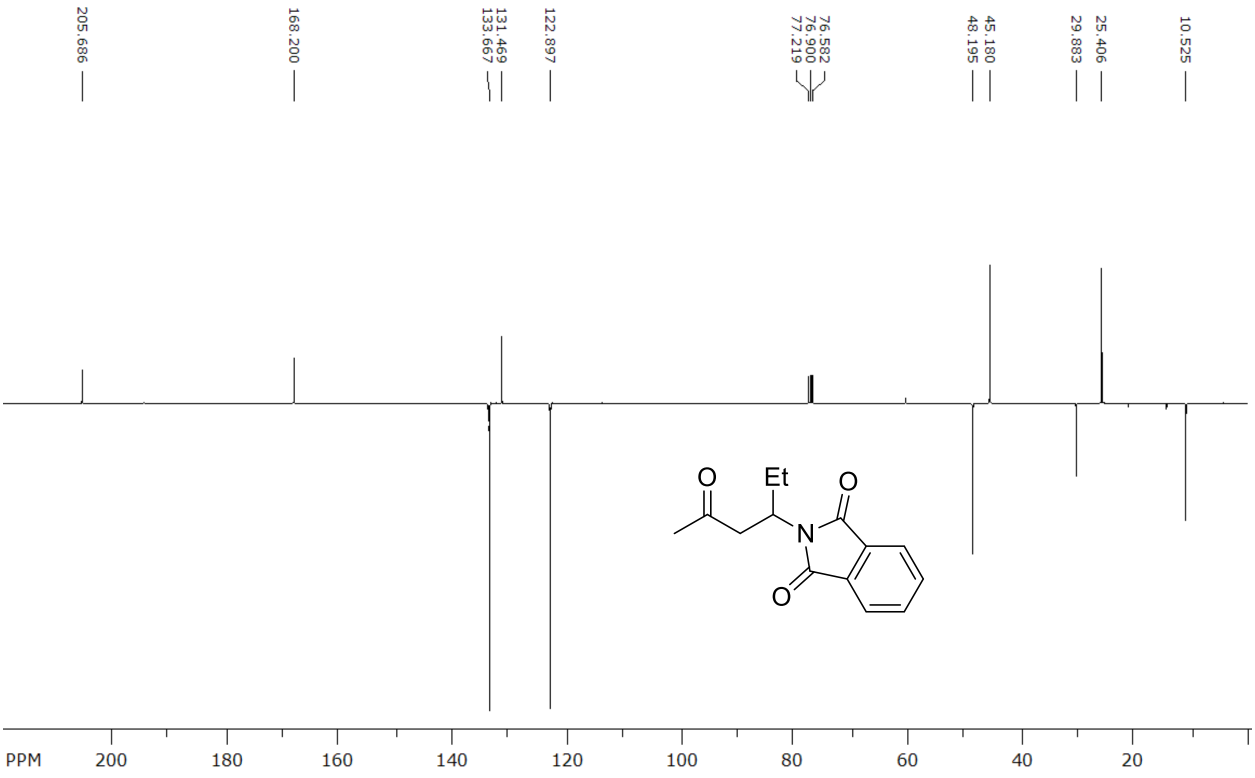

^{1}H NMR (500 MHz, $CDCl_3$) of 1-(2-methyl-1,3-dioxolan-2-yl)-2-(1,3-dioxoisoindolin-2-yl)butane (**3a**)

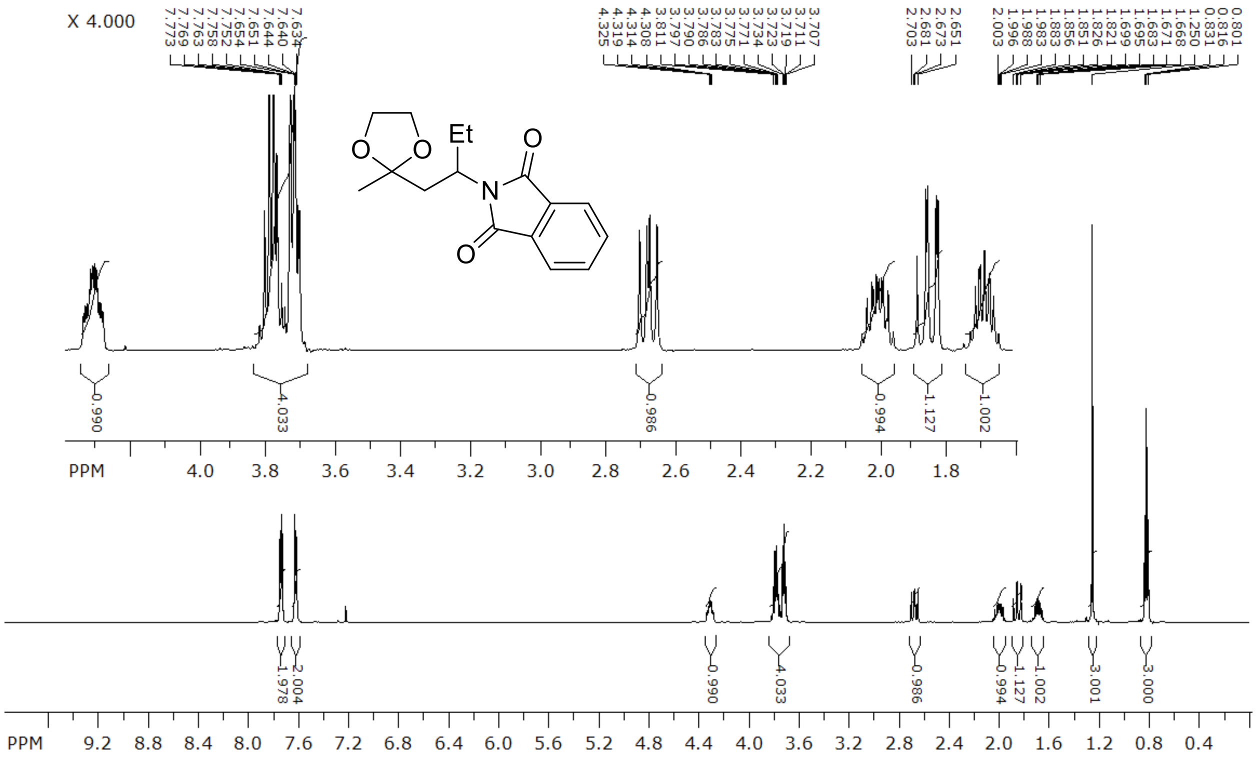

^{13}C {^{1}H} NMR (125 MHz, $CDCl_3$) of 1-(2-methyl-1,3-dioxolan-2-yl)-2-(1,3-dioxoisoindolin-2-yl)butane (**3a**)

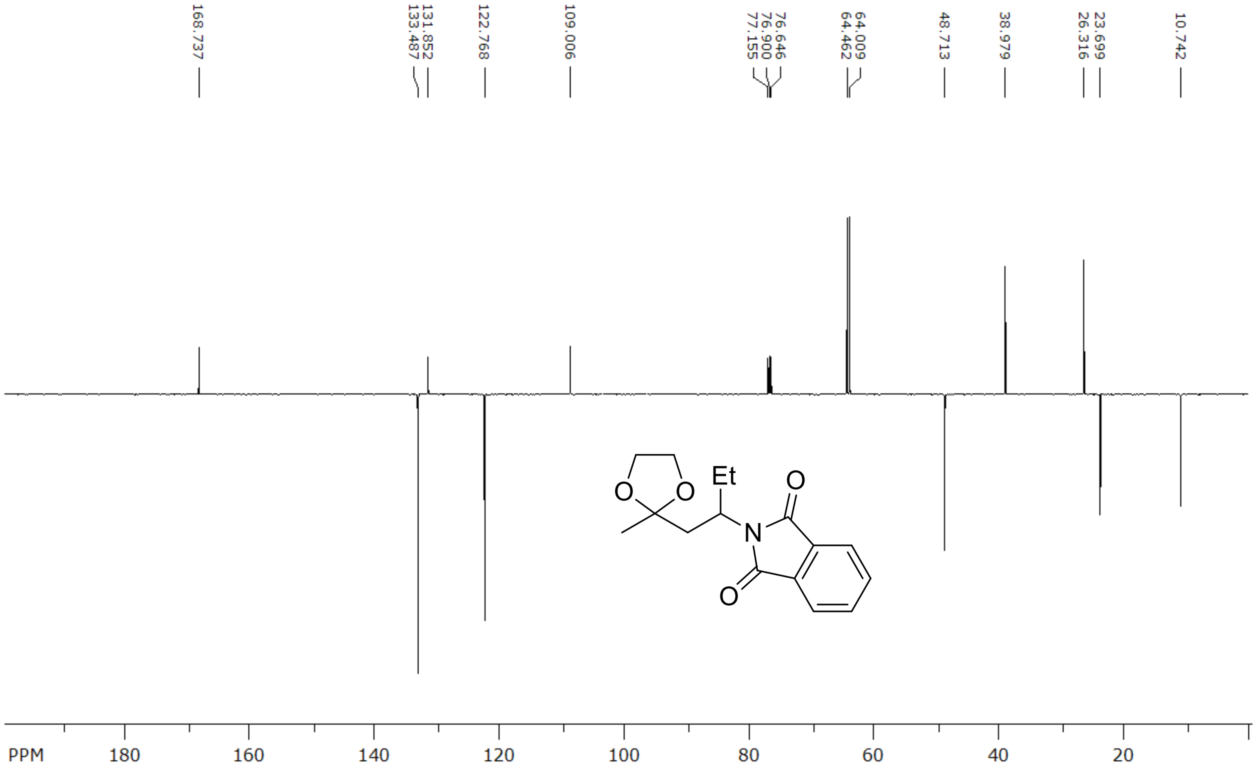

^{1}H NMR (500 MHz, $CDCl_3$) of 1-(2-methyl-1,3-dioxolan-2-yl)butan-2-amine (**4a**)

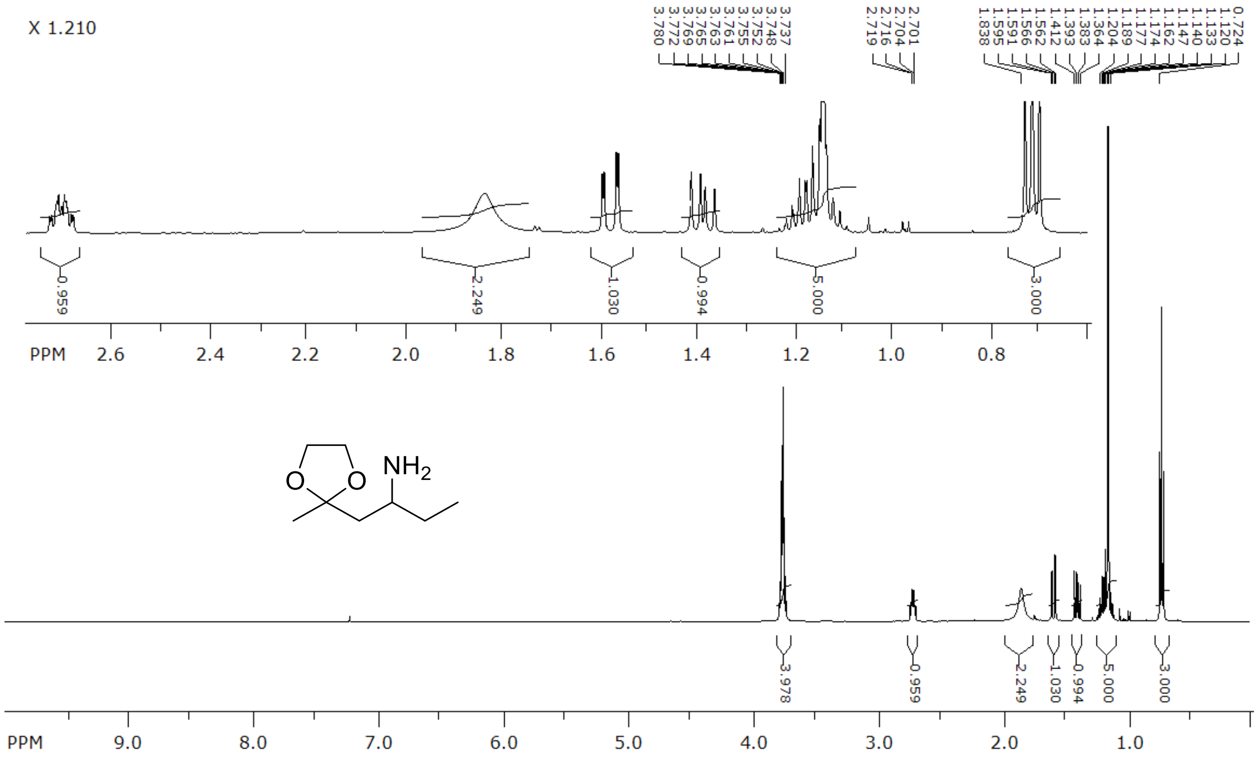

^{13}C {^{1}H} NMR (125MHz, $CDCl_3$) of 1-(2-methyl-1,3-dioxolan-2-yl)butan-2-amine (**4a**)

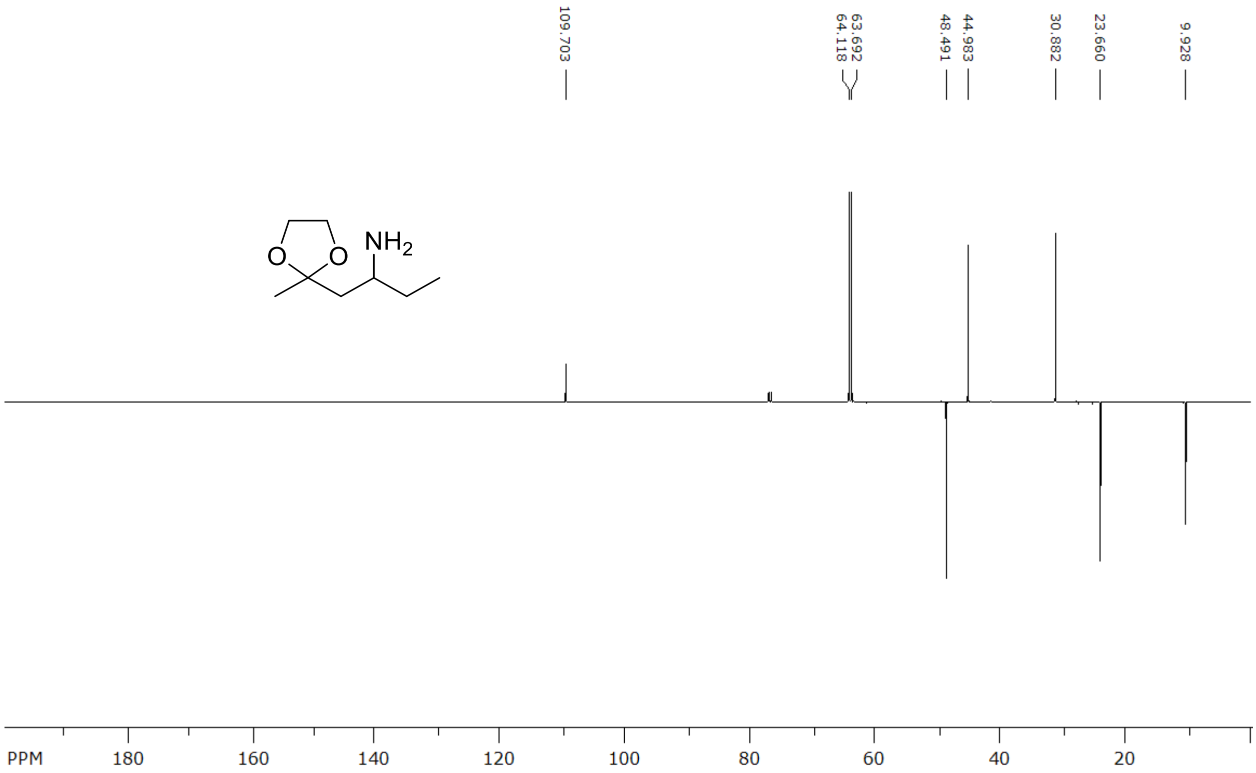

^{1}H NMR (500 MHz, $CDCl_3$) of 4-(1,3-dioxoisoindolin-2-yl)-heptanone-6 (**2b**)

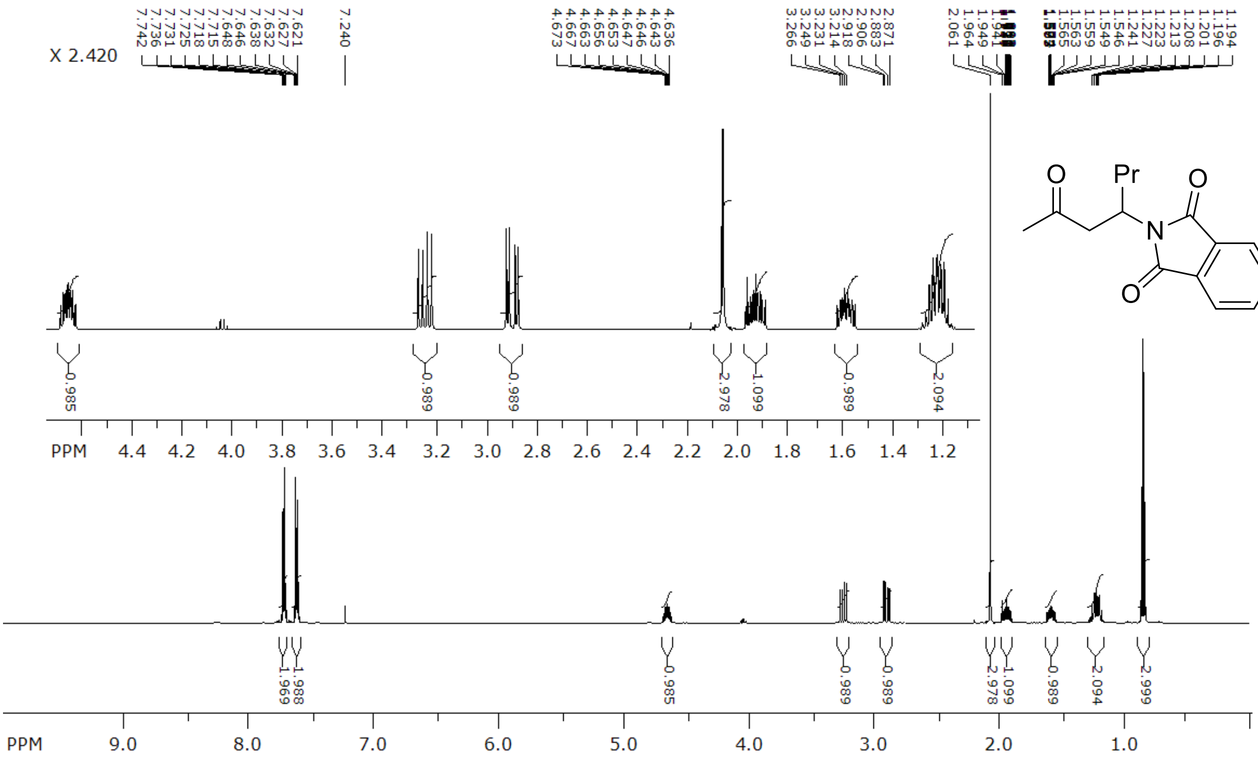

^{13}C {^{1}H} NMR (125 MHz, $CDCl_3$) of 4-(1,3-dioxoisoindolin-2-yl)-heptanone-6 (**2b**)

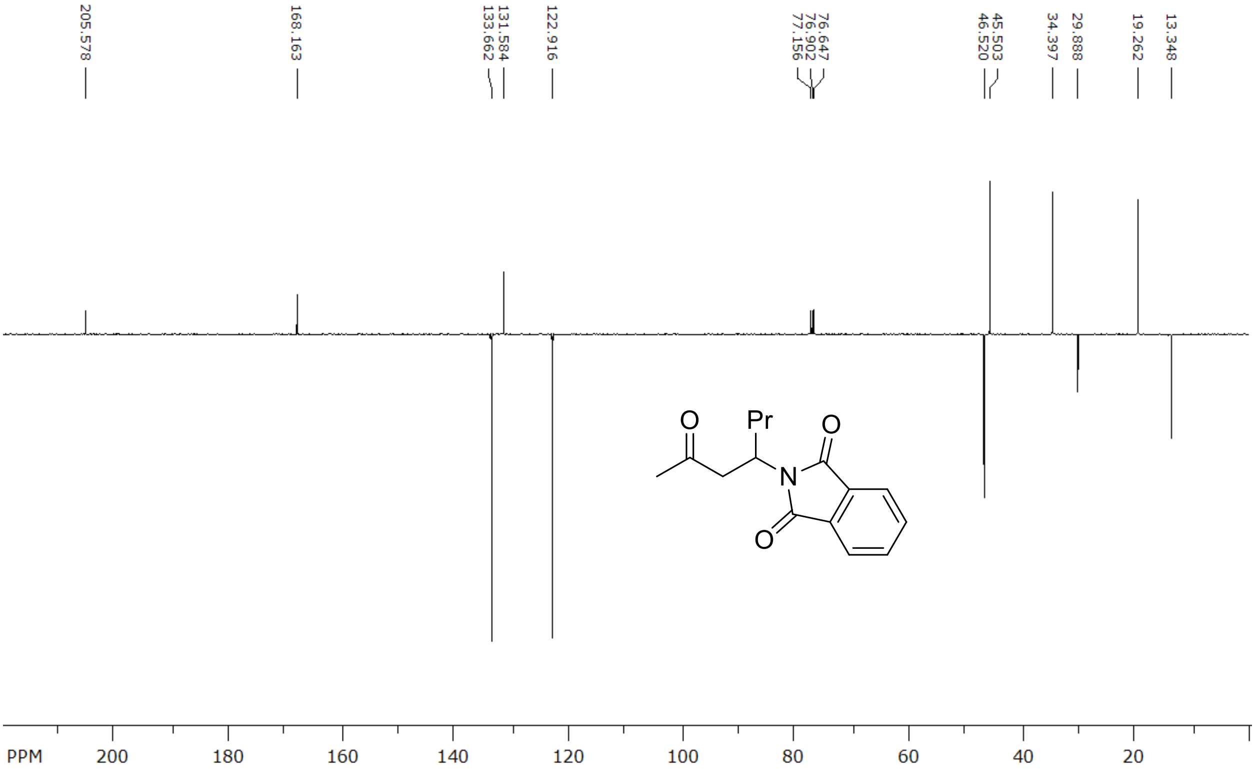

^{1}H NMR (600 MHz, $CDCl_3$) of 1-(2-methyl-1,3-dioxolan-2-yl)-2-(1,3-dioxoisoindolin-2-yl)pentane (**3b**)

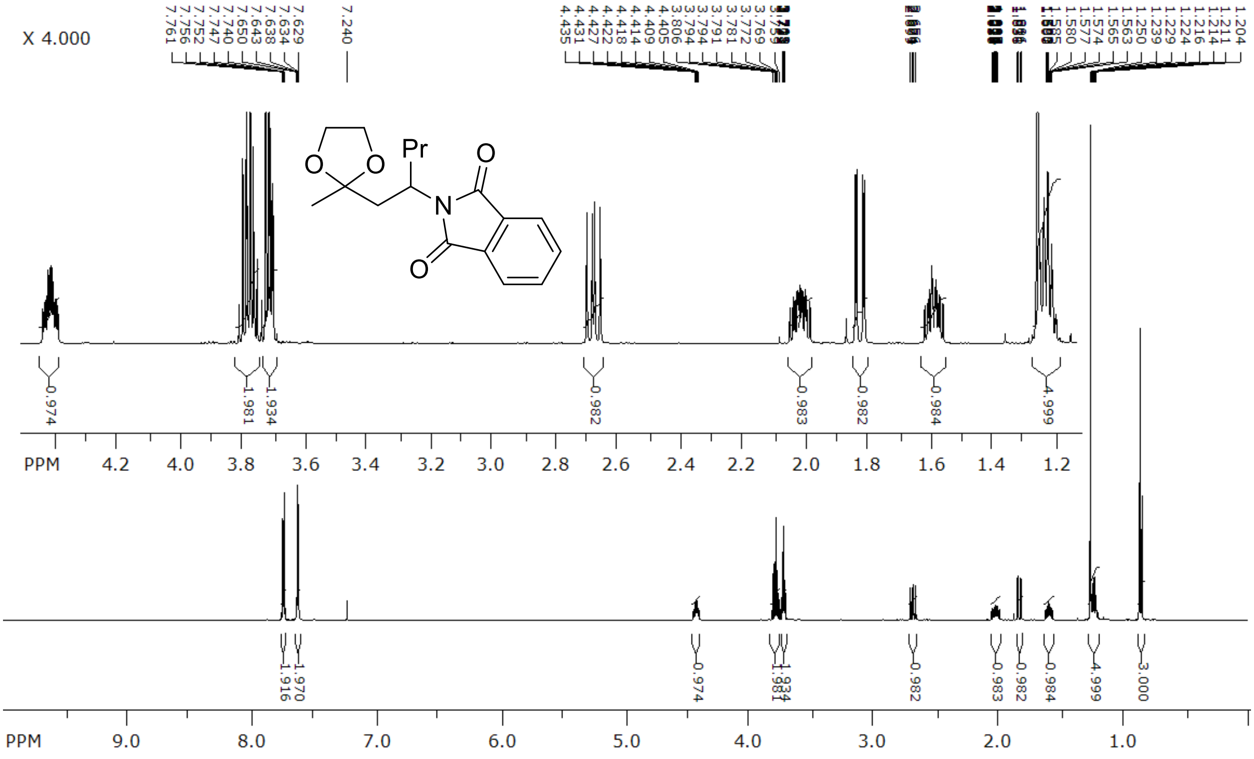

^{13}C {^{1}H} NMR (150 MHz, $CDCl_3$) of 1-(2-methyl-1,3-dioxolan-2-yl)-2-(1,3-dioxoisoindolin-2-yl)pentane (**3b**)

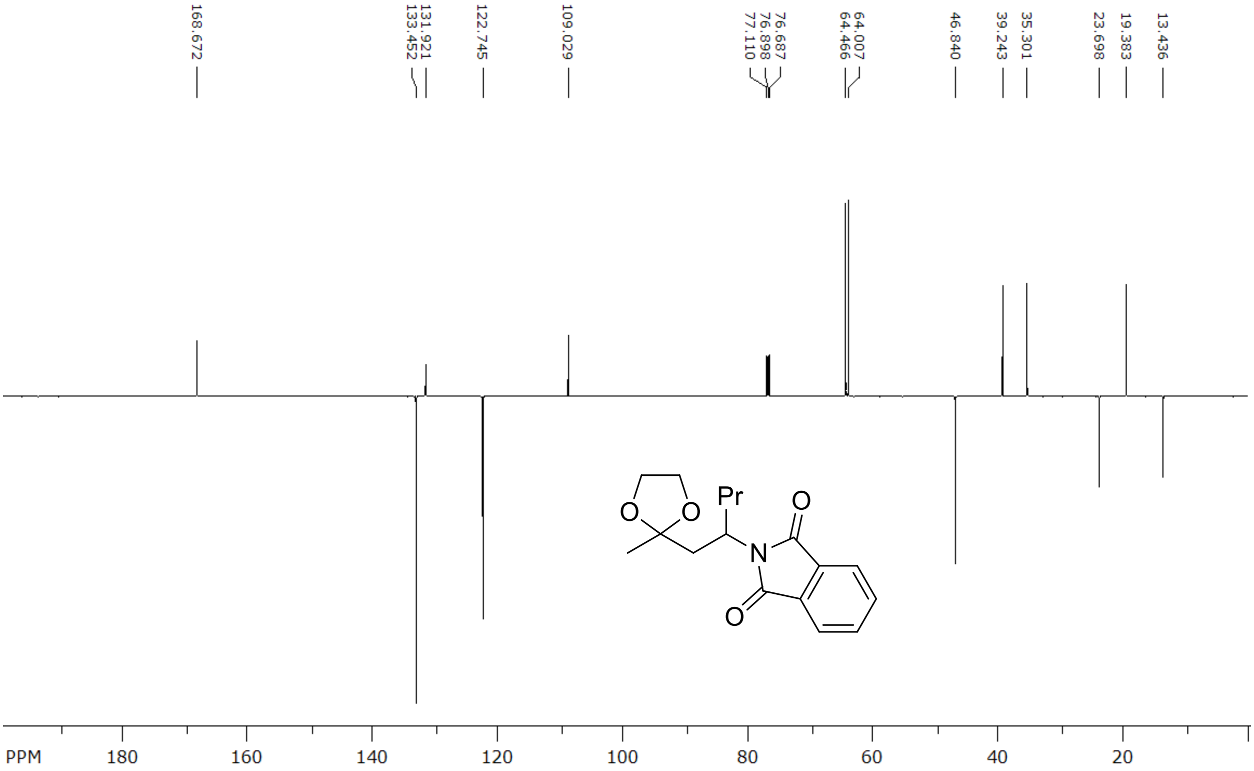

^{1}H NMR (500 MHz, $CDCl_3$) of 1-(2-methyl-1,3-dioxolan-2-yl)pentan-2-amine (**4b**)

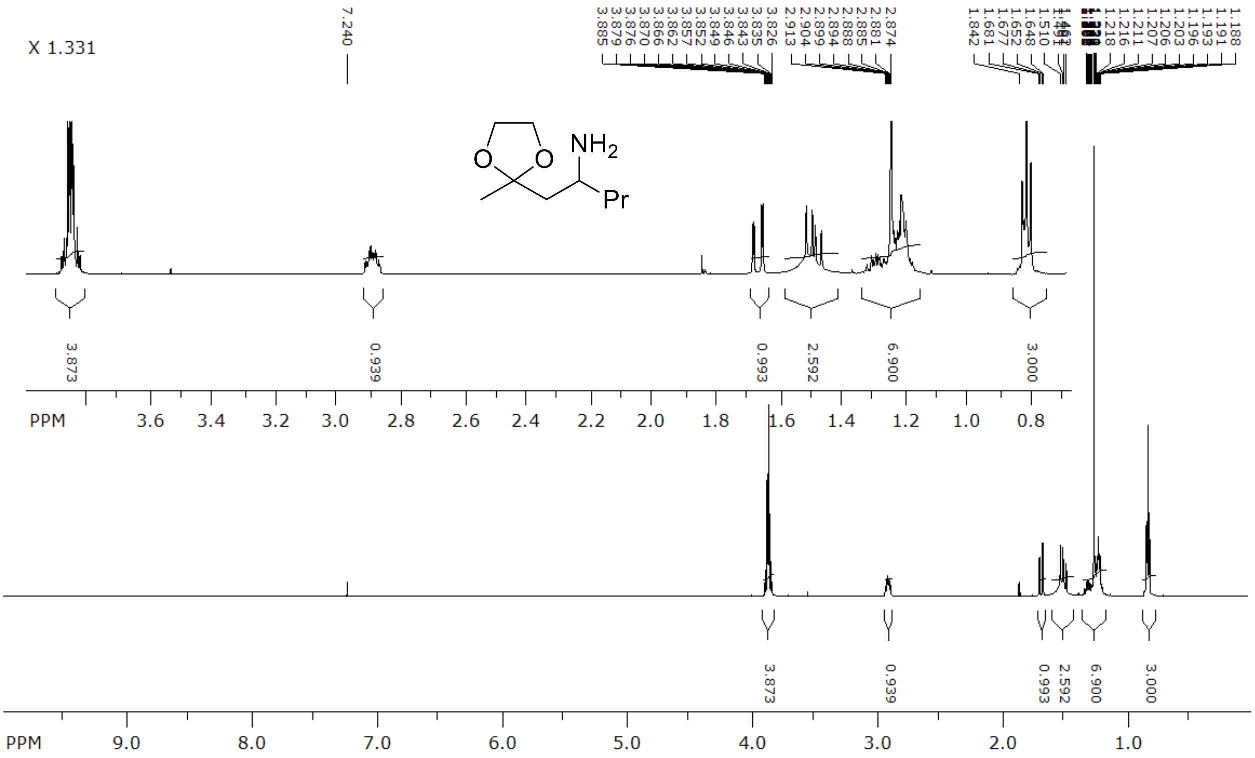

^{13}C {^{1}H} NMR (500 MHz, $CDCl_3$) of 1-(2-methyl-1,3-dioxolan-2-yl)pentan-2-amine (**4b**)

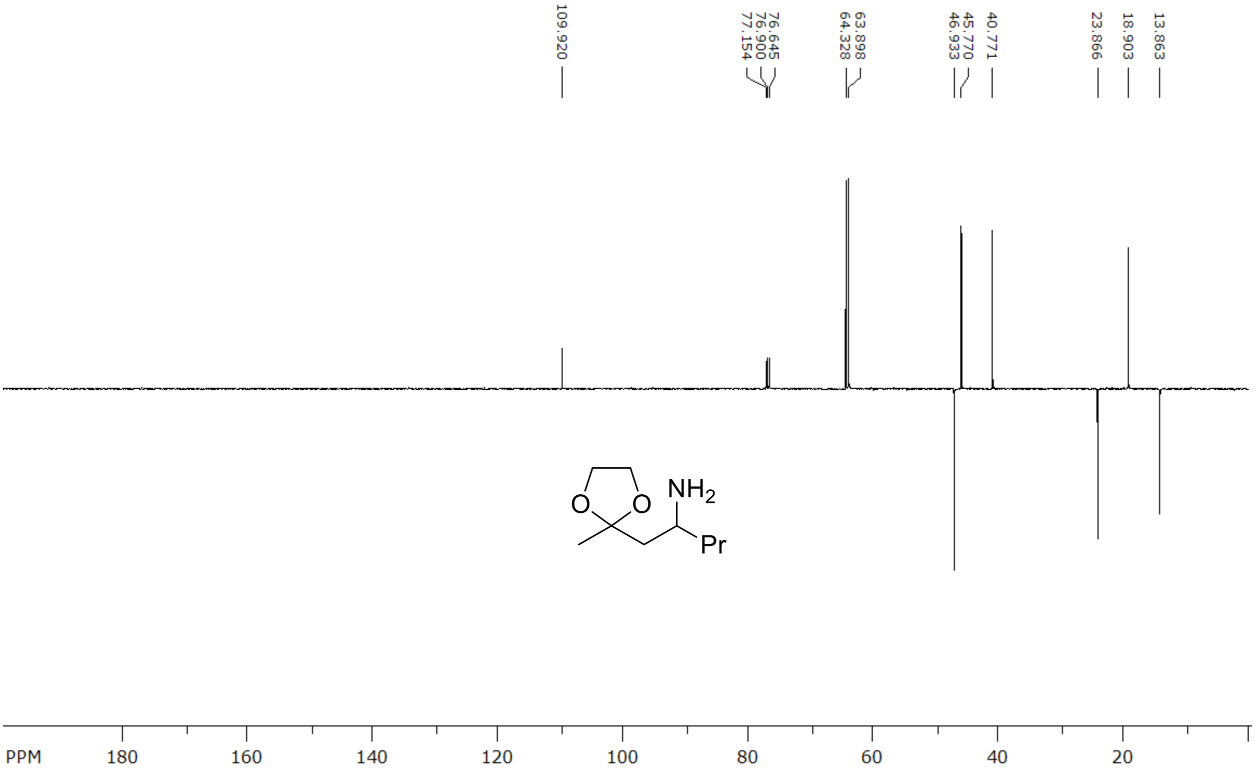

^{1}H NMR (500 MHz, $CDCl_3$) of 7,7,9-triethyl-1,4-dioxa-8-azaspiro[4.5]decane (**5a**)

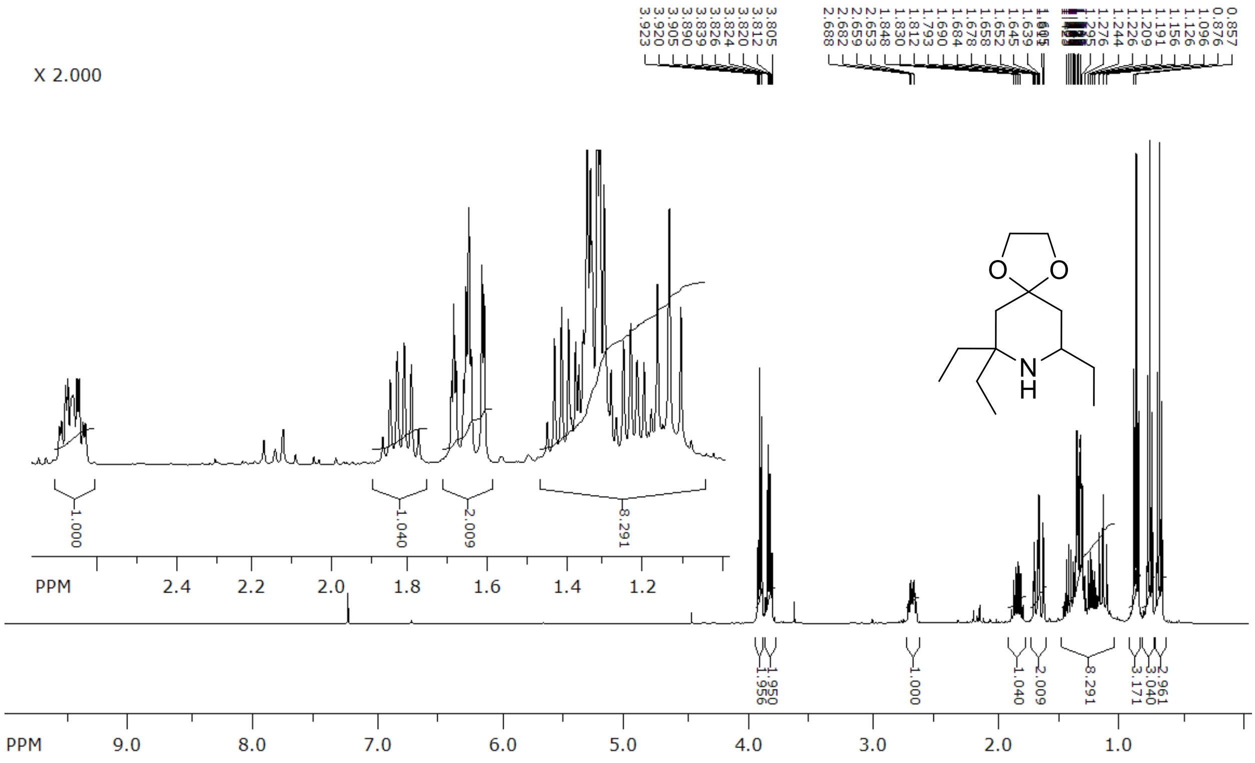

^{13}C {^{1}H} NMR (125 MHz, $CDCl_3$) of 7,7,9-triethyl-1,4-dioxa-8-azaspiro[4.5]decane (**5a**)

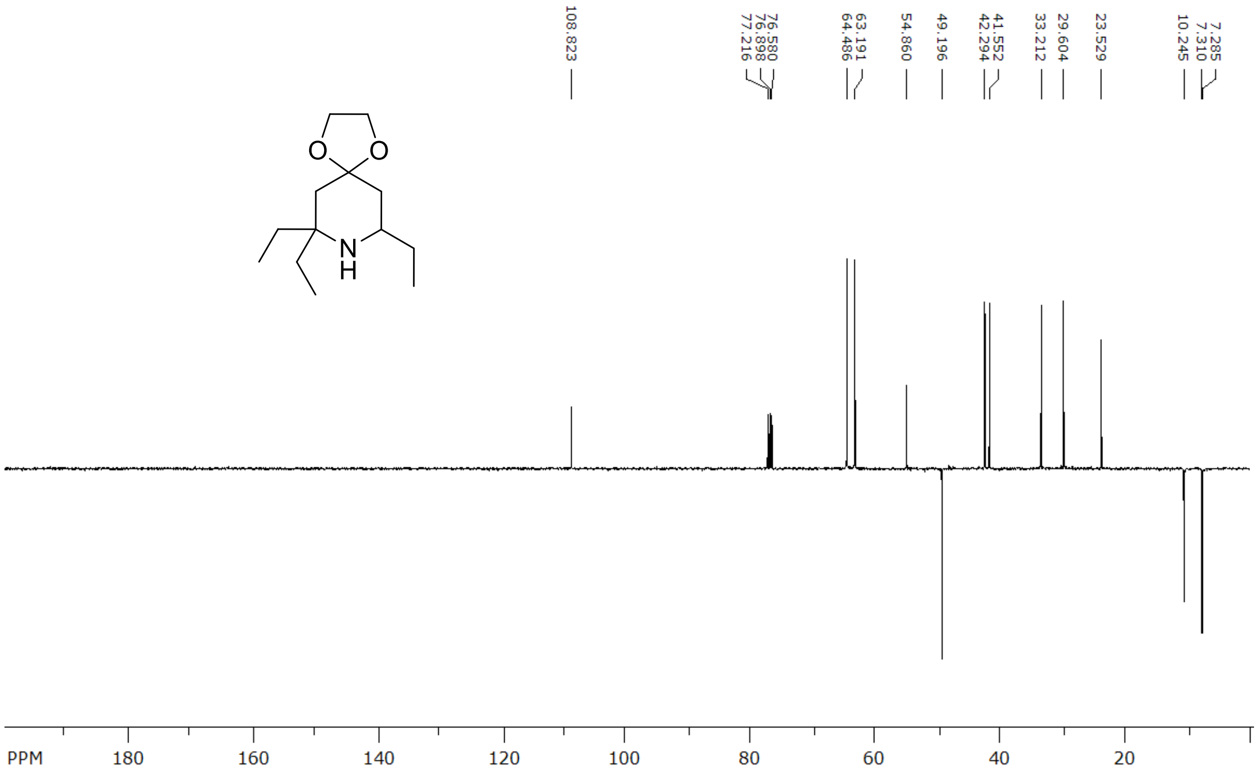

^{1}H NMR (300 MHz, $CDCl_3$) of 7,7,9-tripropyl-1,4-dioxa-8-azaspiro[4.5]decane (**5b**)

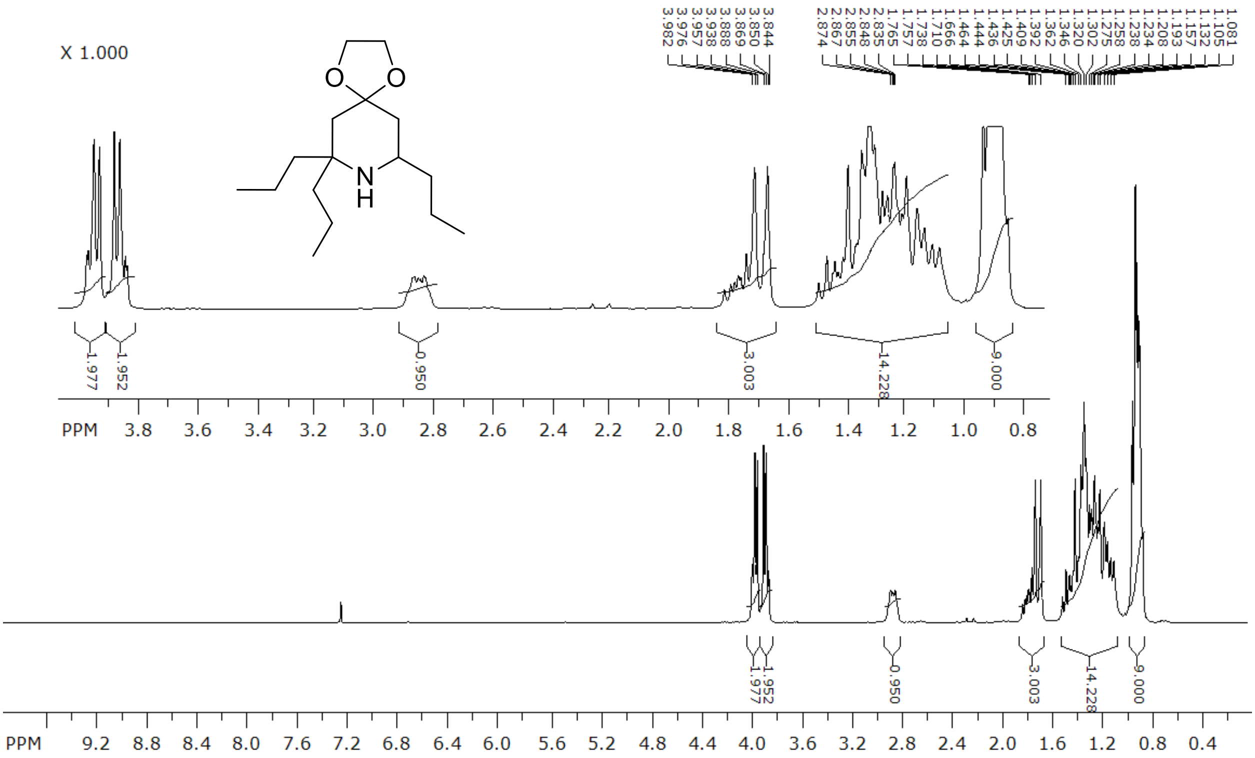

^{13}C {^{1}H} NMR (75 MHz, $CDCl_3$) of 7,7,9-tripropyl-1,4-dioxa-8-azaspiro[4.5]decane (**5b**)

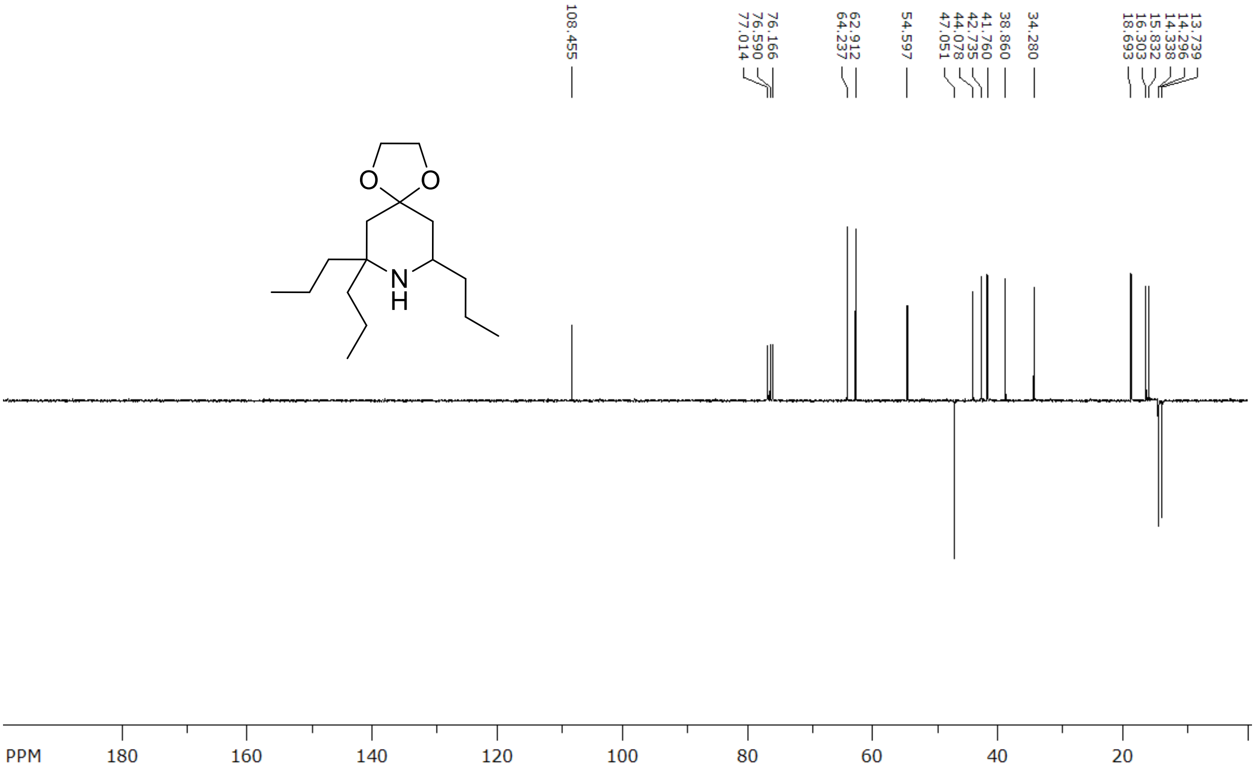

^{1}H NMR (300 MHz, CD_3OD, Zn/CF_3COOH system) of 7,7,9-triethyl-9-ethynyl-1,4-dioxa-8-azaspiro[4.5]decane-8-oxy (**7a**)

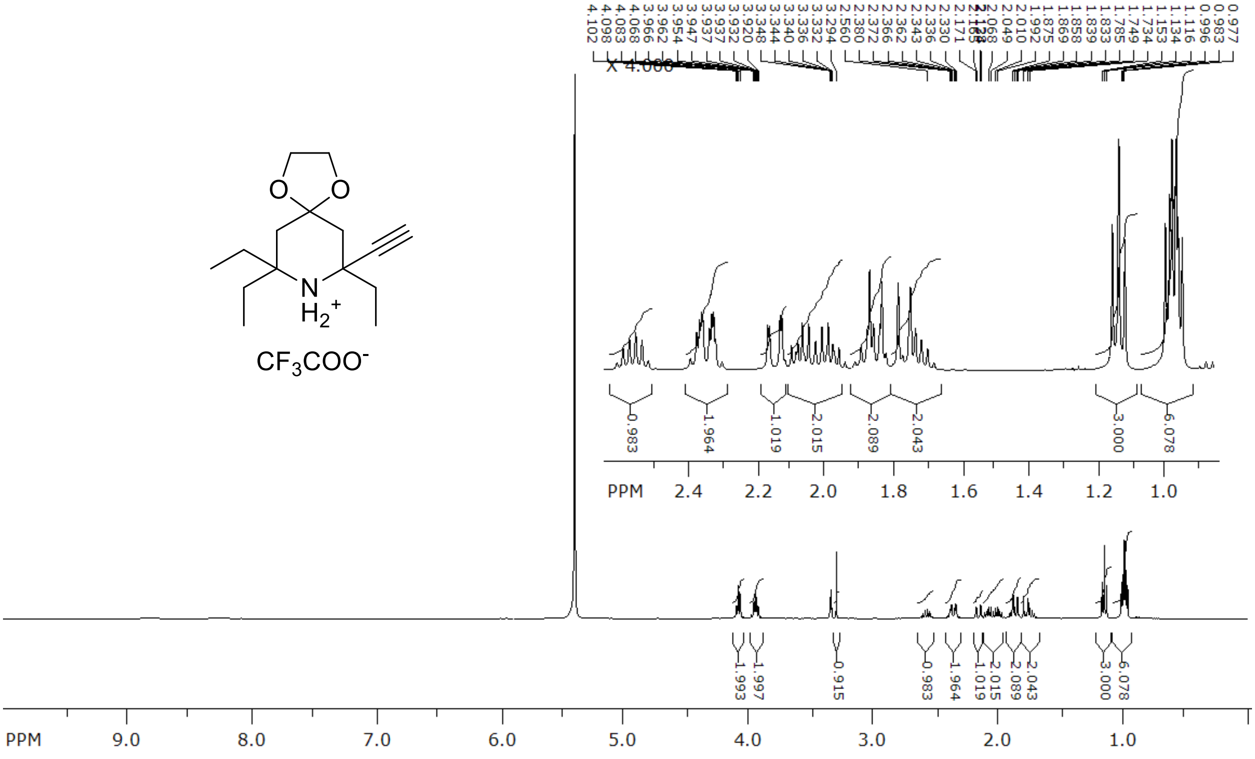

^{1}H NMR (300 MHz, CD_3OD, Zn/CF_3COOH) of 7,7,9-triethyl-9-(3-hydroxyprop-1-yn-1-yl)-1,4-dioxa-8-azaspiro[4.5]decan-8-oxyl (**7b**)

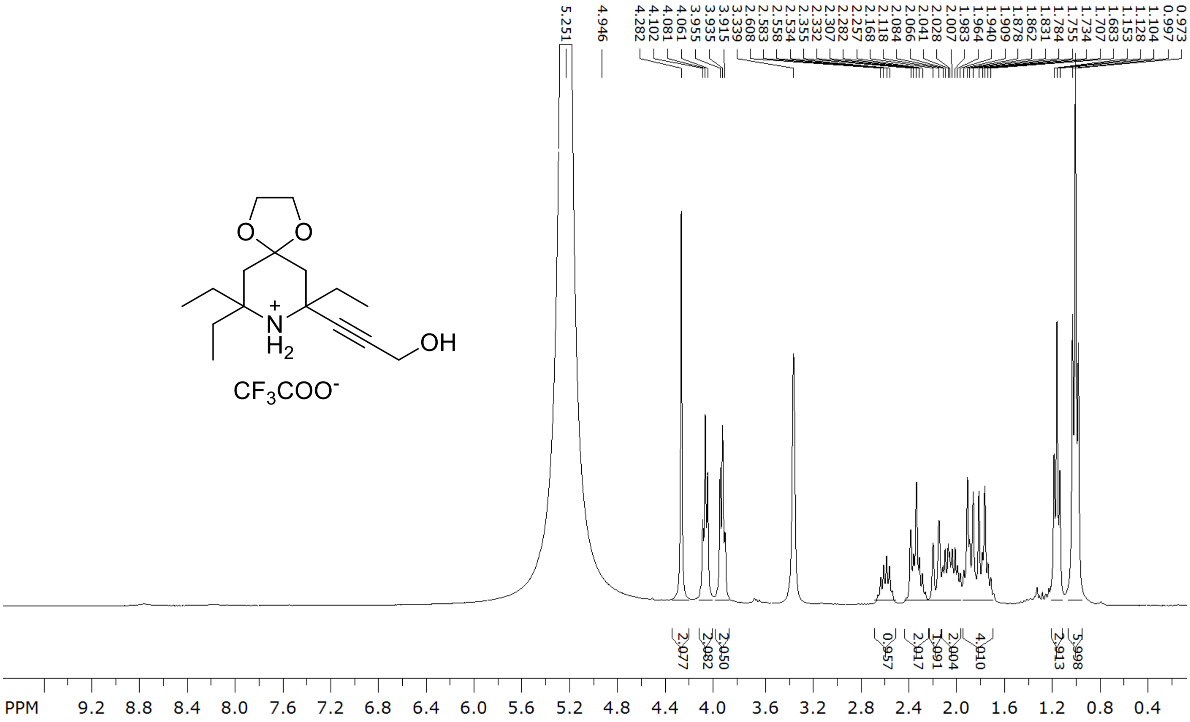

^{1}H NMR (300 MHz, CD_3OD, Zn/CF_3COOH) of 7-(3-((*tert*-butoxycarbonyl)amino)prop-1-yn-1-yl)-7,9,9-triethyl-1,4-dioxa-8-azaspiro[4.5]decan-8-oxyl (**7c**)

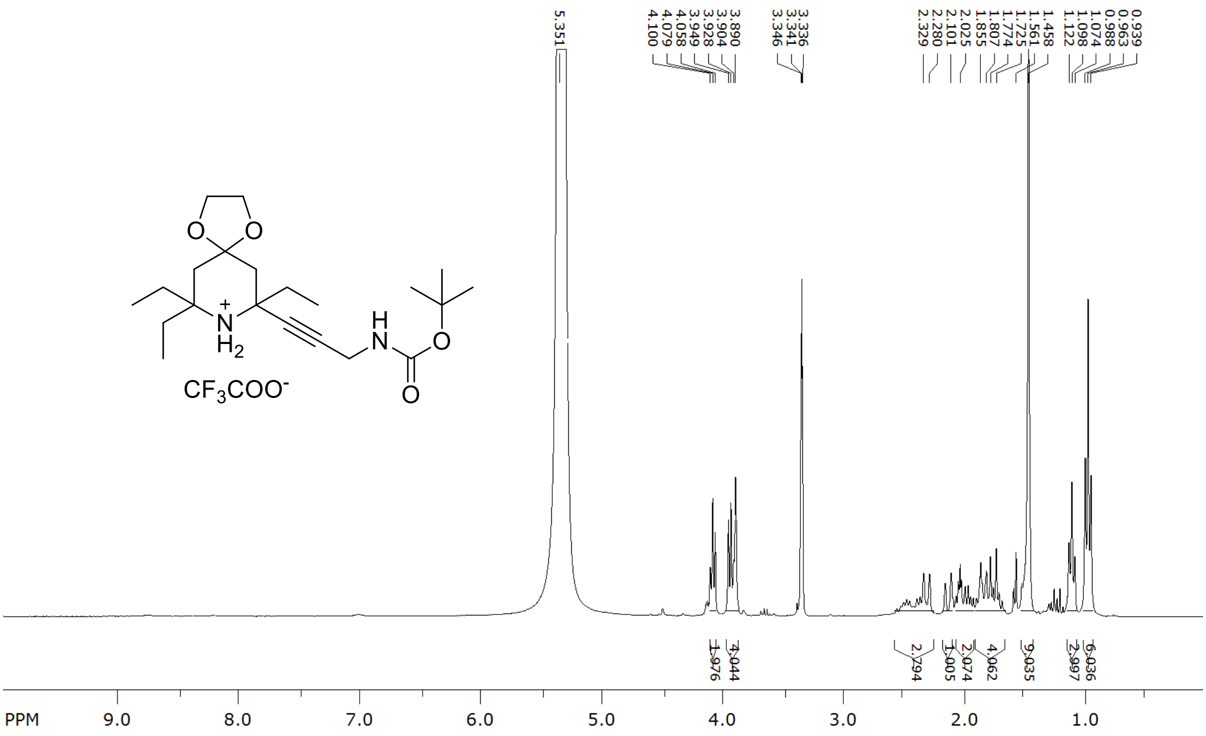

^{1}H NMR (400 MHz, CD_3OD, Zn/CF_3COOH system) of 7,7,9,9-tetraethyl-1,4-dioxa-8-azaspiro[4.5]decane-8-oxyl (**8a**)

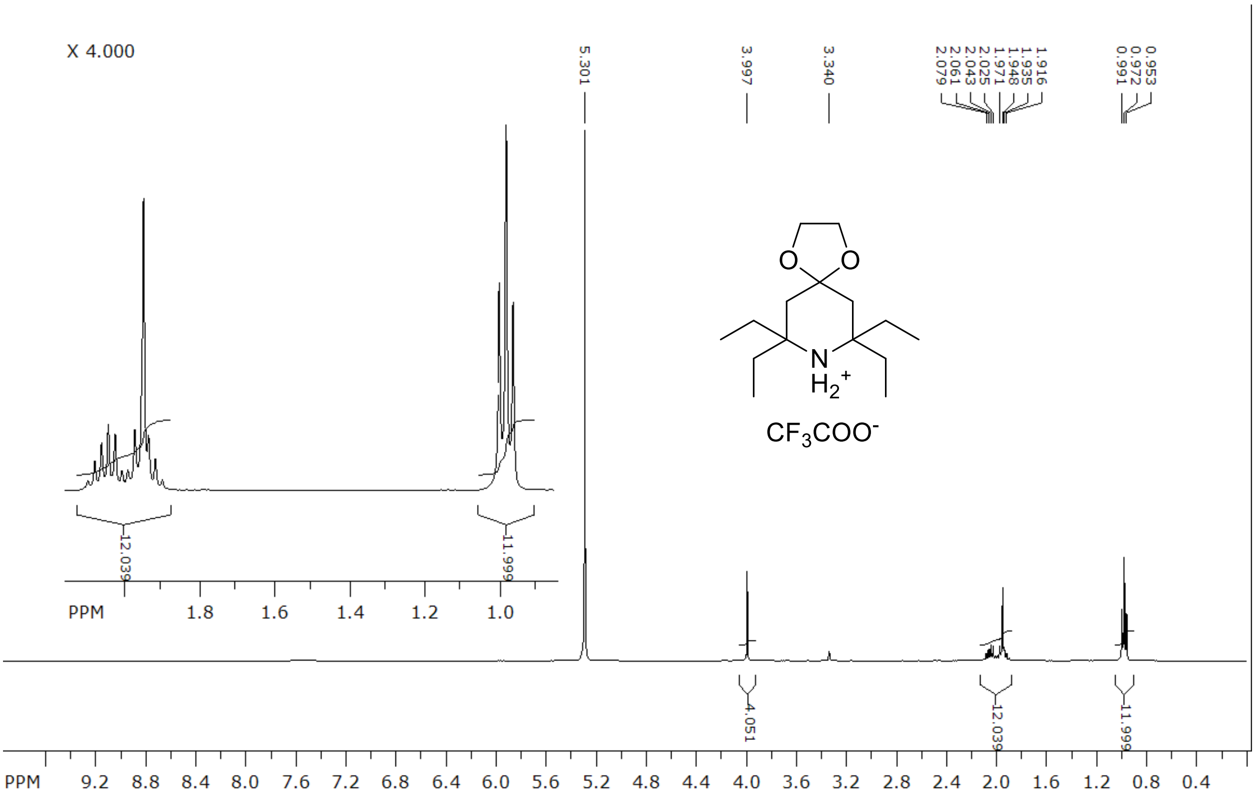

^{1}H NMR (400 MHz, CD_3OD, Zn/CF_3COOH system) of 2,2,6,6-tetraethylpiperidin-4-one-1-oxyl (**9a**)

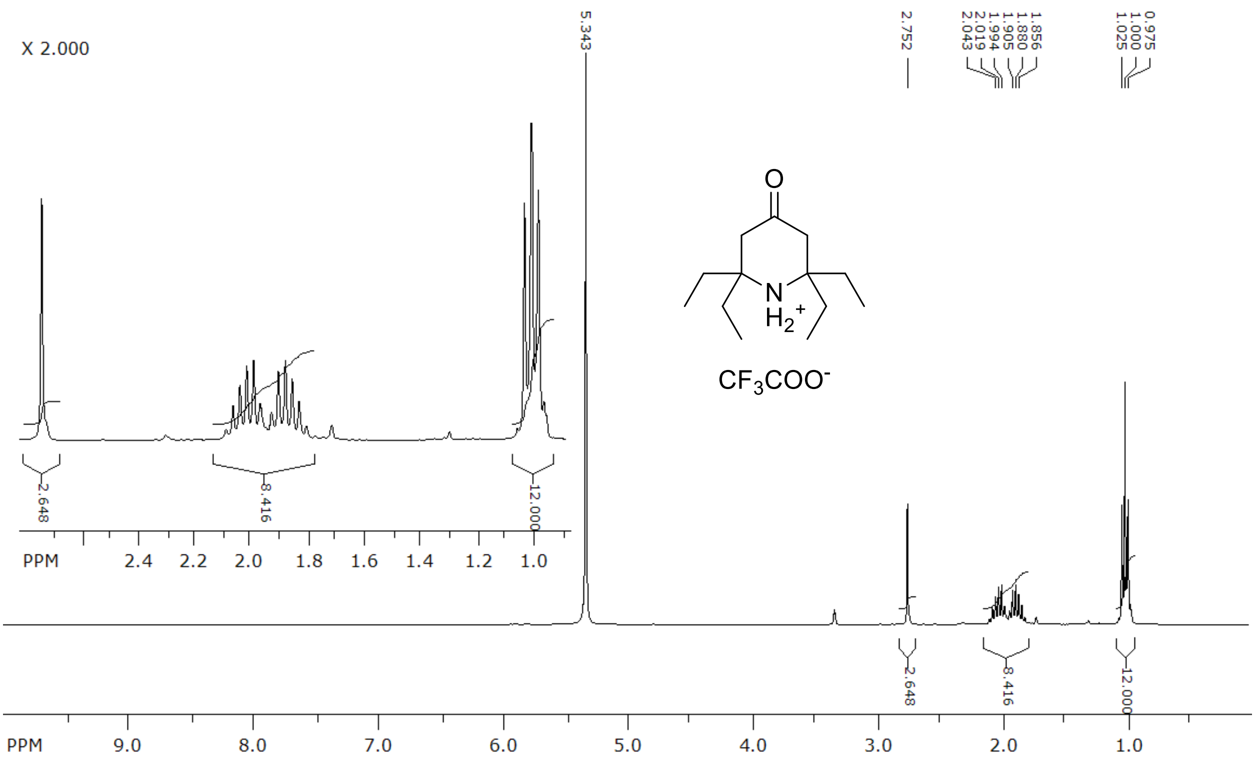

^{1}H NMR (400 MHz, CD_3OD, Zn/CF_3COOH) of 7,7,9-triethyl-9-(3-hydroxypropyl)-1,4-dioxa-8-azaspiro[4.5]decan-8-oxyl (**8b**)

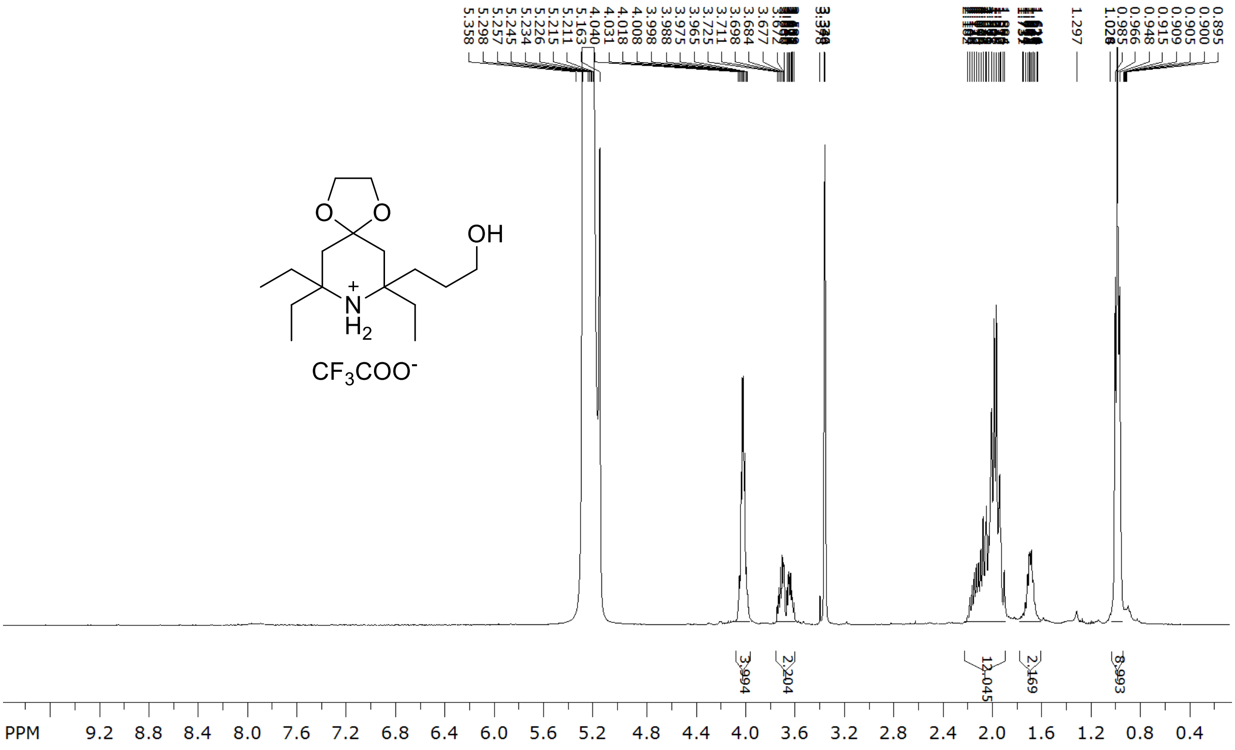

^1H NMR (300 MHz, CD_3OD, Zn/CF_3COOH) of 7-(3-((*tert*-butoxycarbonyl)amino)propyl)-7,9,9-triethyl-1,4-dioxa-8-azaspiro[4.5]decan-8-oxyl (**8c**)

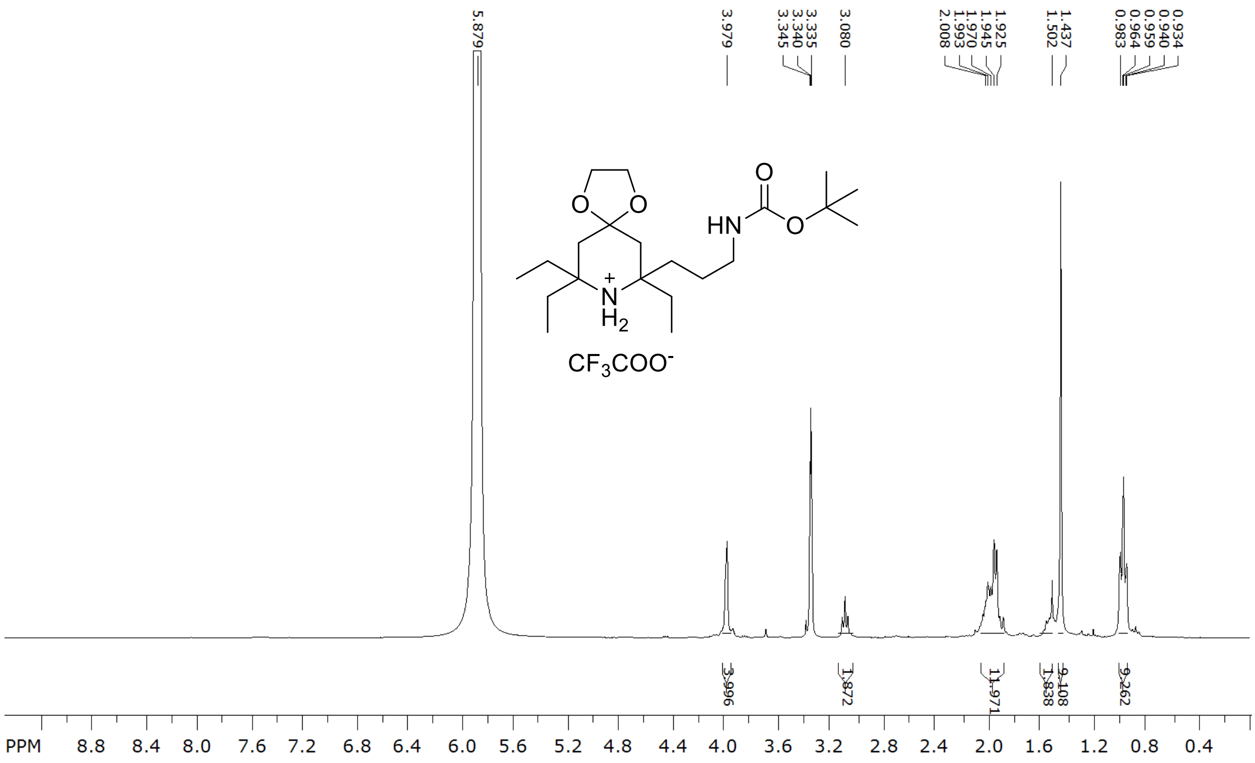

^1H NMR (300 MHz, CD_3OD, Zn/CF_3COOH system) of 7,7,9-tripropyl-9-(prop-2-en-1-yl)-1,4-dioxa-8-azaspiro[4.5]decane-8-oxyl (**7d**)

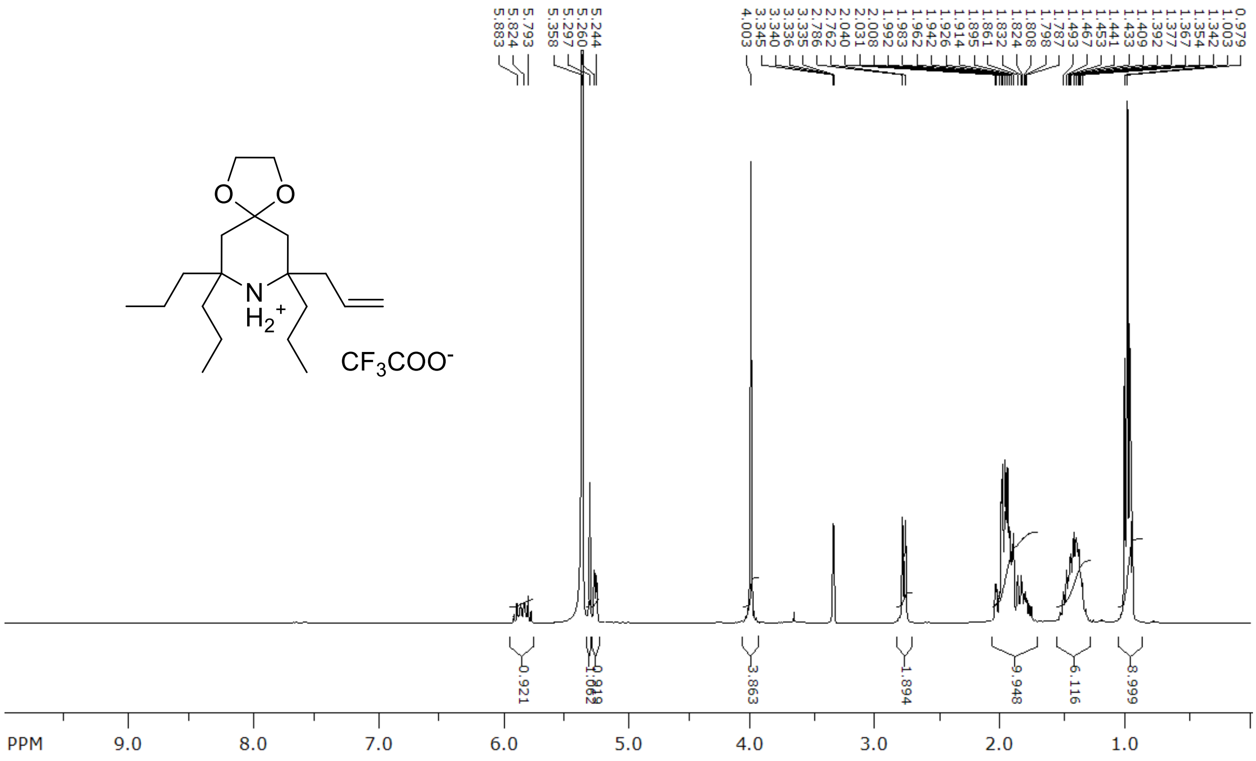

^{1}H NMR (300 MHz, CD_3OD, Zn/CF_3COOH system) of 7,7,9,9-tetrapropyl-1,4-dioxa-8-azaspiro[4.5]decane-8-oxyl (**8d**)

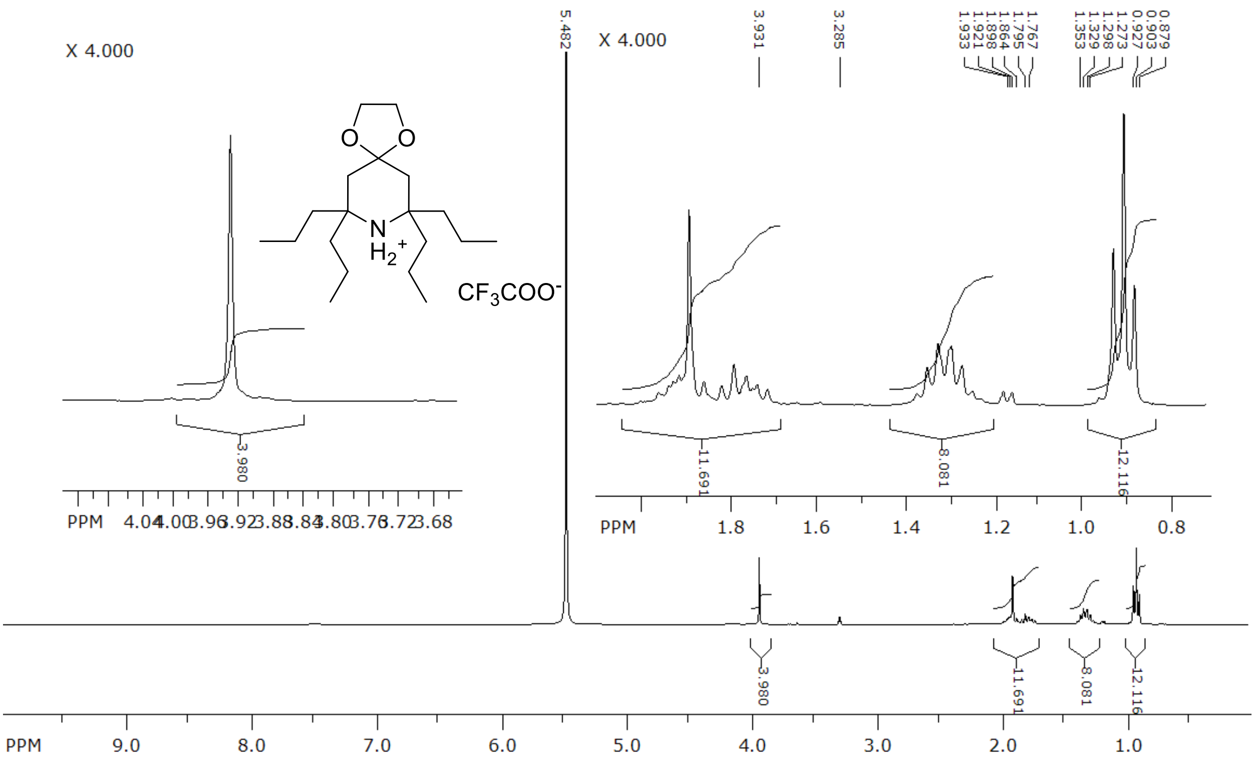

^{1}H NMR (300 MHz, CD_3OD, Zn/CF_3COOH system) of 2,2,6,6-tetrapropylpiperidin-4-one-1-oxyl (**9b**)

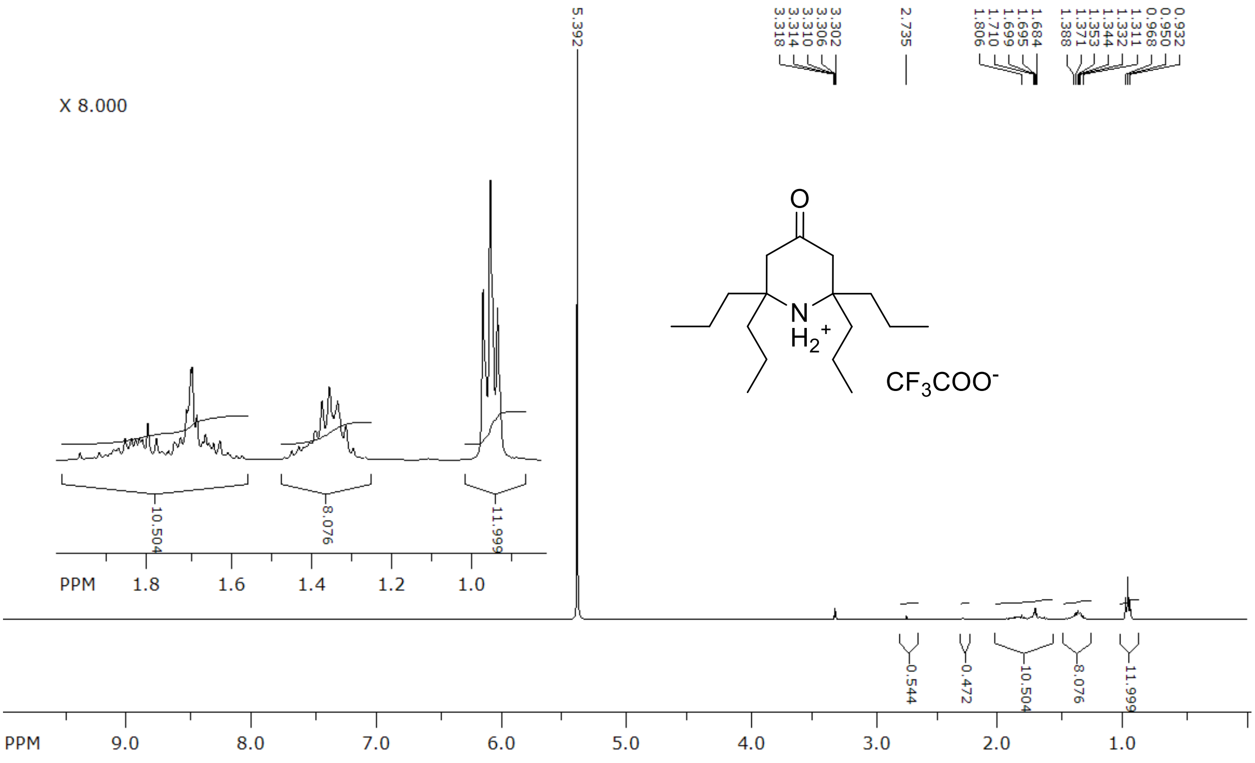

Line shape analysis of NMR multiplets

Line shape analysis of multiplets for 7,7,9-triethyl-1,4-dioxa-8-azaspiro[4.5]decane (**5a**)

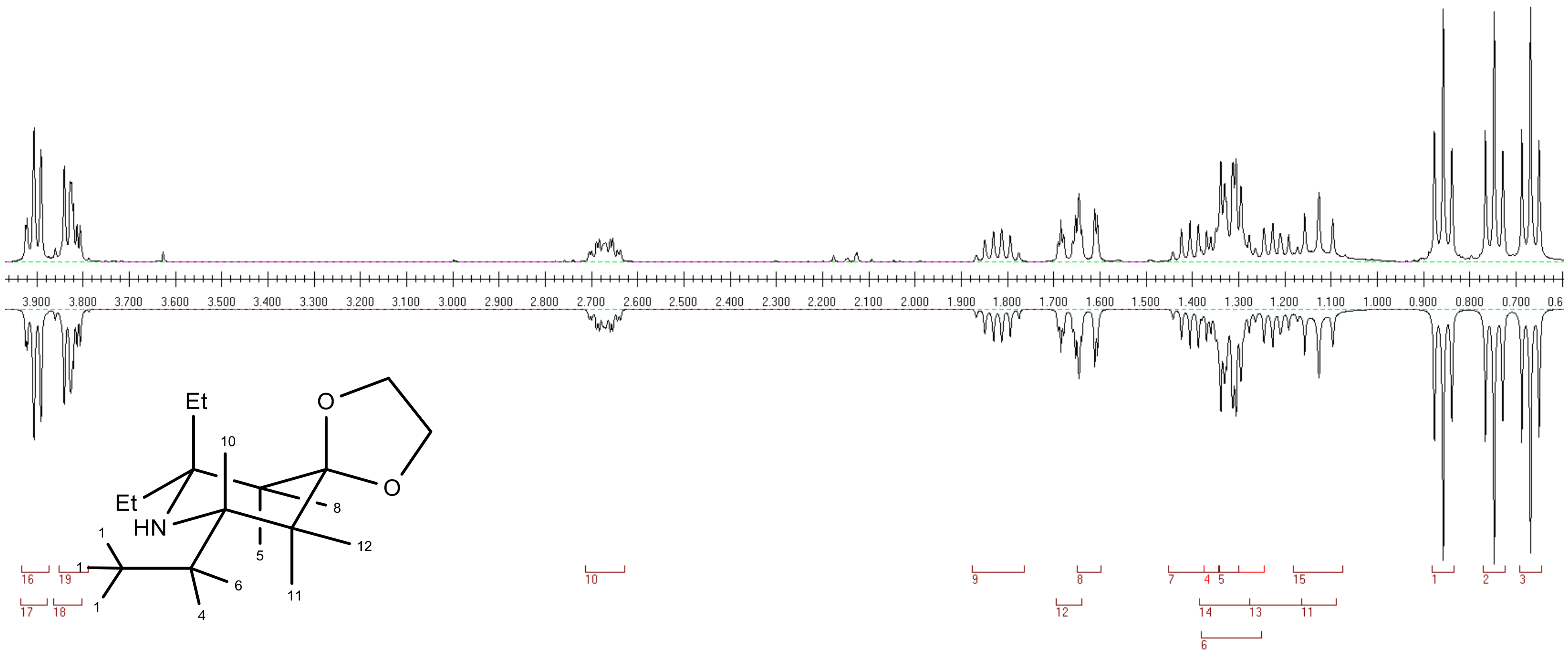

Table S1. Spin system parameters for 7,7,9-triethyl-1,4-dioxa-8-azaspiro[4.5]decane (**5a**)

Numb	Nucleus	n	Shift	J[1]	J[2]	J[]	J[4]	J[5]	J[6]	J[7]	J[8]	J[9]	J[10]	J[11]	J[12]	J[13]	J[14]	J[15]	J[16]	J[17]	J[18]
1	^{1}H	3	0.858*																		
2	^{1}H	3	0.747*	-																	
3	^{1}H	3	0.669*	-	-																
4	^{1}H	1	1.309	7.50	-	-															
5	^{1}H	1	1.323	-	-	-	-														
6	^{1}H	1	1.315	7.46	-	-	-14.29	-													
7	^{1}H	1	1.396	-	-	7.35	-	-	-												
8	^{1}H	1	1.624	-	-	-	-	-13.55	-	-											
9	^{1}H	1	1.820	-	-	7.57	-	-	-	-14.37	-										
10	^{1}H	1	2.671	-	-	-	6.34	-	6.63	-	-	-									
11	^{1}H	1	1.127	-	-	-	-	-	-	-	-	-	11.76								
12	^{1}H	1	1.668	-	-	-	-	-	-	-	-2.40	-	2.48	-12.68							
13	^{1}H	1	1.220	-	7.57	-	-	-	-	-	-	-	-	-	-						
14	^{1}H	1	1.331	-	7.45	-	-	-	-	-	-	-	-	-	-	-13.83					
15	^{1}H	1	1.129	-	-	-	-	-	-	-	-	-	-	-	-	-	-				
16	^{1}H	1	3.903	-	-	-	-	-	-	-	-	-	-	-	-	-	-	-			
17	^{1}H	1	3.906	-	-	-	-	-	-	-	-	-	-	-	-	-	-	-	-6.20		
18	^{1}H	1	3.833	-	-	-	-	-	-	-	-	-	-	-	-	-	-	-	6.42	6.15	
19	^{1}H	1	3.819	-	-	-	-	-	-	-	-	-	-	-	-	-	-	-	6.21	6.57	-7.66

*Defined up to a permutation

Line shape analysis of multiplets for 7,7,9-triethyl-9-ethynyl-1,4-dioxa-8-azaspiro[4.5]decane-8-oxy (**7a**)

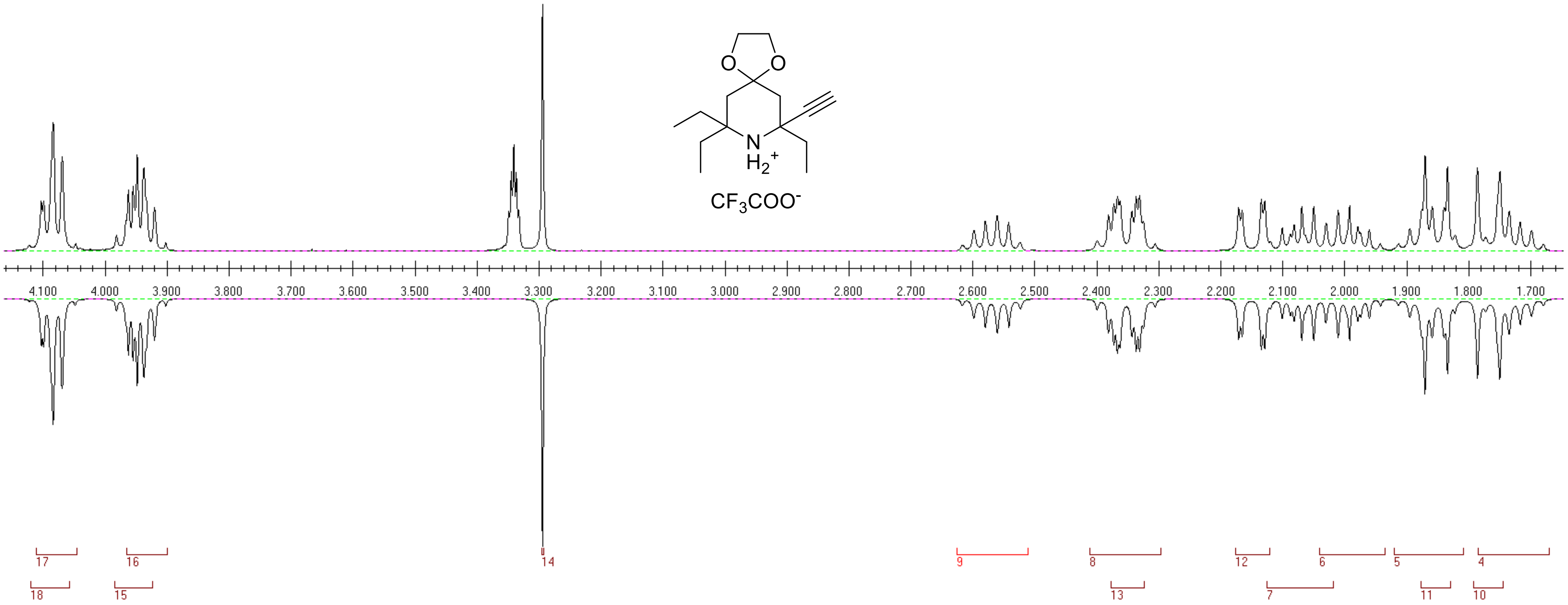

Table S2. Spin system parameters for 7,7,9-triethyl-9-ethynyl-1,4-dioxa-8-azaspiro[4.5]decane-8-oxy (**7a**)

Numb	Nucleus	n	Shift	J[1]	J[2]	J[3]	J[4]	J[5]	J[6]	J[7]	J[8]	J[9]	J[10]	J[11]	J[12]	J[13]	J[14]	J[15]	J[16]	J[17]
4	^{1}H	1,00	1,73	7,61	-	-														
5	^{1}H	1,00	1,87	7,40	-	-	-14,42													
6	^{1}H	1,00	1,99	-	7,40	-	-	-												
7	^{1}H	1,00	2,07	-	7,56	-	-	-	-12,70											
8	^{1}H	1,00	2,35	-	-	7,41	-	-	-	-										
9	^{1}H	1,00	2,57	-	-	7,26	-	-	-	-	-15,28									
10	^{1}H	1,00	1,77	-	-	-	-	-	-	-	-	-								
11	^{1}H	1,00	1,85	-	-	-	-	-	-	-	-	-	-							
12	^{1}H	1,00	2,15	-	-	-	-	-	-	-	-	-	-	-14,66						
13	^{1}H	1,00	2,35	-	-	-	-	-	-	-	-	-	-14,48	-	2,27					
14	^{1}H	1,00	3,29	-	-	-	-	-	-	-	-	-	-	-	-	-				
15	^{1}H	1,00	3,95	-	-	-	-	-	-	-	-	-	-	-	-	-	-			
16	^{1}H	1,00	3,93	-	-	-	-	-	-	-	-	-	-	-	-	-	-	-7,68		
17	^{1}H	1,00	4,08	-	-	-	-	-	-	-	-	-	-	-	-	-	-	6,80	6,51	
18	^{1}H	1,00	4,09	-	-	-	-	-	-	-	-	-	-	-	-	-	-	5,53	6,74	-7,94

Line shape analysis of multiplets for 7,7,9-triethyl-9-(3-hydroxyprop-1-yn-1-yl)-1,4-dioxa-8-azaspiro[4.5]decan-8-oxyl (**7b**)

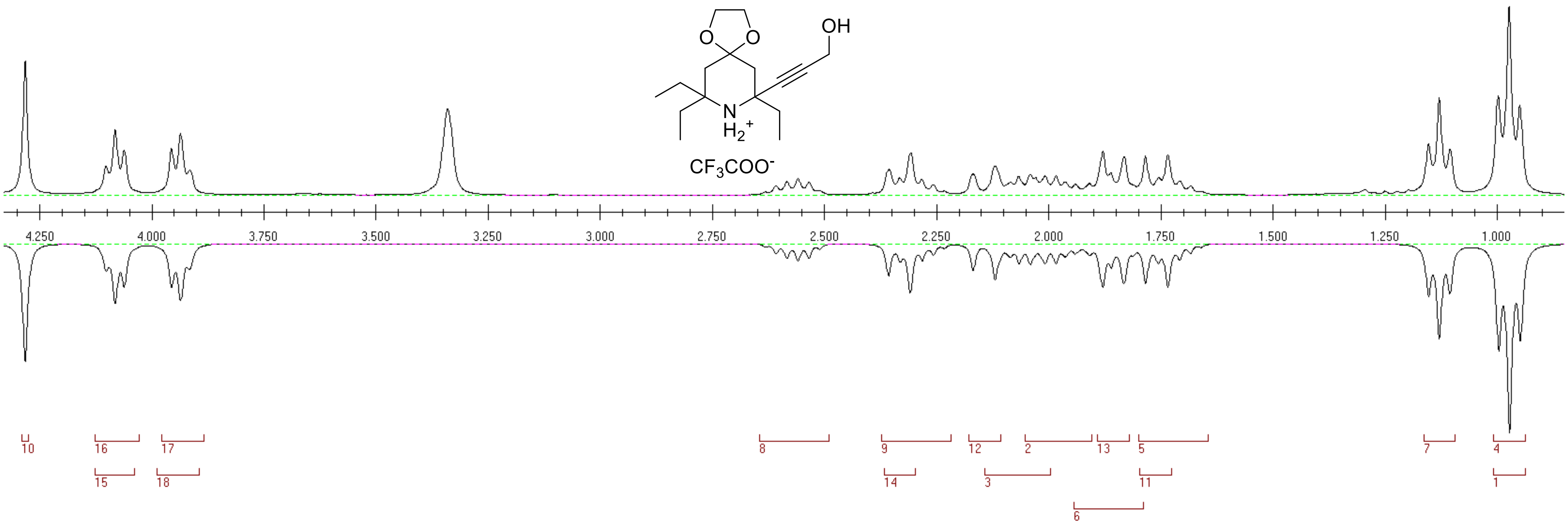

Table S3. Spin system parameters for 7,7,9-triethyl-9-(3-hydroxyprop-1-yn-1-yl)-1,4-dioxa-8-azaspiro[4.5]decan-8-oxyl (**7b**)

Numb	Nucleus	n	Shift	J[1]	J[2]	J[3]	J[4]	J[5]	J[6]	J[7]	J[8]	J[9]	J[10]	J[11]	J[12]	J[13]	J[14]	J[15]	J[16]	J[17]
2	^{1}H	1,00	1,98	7,30	-	-	-	-	-	-	-	-	-	-	-	-	-	-	-	-
3	^{1}H	1,00	2,07	7,30	-12,70	-	-	-	-	-	-	-	-	-	-	-	-	-	-	-
5	^{1}H	1,00	1,72	-	-	-	7,35	-	-	-	-	-	-	-	-	-	-	-	-	-
6	^{1}H	1,00	1,87	-	-	-	7,35	-14,40	-	-	-	-	-	-	-	-	-	-	-	-
8	^{1}H	1,00	2,57	-	-	-	-	-	-	7,30	-	-	-	-	-	-	-	-	-	-
9	^{1}H	1,00	2,30	-	-	-	-	-	-	7,30	-14,90	-	-	-	-	-	-	-	-	-
10	^{1}H	2,00	4,28	-	-	-	-	-	-	-	-	-	-	-	-	-	-	-	-	-
11	^{1}H	1,00	1,76	-	-	-	-	-	-	-	-	-	-	-	-	-	-	-	-	-
12	^{1}H	1,00	2,14	-	-	-	-	-	-	-	-	-	-	-14,70	-	-	-	-	-	-
13	^{1}H	1,00	1,86	-	-	-	-	-	-	-	-	-	-	-	-	-	-	-	-	-
14	^{1}H	1,00	2,33	-	-	-	-	-	-	-	-	-	-	-	-	-14,35	-	-	-	-
15	^{1}H	1,00	4,08	-	-	-	-	-	-	-	-	-	-	-	-	-	-	-	-	-
16	^{1}H	1,00	4,08	-	-	-	-	-	-	-	-	-	-	-	-	-	-	-7,70	-	-
17	^{1}H	1,00	3,93	-	-	-	-	-	-	-	-	-	-	-	-	-	-	5,40	7,05	-
18	^{1}H	1,00	3,94	-	-	-	-	-	-	-	-	-	-	-	-	-	-	5,55	7,00	-8,05

Line shape analysis of multiplets for 7-(3-((*tert*-butoxycarbonyl)amino)prop-1-yn-1-yl)-7,9,9-triethyl-1,4-dioxa-8-azaspiro[4.5]decan-8-oxyl (**7c**)

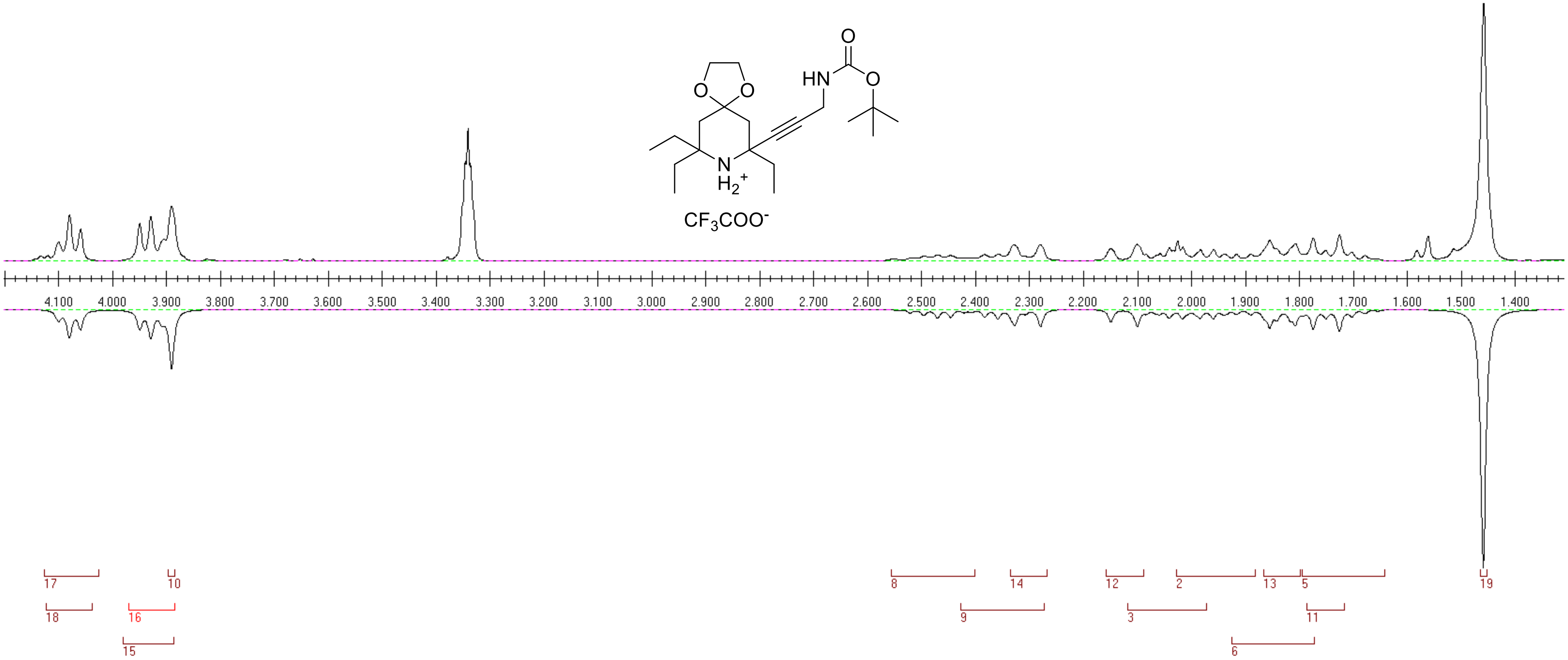

Table S4. Spin system parameters for 7-(3-((*tert*-butoxycarbonyl)amino)prop-1-yn-1-yl)-7,9,9-triethyl-1,4-dioxa-8-azaspiro[4.5]decan-8-oxyl (**7c**)

Numb	Nucleus	n	Shift	J[1]	J[2]	J[3]	J[4]	J[5]	J[6]	J[7]	J[8]	J[9]	J[10]	J[11]	J[12]	J[13]	J[14]	J[15]	J[16]	J[17]	J[18]
2	^{1}H	1,00	1,96	7,35	-	-	-	-	-	-	-	-	-	-	-	-	-	-	-	-	-
3	^{1}H	1,00	2,05	7,35	-12,65	-	-	-	-	-	-	-	-	-	-	-	-	-	-	-	-
5	^{1}H	1,00	1,72	-	-	-	7,35	-	-	-	-	-	-	-	-	-	-	-	-	-	-
6	^{1}H	1,00	1,85	-	-	-	7,35	-14,4	-	-	-	-	-	-	-	-	-	-	-	-	-
8	^{1}H	1,00	2,48	-	-	-	-	-	-	7,30	-	-	-	-	-	-	-	-	-	-	-
9	^{1}H	1,00	2,35	-	-	-	-	-	-	7,30	-15,30	-	-	-	-	-	-	-	-	-	-
10	^{1}H	2,00	3,89	-	-	-	-	-	-	-	-	-	-	-	-	-	-	-	-	-	-
11	^{1}H	1,00	1,75	-	-	-	-	-	-	-	-	-	-	-	-	-	-	-	-	-	-
12	^{1}H	1,00	2,12	-	-	-	-	-	-	-	-	-	-	-14,65	-	-	-	-	-	-	-
13	^{1}H	1,00	1,83	-	-	-	-	-	-	-	-	-	-	-	-	-	-	-	-	-	-
14	^{1}H	1,00	2,30	-	-	-	-	-	-	-	-	-	-	-	-	-14,25	-	-	-	-	-
15	^{1}H	1,00	3,93	-	-	-	-	-	-	-	-	-	-	-	-	-	-	-	-	-	-
16	^{1}H	1,00	3.93	-	-	-	-	-	-	-	-	-	-	-	-	-	-	-7.47	-	-	-
17	^{1}H	1,00	4.07															7.80	6.45	-	-
18	^{1}H	1,00	4.08															6.06	4.64	-8.55	-
19	^{1}H	9,00	1,46	-	-	-	-	-	-	-	-	-	-	-	-	-	-	-	-	-	-

IR spectra

IR (neat) of 3-(1,3-dioxoisoindolin-2-yl)-hexanone-5 (**2a**)

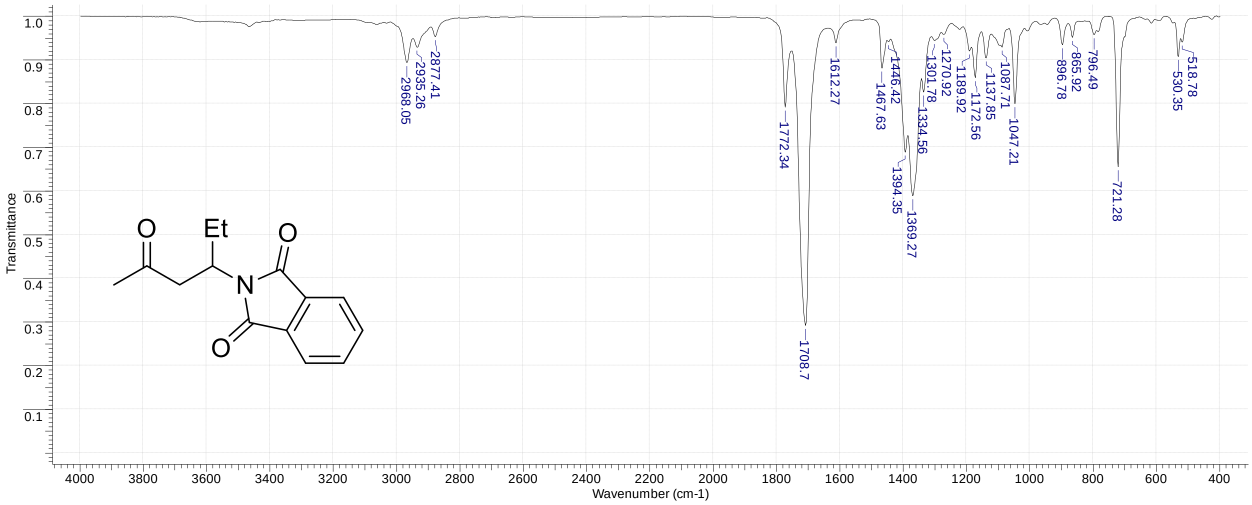

IR (neat) of 1-(2-methyl-1,3-dioxolan-2-yl)-2-(1,3-dioxoisoindolin-2-yl)butane (**3a**)

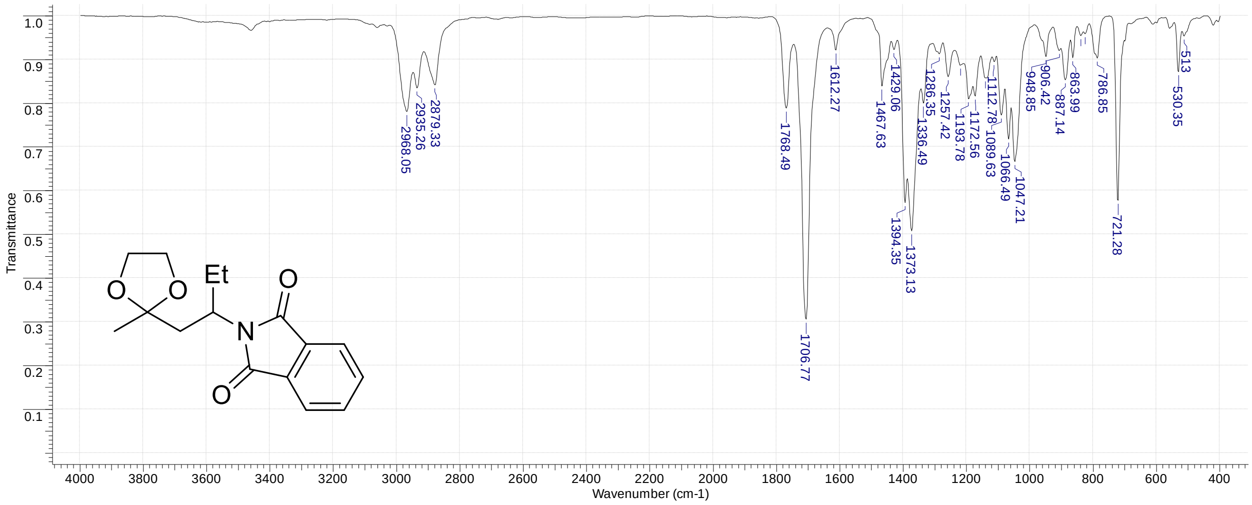

IR (neat) of 1-(2-methyl-1,3-dioxolan-2-yl)butan-2-amine (**4a**)

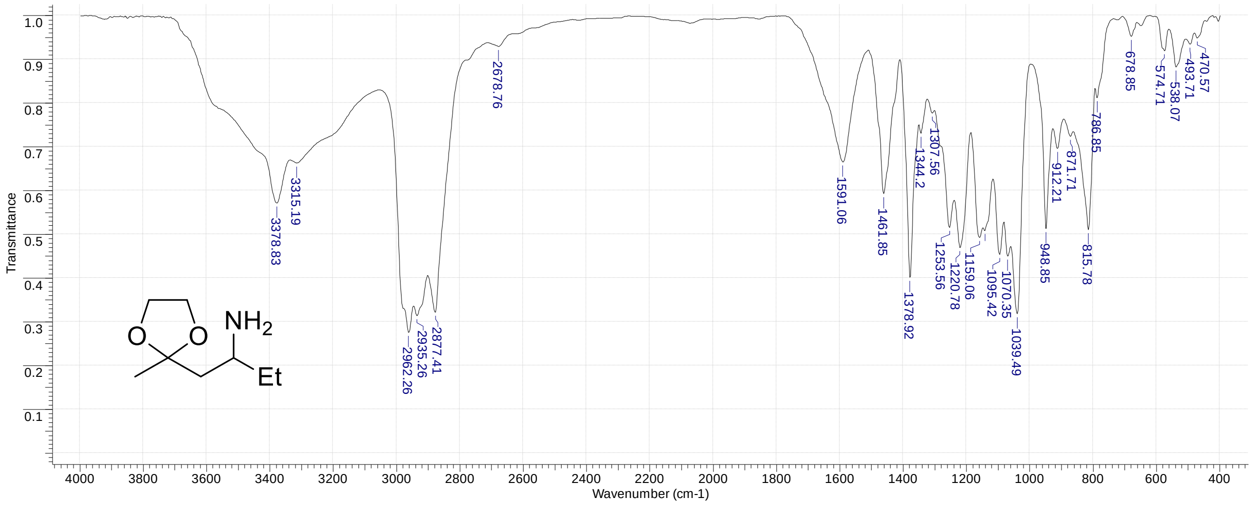

IR (neat) of 4-(1,3-dioxoisoindolin-2-yl)-heptanone-6 (**2b**).

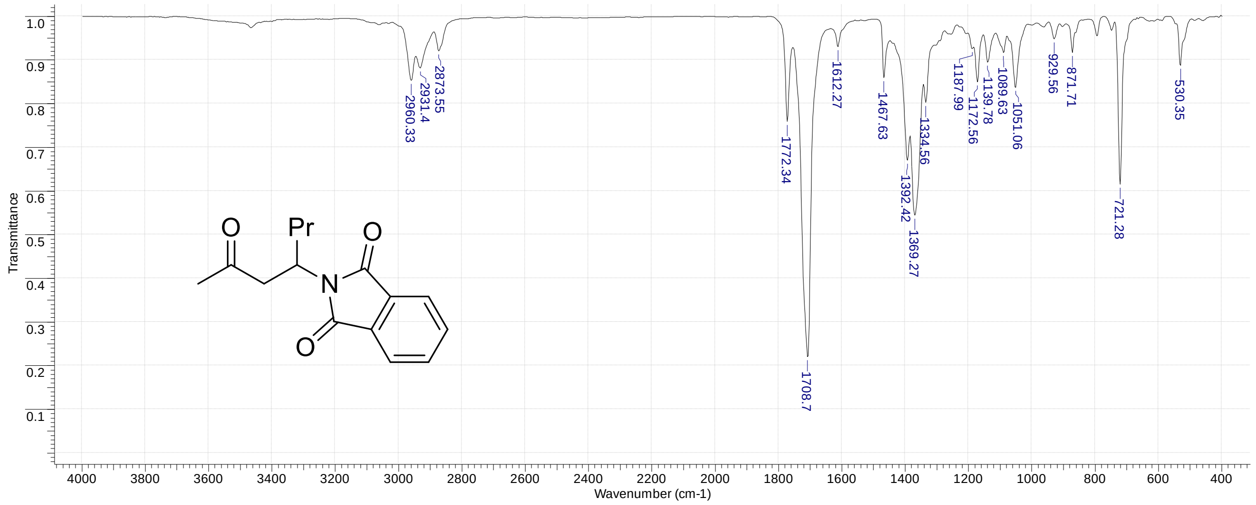

IR (neat) of 1-(2-methyl-1,3-dioxolan-2-yl)-2-(1,3-dioxoisoindolin-2-yl)pentane (**3b**)

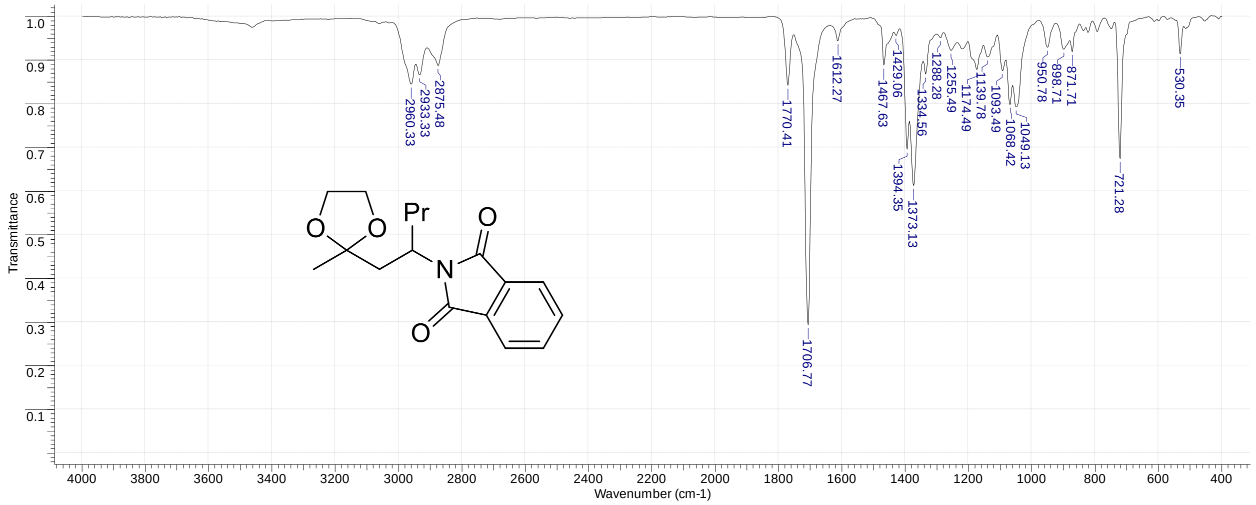

IR (neat) of 1-(2-methyl-1,3-dioxolan-2-yl)pentan-2-amine (**4b**)

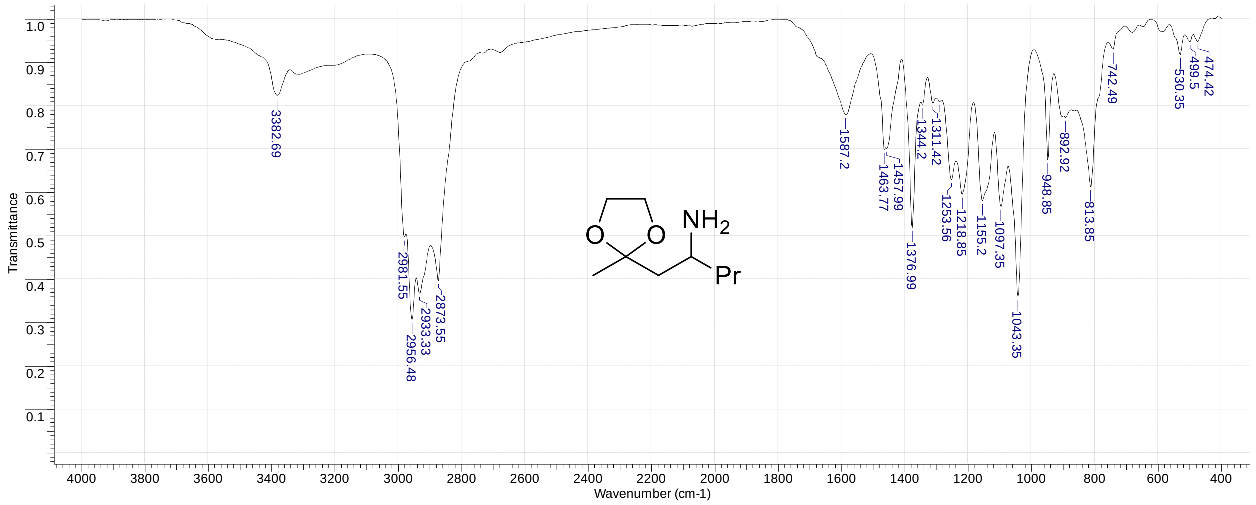

IR (neat) of 7,7,9-triethyl-1,4-dioxa-8-azaspiro[4.5]decane (**5a**)

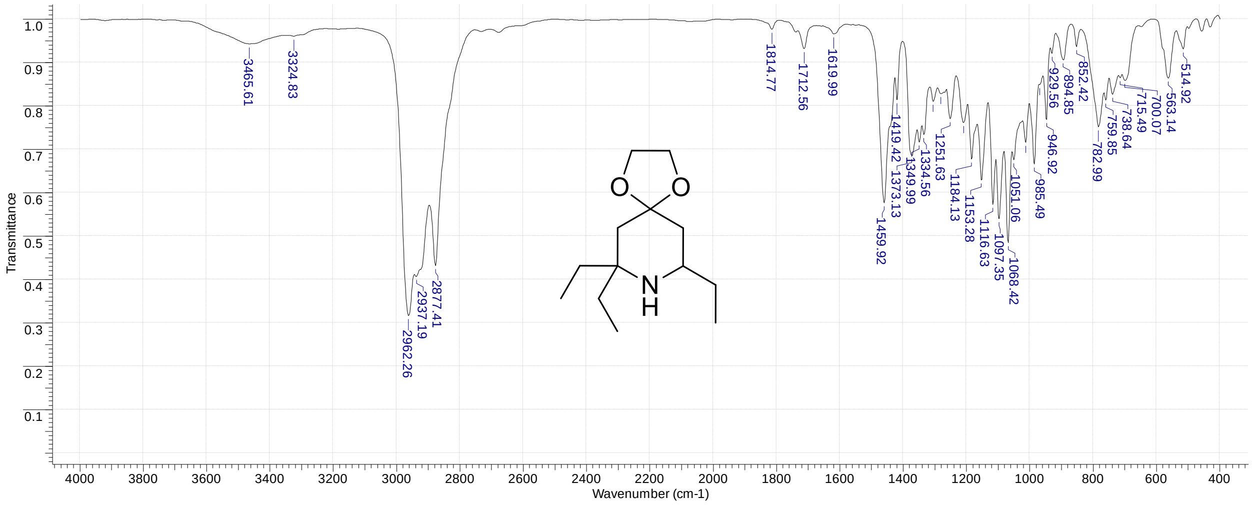

IR (KBr) of 7,7,9-triethyl-9-ethynyl-1,4-dioxa-8-azaspiro[4.5]decane-8-oxyl (**7a**)

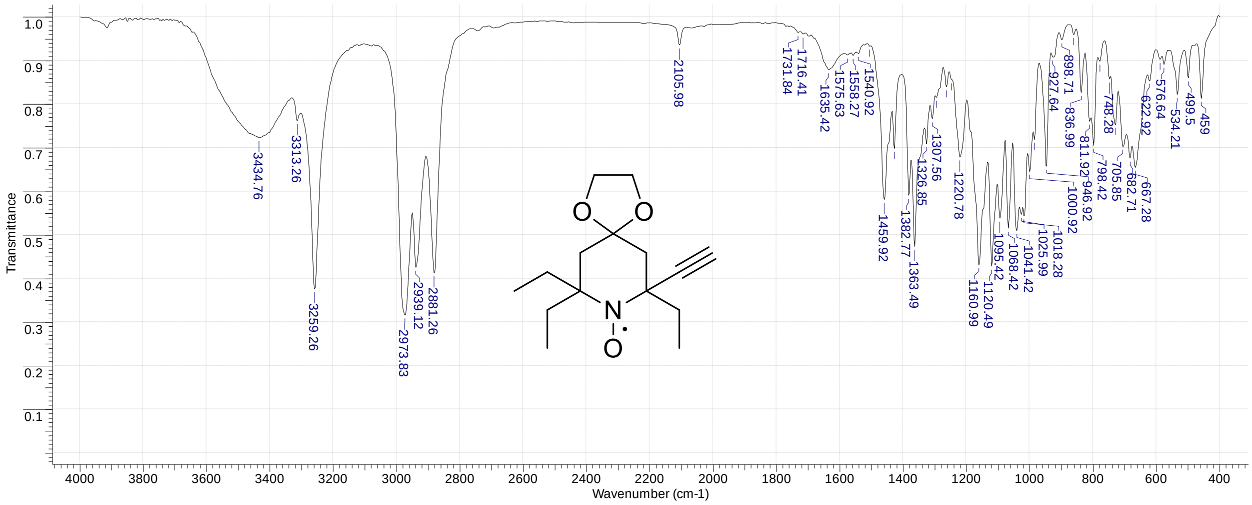

IR (neat) of 7,7,9-triethyl-9-(3-hydroxyprop-1-yn-1-yl)-1,4-dioxa-8-azaspiro[4.5]decan-8-oxyl (**7b**)

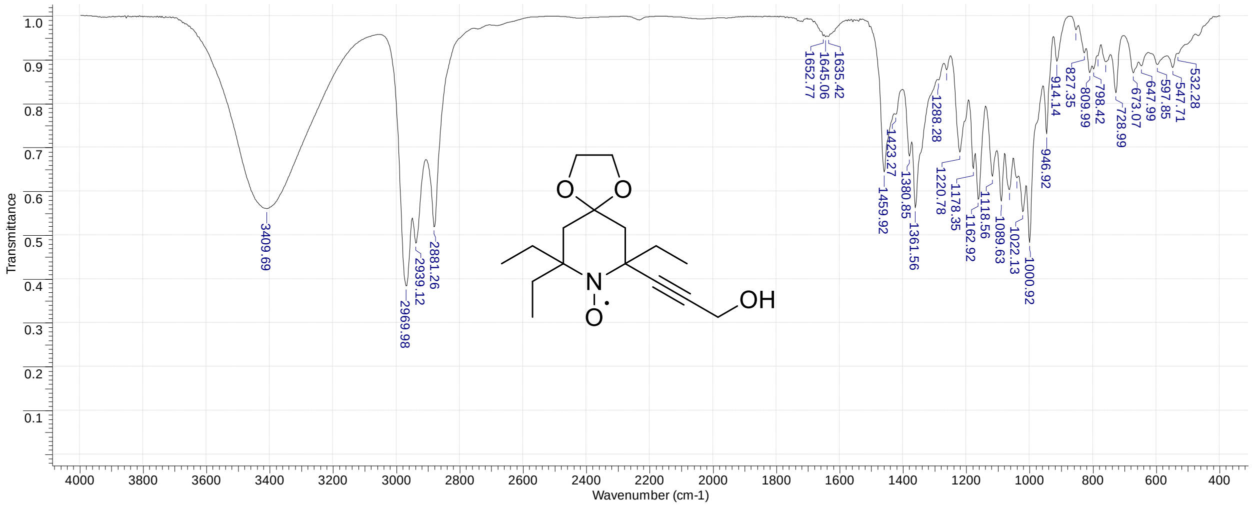

IR (neat) of 7-(3-((*tert*-butoxycarbonyl)amino)prop-1-yn-1-yl)-7,9,9-triethyl-1,4-dioxa-8-azaspiro[4.5]decan-8-oxyl (**7c**)

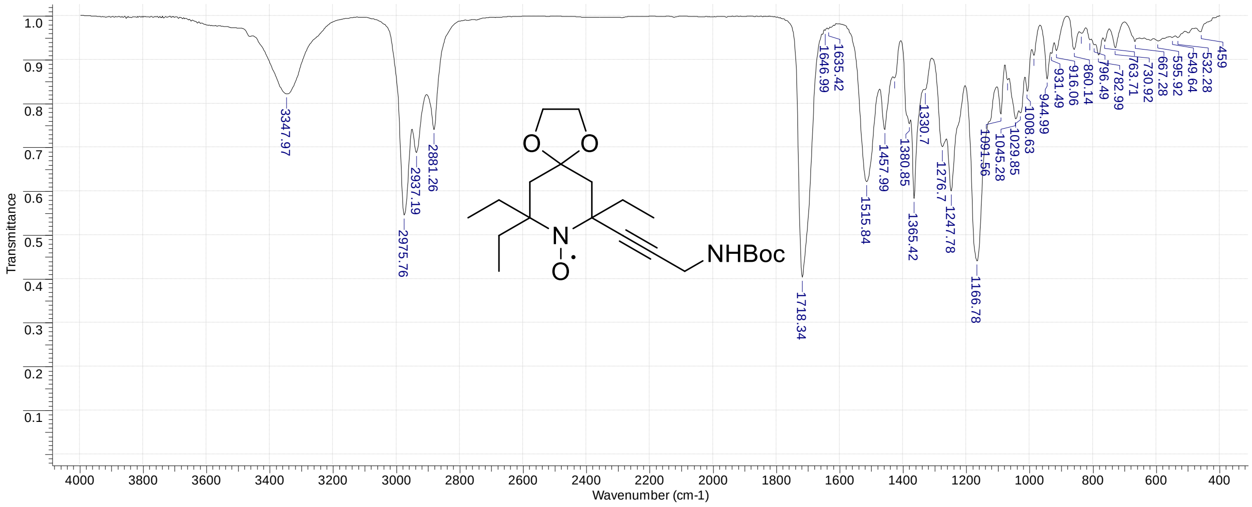

IR (KBr) of 7,7,9,9-tetraethyl -1,4-dioxa-8-azaspiro[4.5]decane-8-oxyl (**8a**)

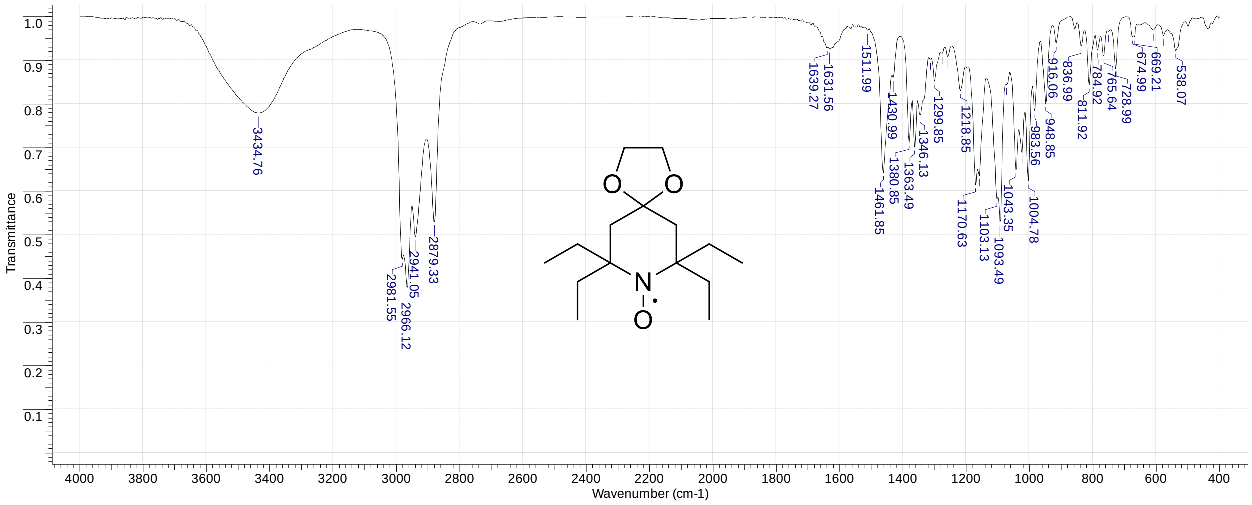

IR (neat) of 7,7,9-triethyl-9-(3-hydroxypropyl)-1,4-dioxa-8-azaspiro[4.5]decan-8-oxyl (**8b**)

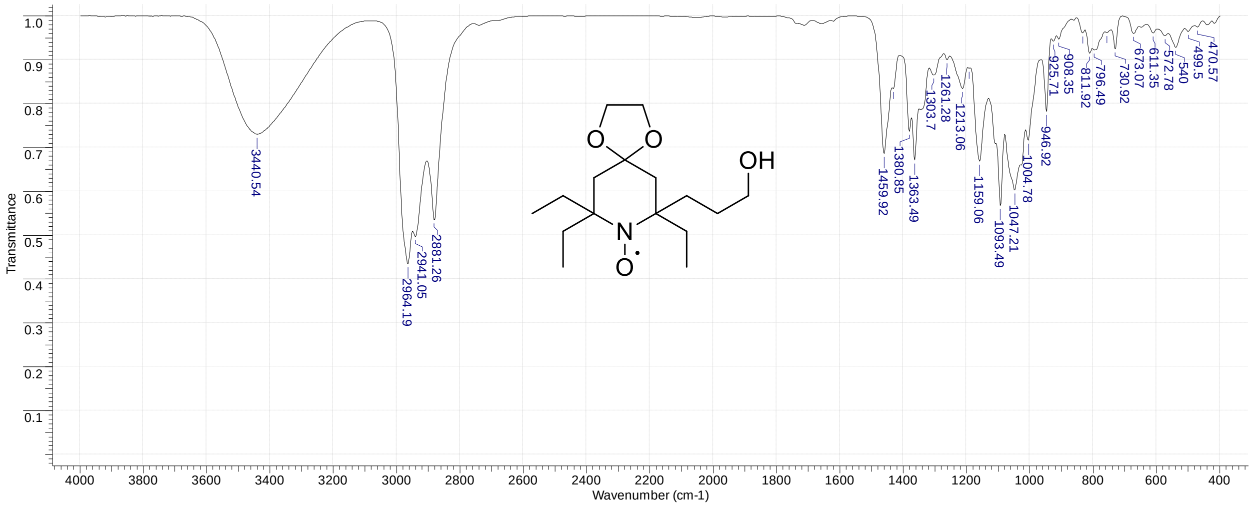

IR (neat) of 7-(3-((*tert*-butoxycarbonyl)amino)propyl)-7,9,9-triethyl-1,4-dioxa-8-azaspiro[4.5]decan-8-oxyl (**8c**)

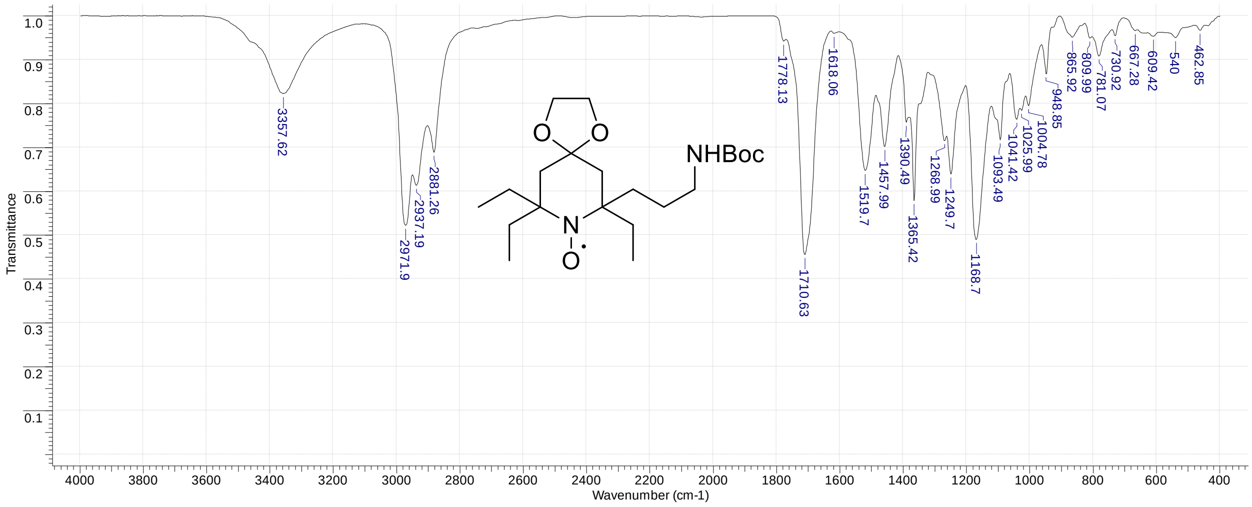

IR (KBr) of 2,2,6,6-tetraethylpiperidin-4-one-1-oxyl (**9a**)

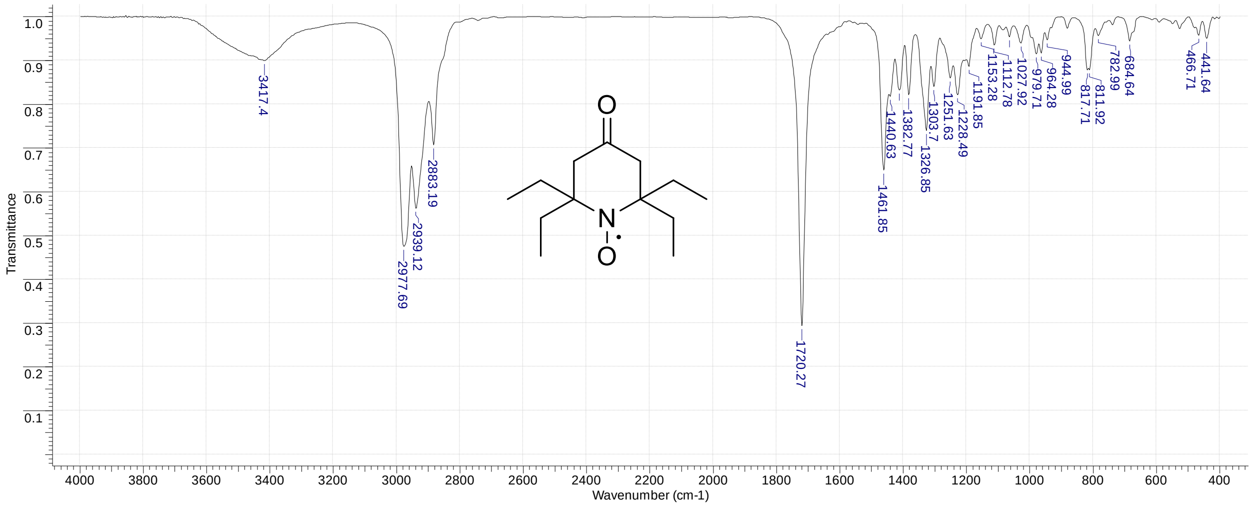

IR (neat) of 7,7,9-tripropyl-1,4-dioxa-8-azaspiro[4.5]decane (**5b**)

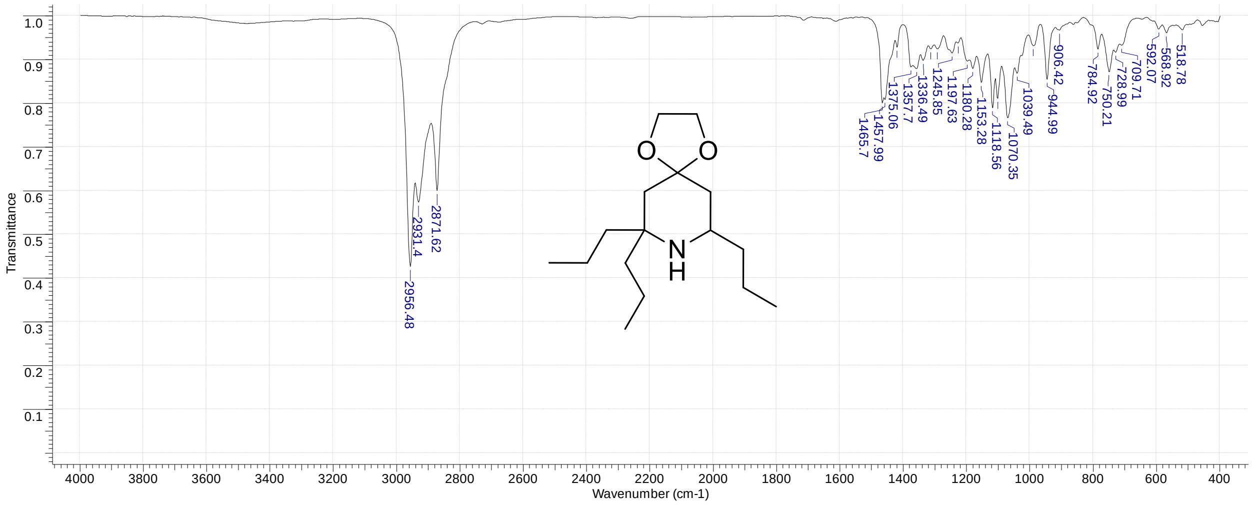

IR (neat) of 7,7,9-triprorpyl-9-(prop-2-en-1-yl)-1,4-dioxa-8-azaspiro[4.5]decane-8-oxyl (**7d**)

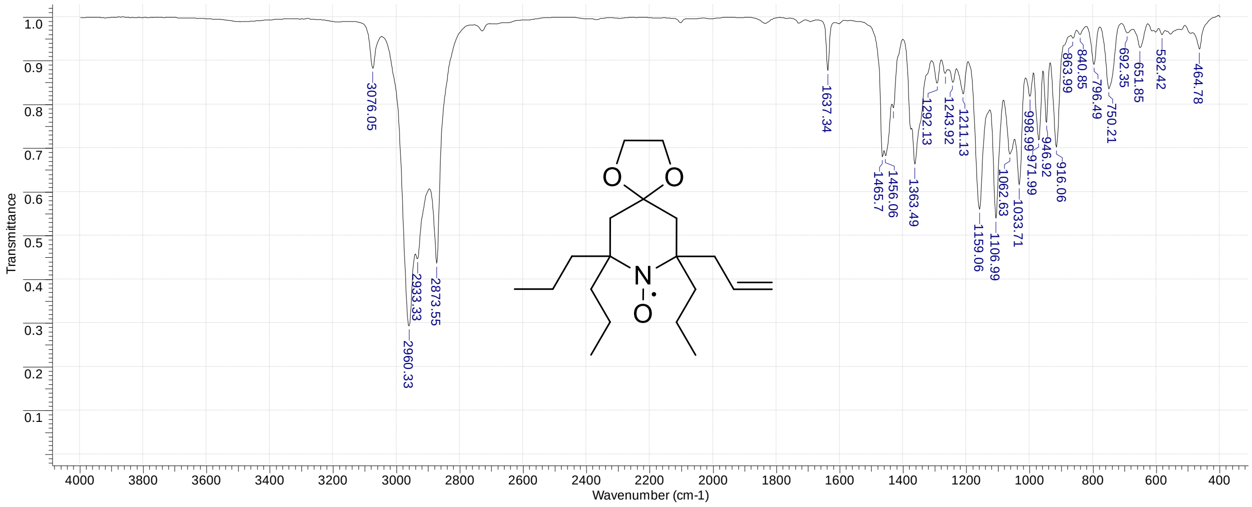

IR (neat) of 7,7,9,9-tetrapropyl-1,4-dioxa-8-azaspiro[4.5]decane-8-oxyl (**8d**)

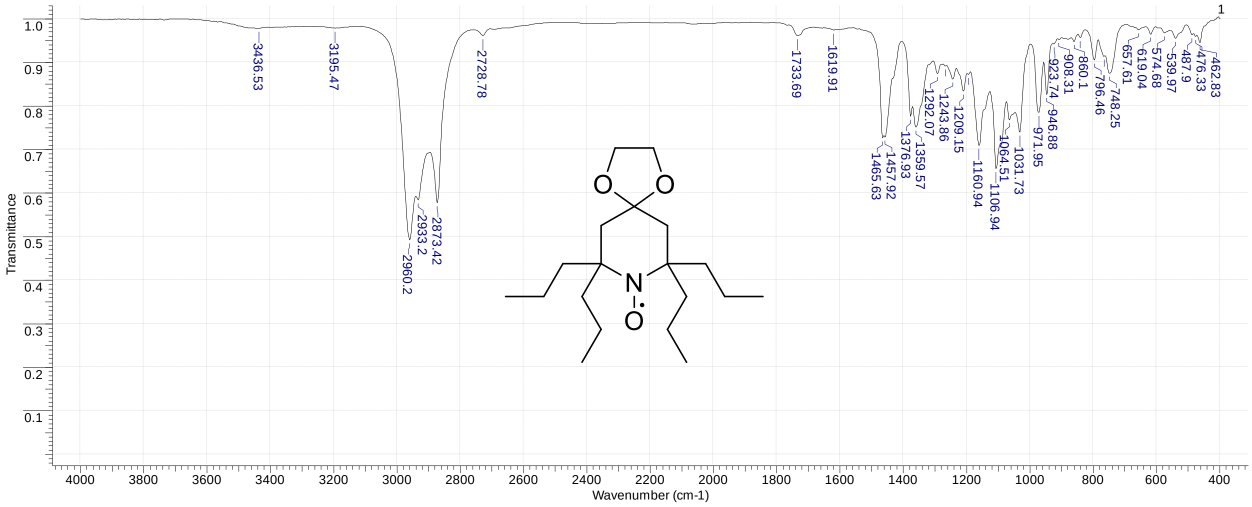

IR (KBr) of 2,2,6,6-tetrapropylpiperidin-4-one-1-oxyl (**9d**)

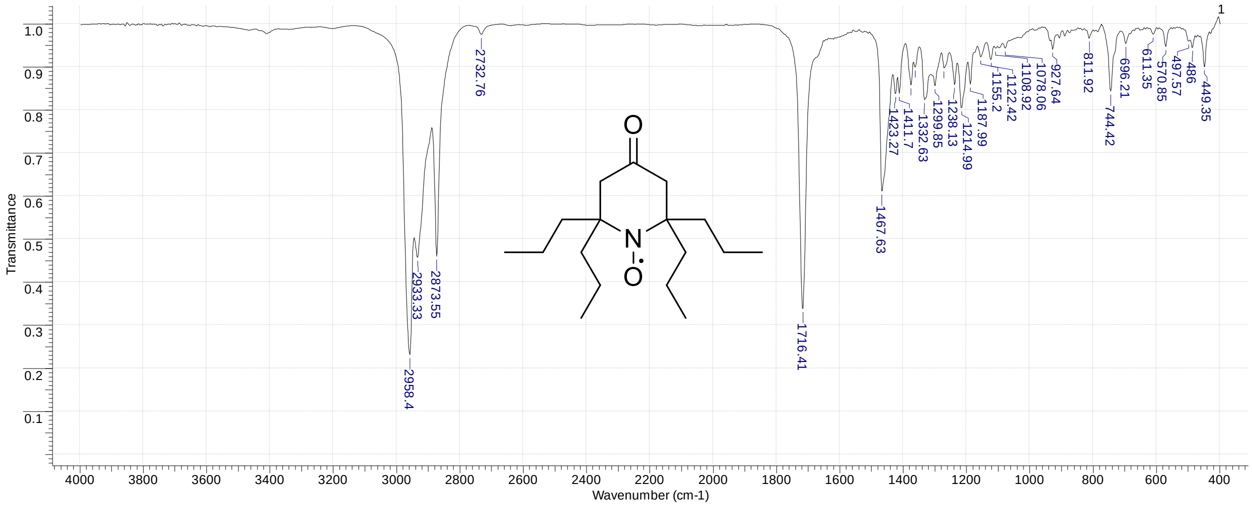

Gas chromatography–mass spectrometry

GC–MS of 3-(1,3-dioxoisoindolin-2-yl)-hexanone-5 (**2a**)

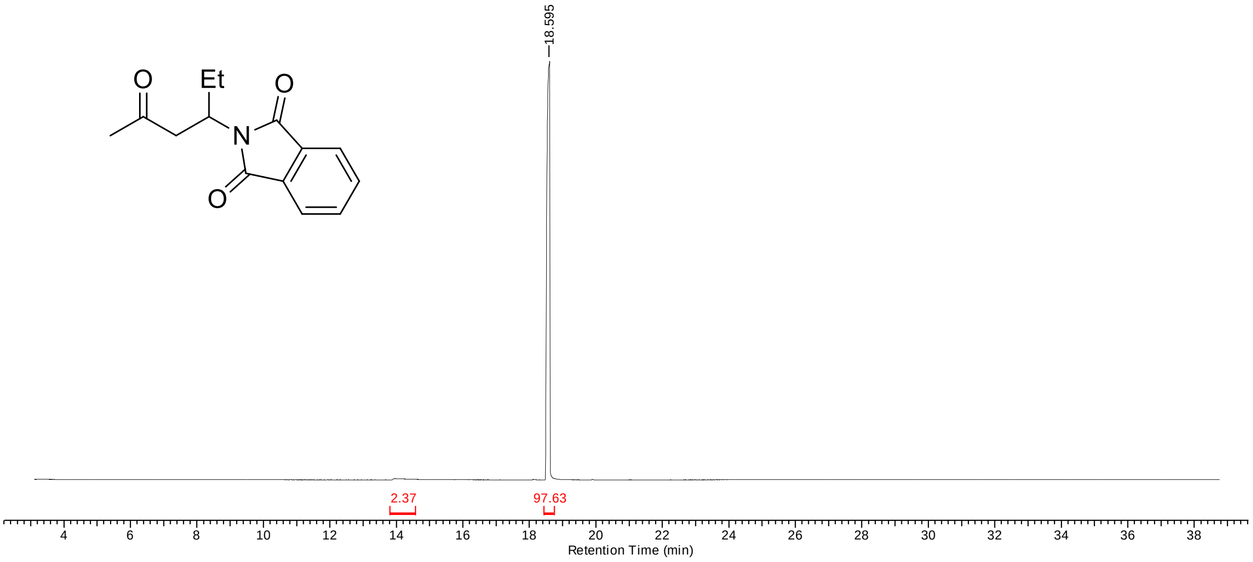

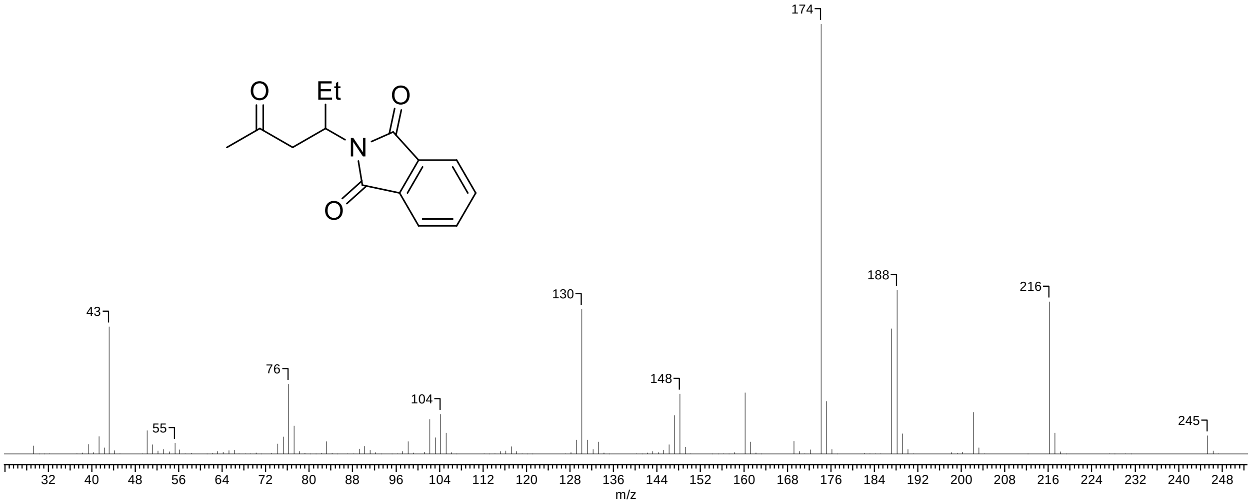

GC–MS of 1-(2-methyl-1,3-dioxolan-2-yl)-2-(1,3-dioxoisoindolin-2-yl)butane (**3a**)

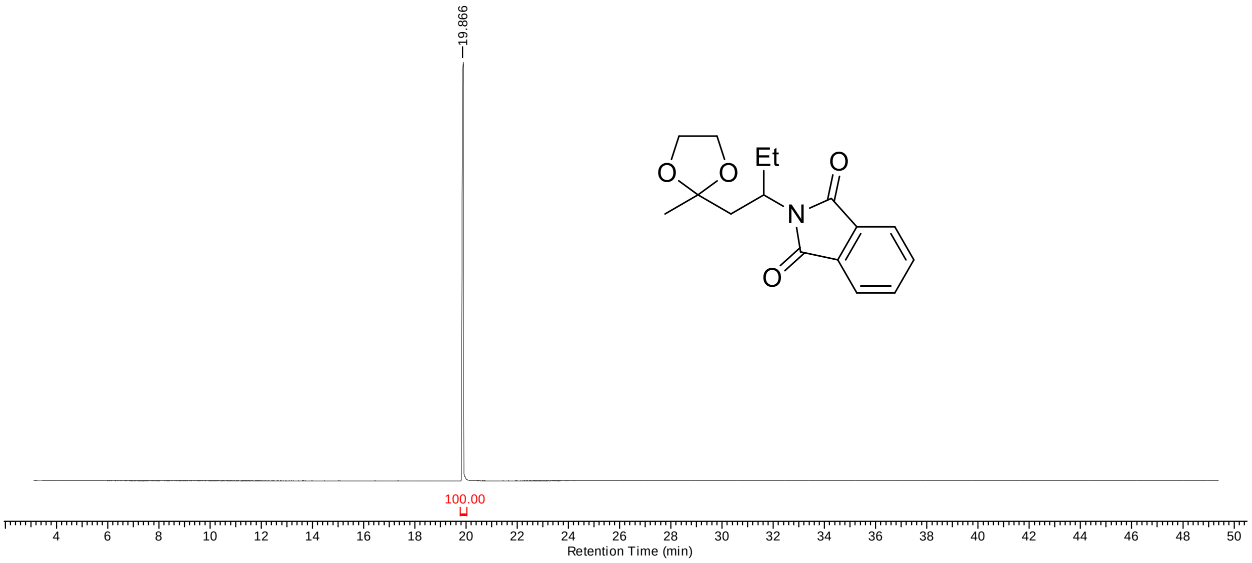

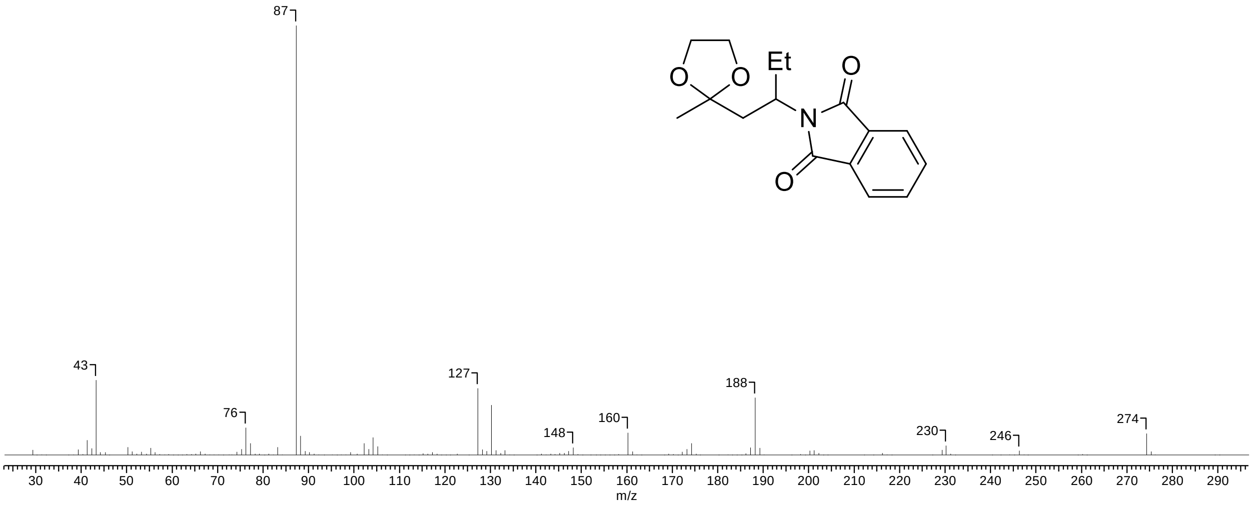

GC–MS of 1-(2-methyl-1,3-dioxolan-2-yl)butan-2-amine (**4a**)

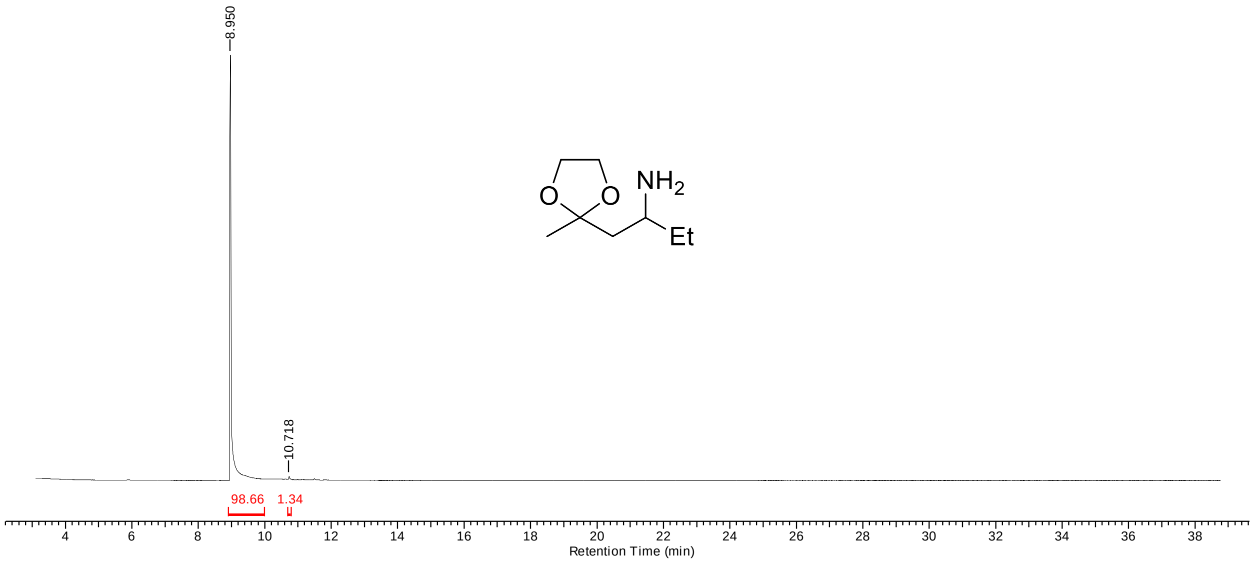

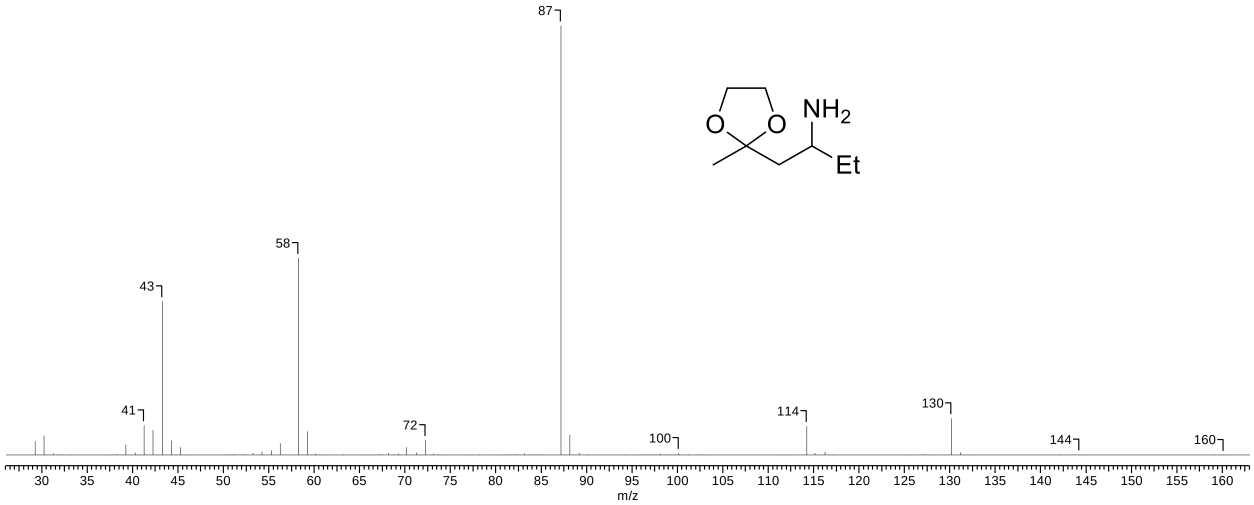

GC–MS of 4-(1,3-dioxoisoindolin-2-yl)-heptanone-6 (**2b**).

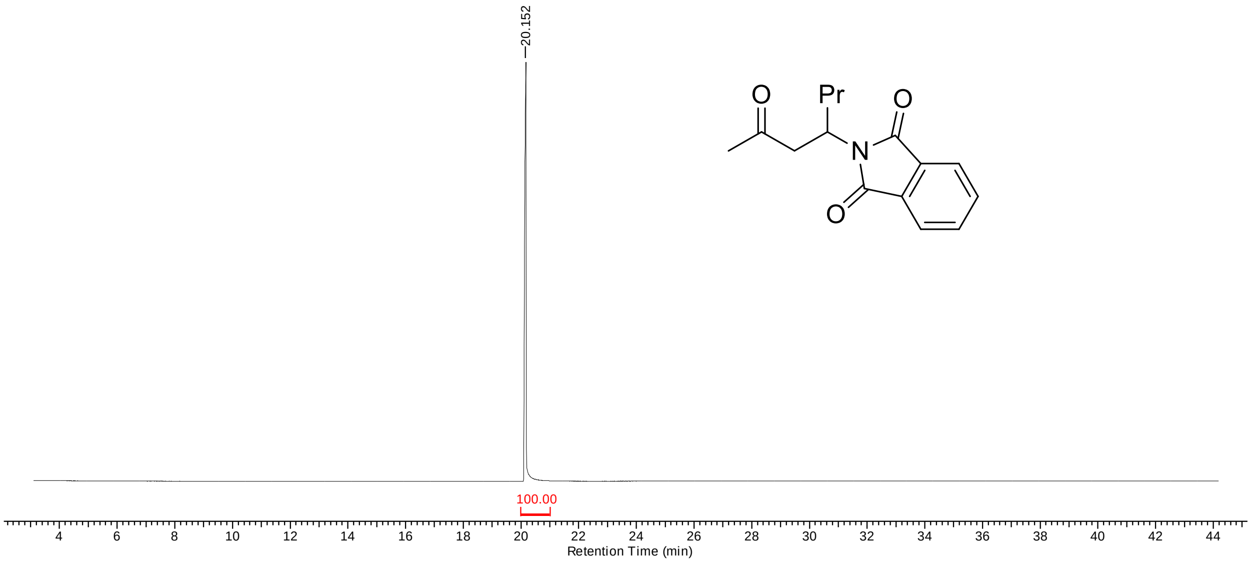

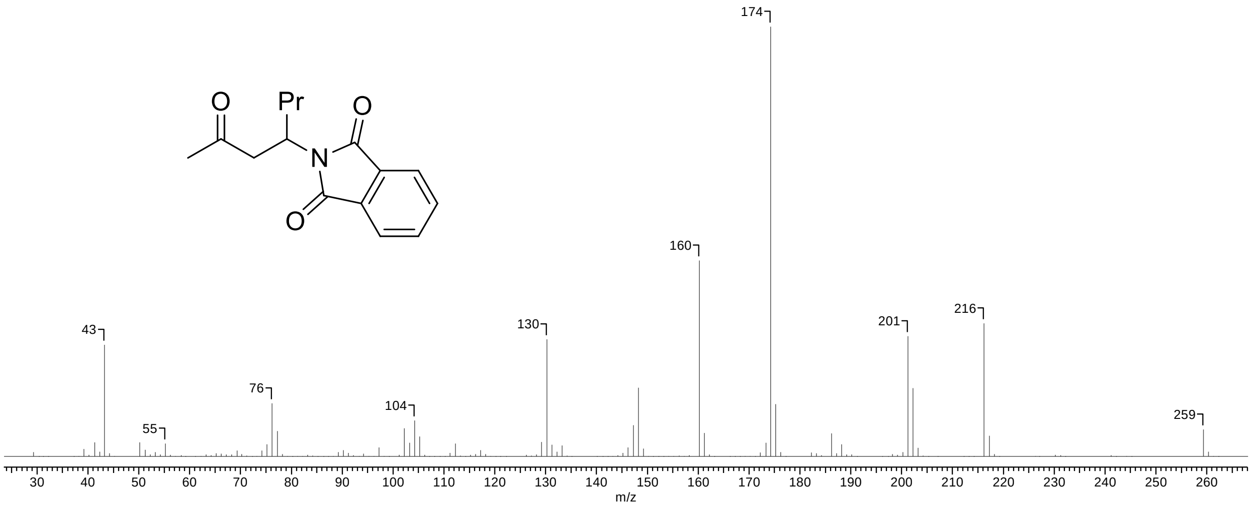

GC–MS of 1-(2-methyl-1,3-dioxolan-2-yl)-2-(1,3-dioxoisoindolin-2-yl)pentane (**3b**).

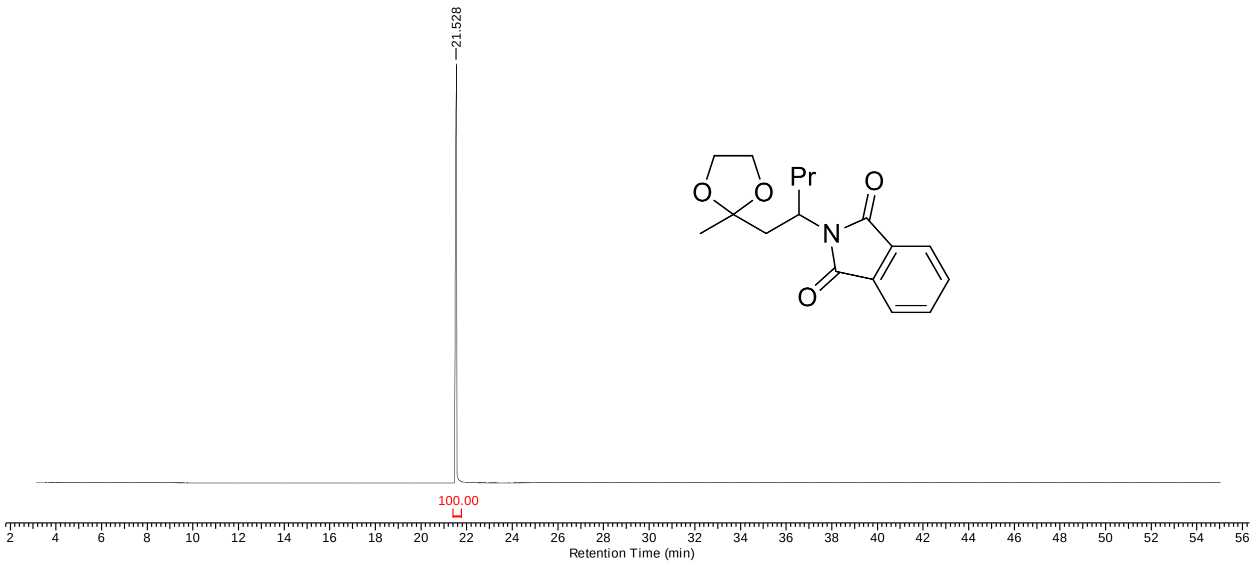

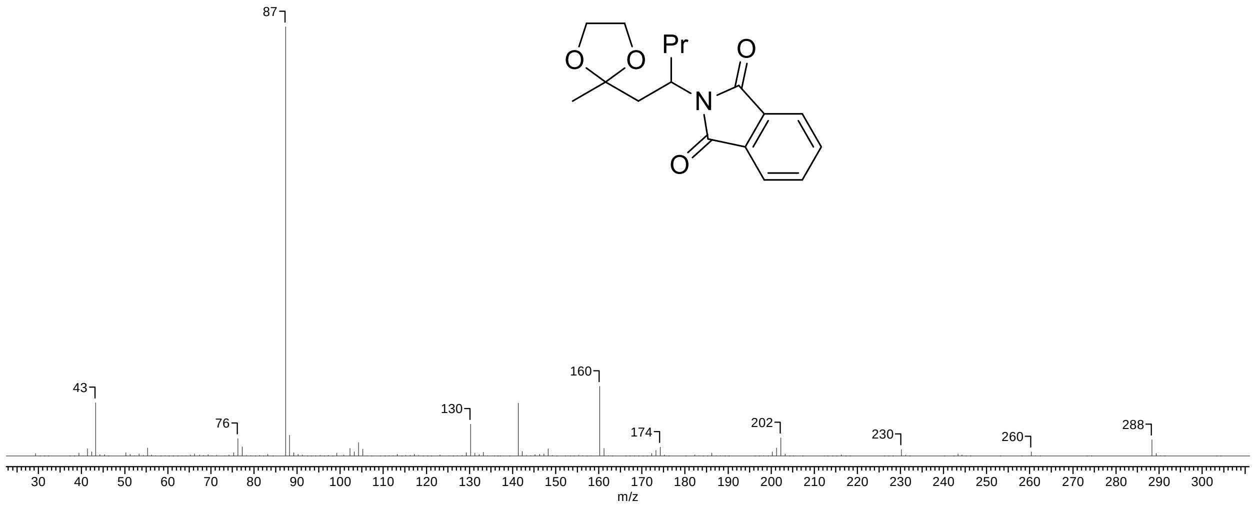

GC–MS of 1-(2-methyl-1,3-dioxolan-2-yl)pentan-2-amine (**4b**)

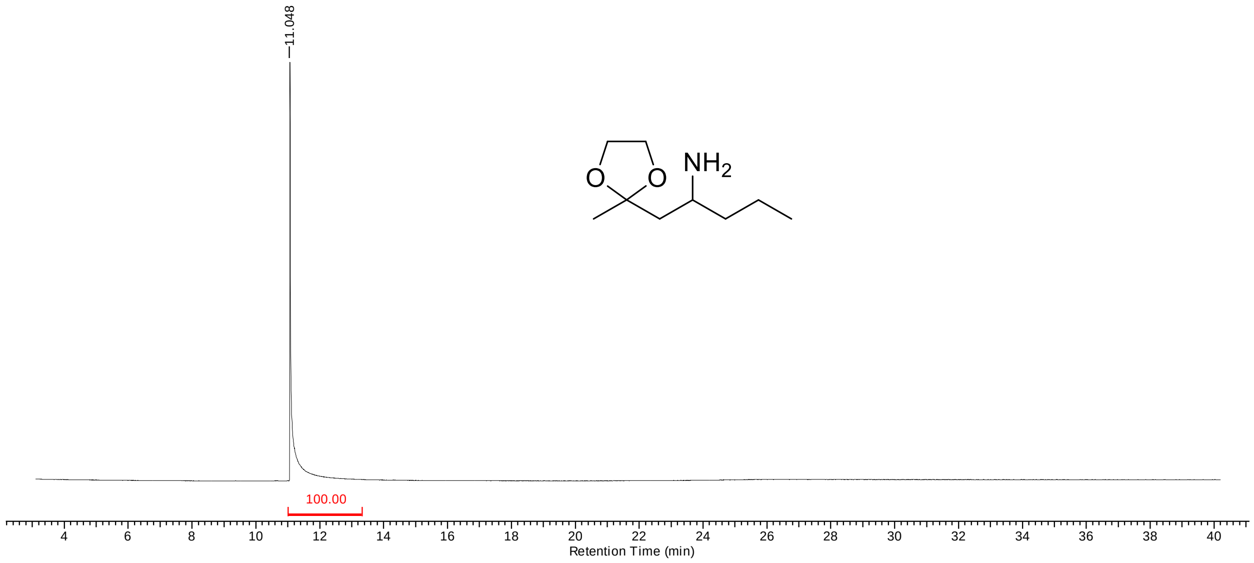

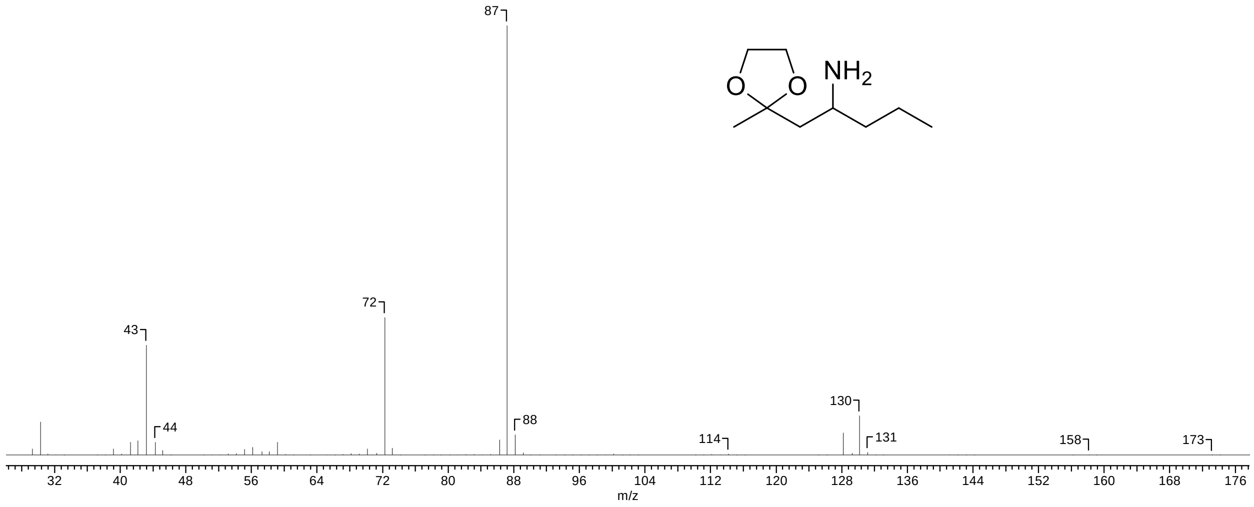

GC–MS of 7,7,9-triethyl-1,4-dioxa-8-azaspiro[4.5]decane (**5a**)

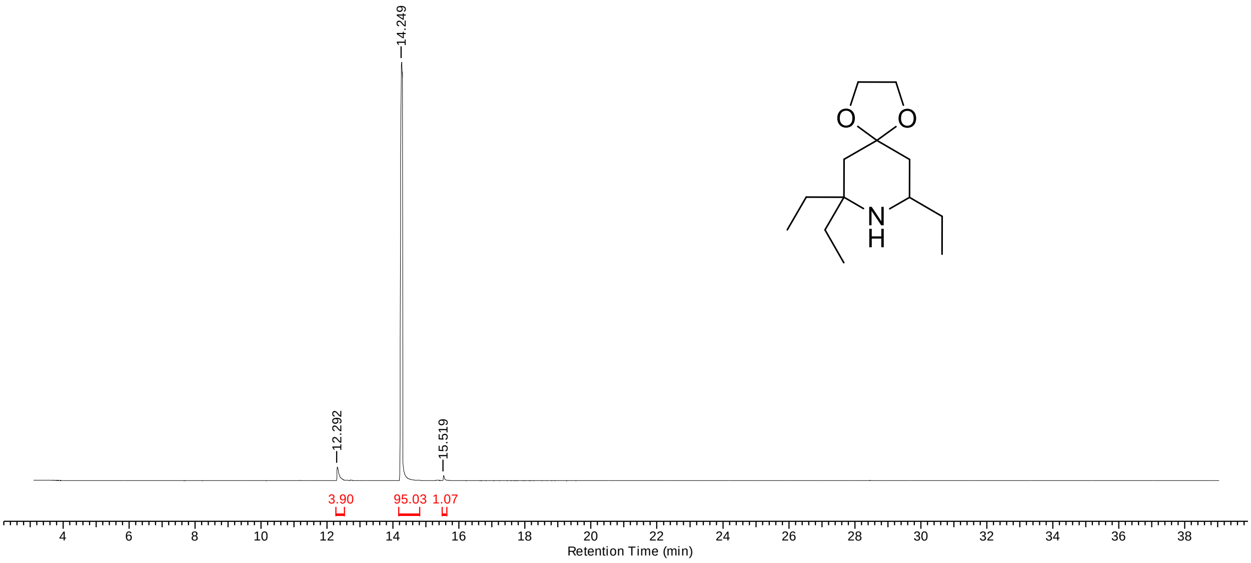

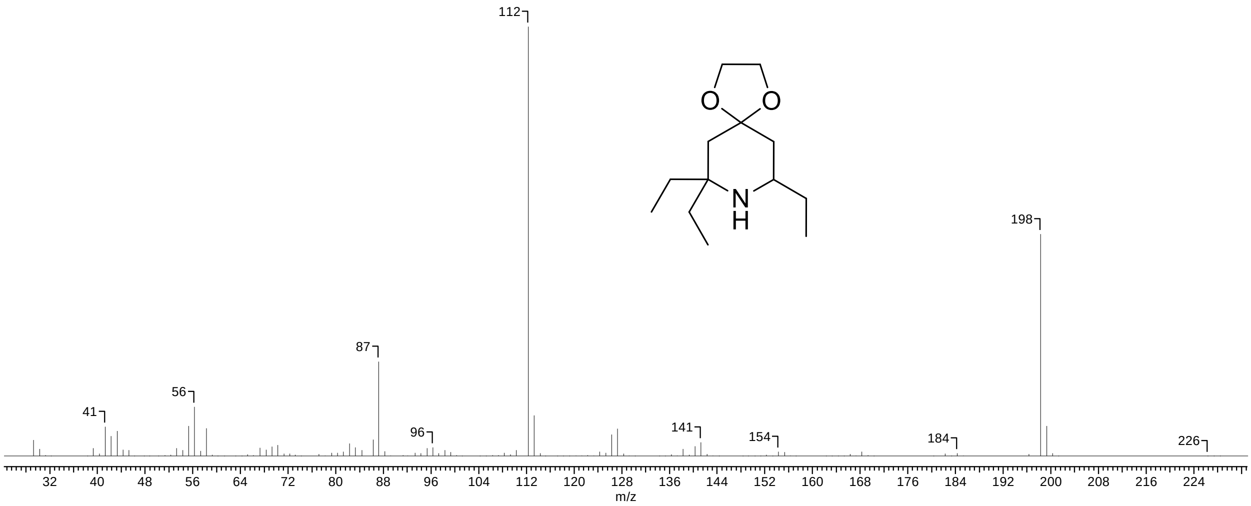

GC–MS of 7,7,9-tripropyl-1,4-dioxa-8-azaspiro[4.5]decane (**5b**)

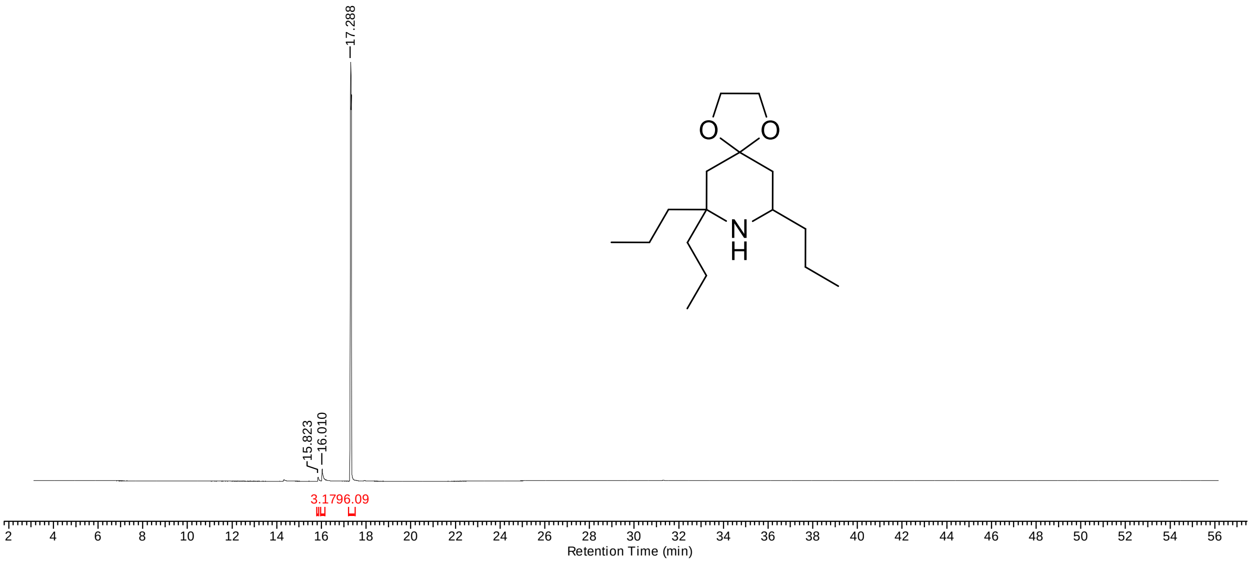

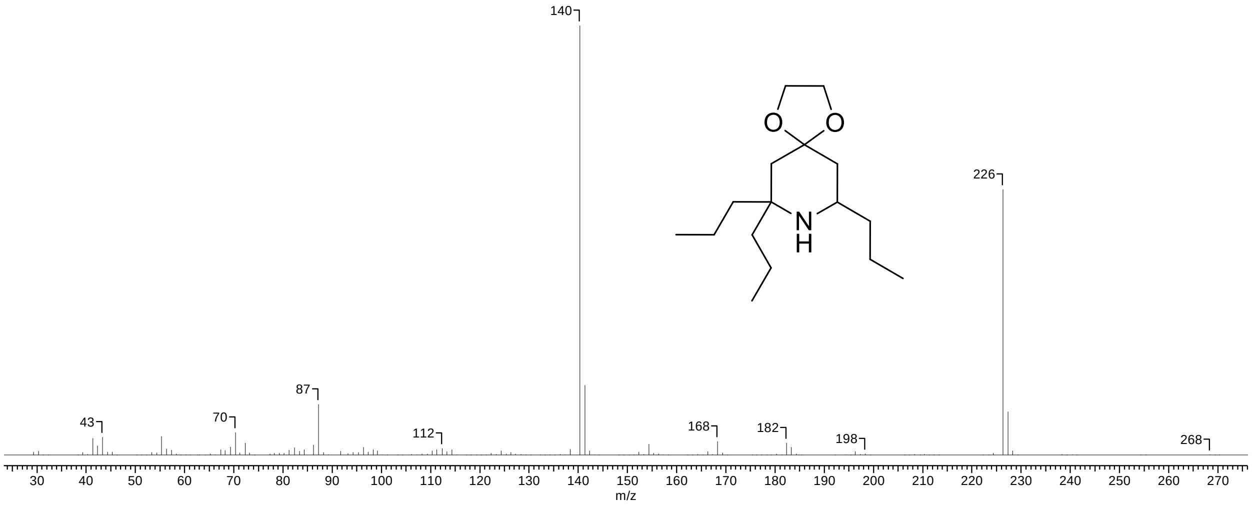

EPR measurements

EPR spectrum of 7,7,9-triethyl-9-ethynyl-1,4-dioxa-8-azaspiro[4.5]decane-8-oxyl (**7a**)

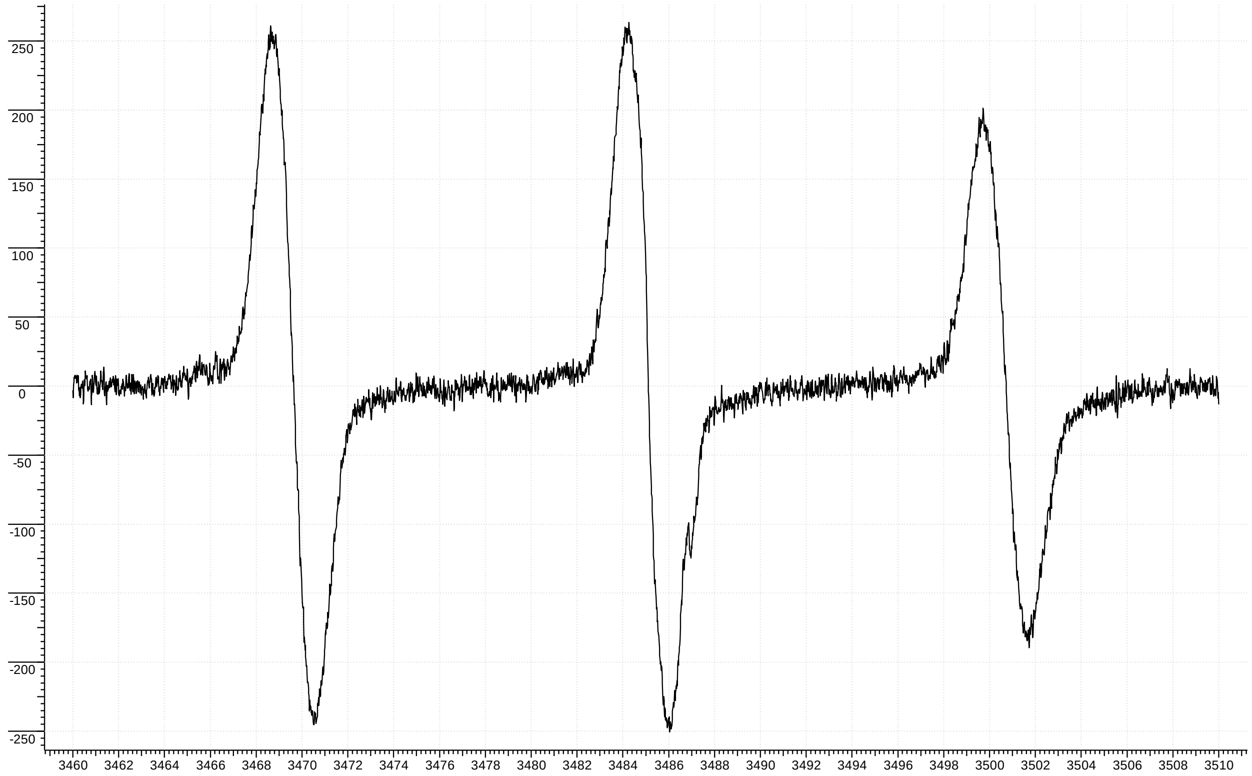

EPR spectrum of 7,7,9-triethyl-9-(3-hydroxyprop-1-yn-1-yl)-1,4-dioxa-8-azaspiro[4.5]decan-8-oxyl (**7b**)

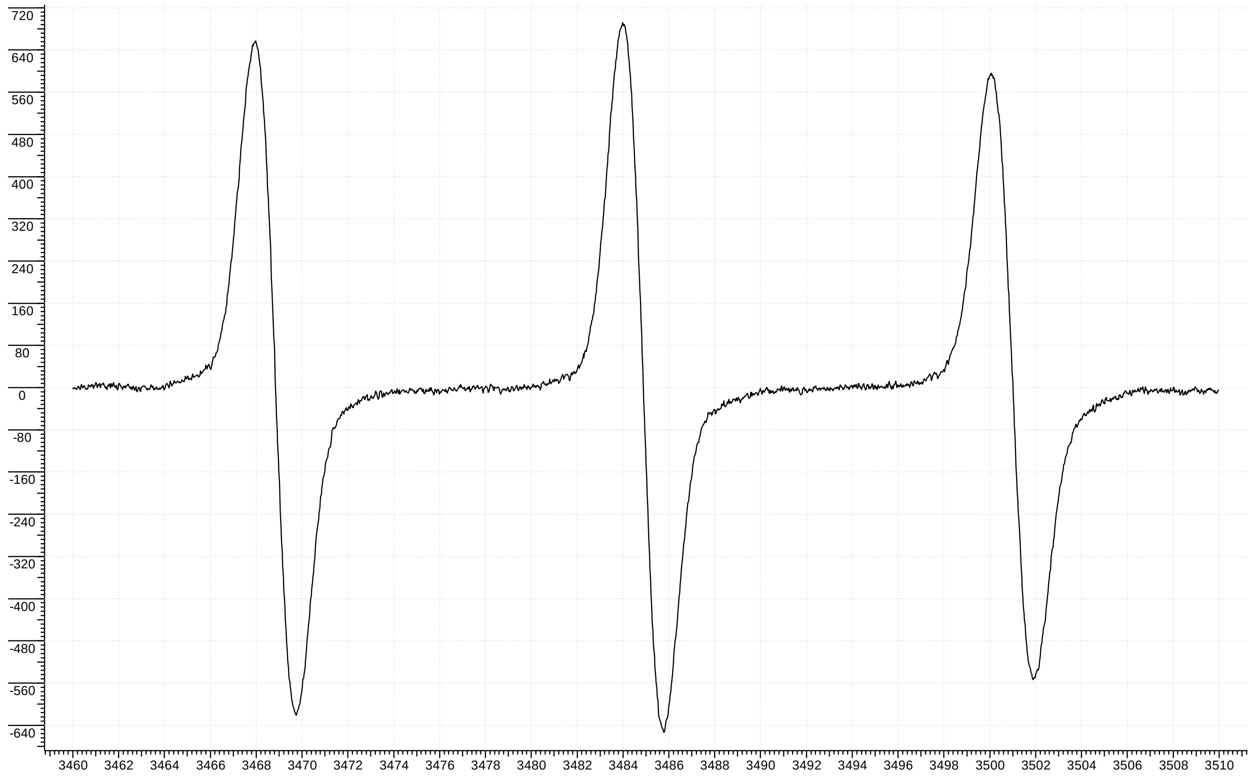

EPR spectrum of 7-(3-((tert-butoxycarbonyl)amino)prop-1-yn-1-yl)-7,9,9-triethyl-1,4-dioxa-8-azaspiro[4.5]decan-8-oxyl (**7c**)

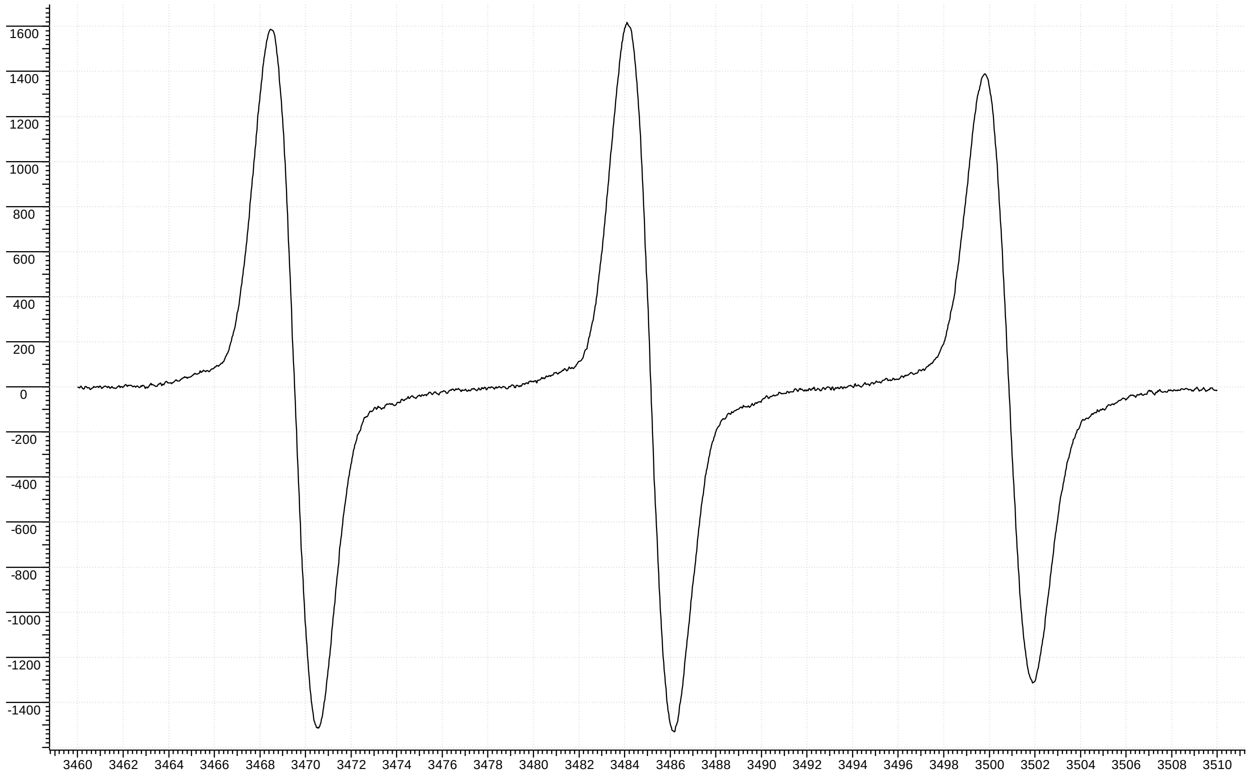

EPR spectrum of 7,7,9-triprorpyl-9-(prop-2-en-1-yl)-1,4-dioxa-8-azaspiro[4.5]decane-8-oxyl (**7d**)

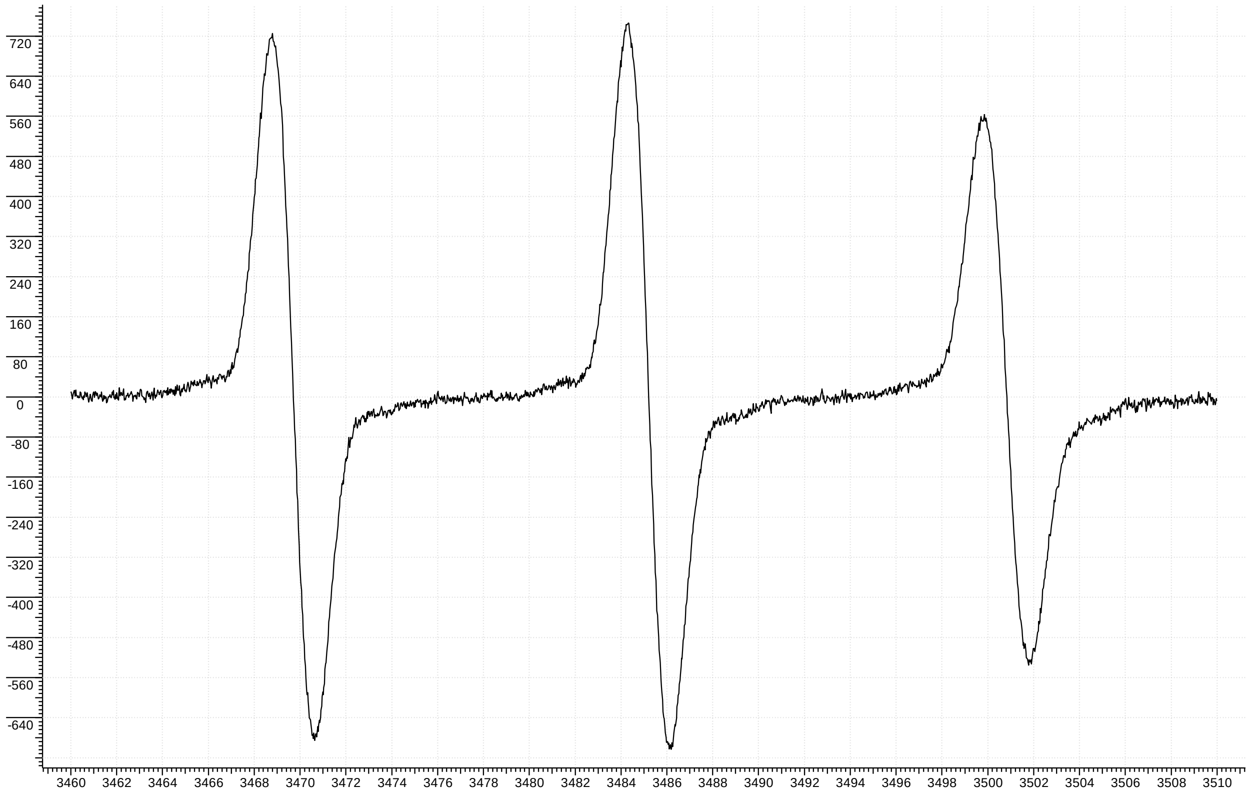

EPR spectrum of 7,7,9,9-tetraethyl-1,4-dioxa-8-azaspiro[4.5]decane-8-oxyl (**8a**)

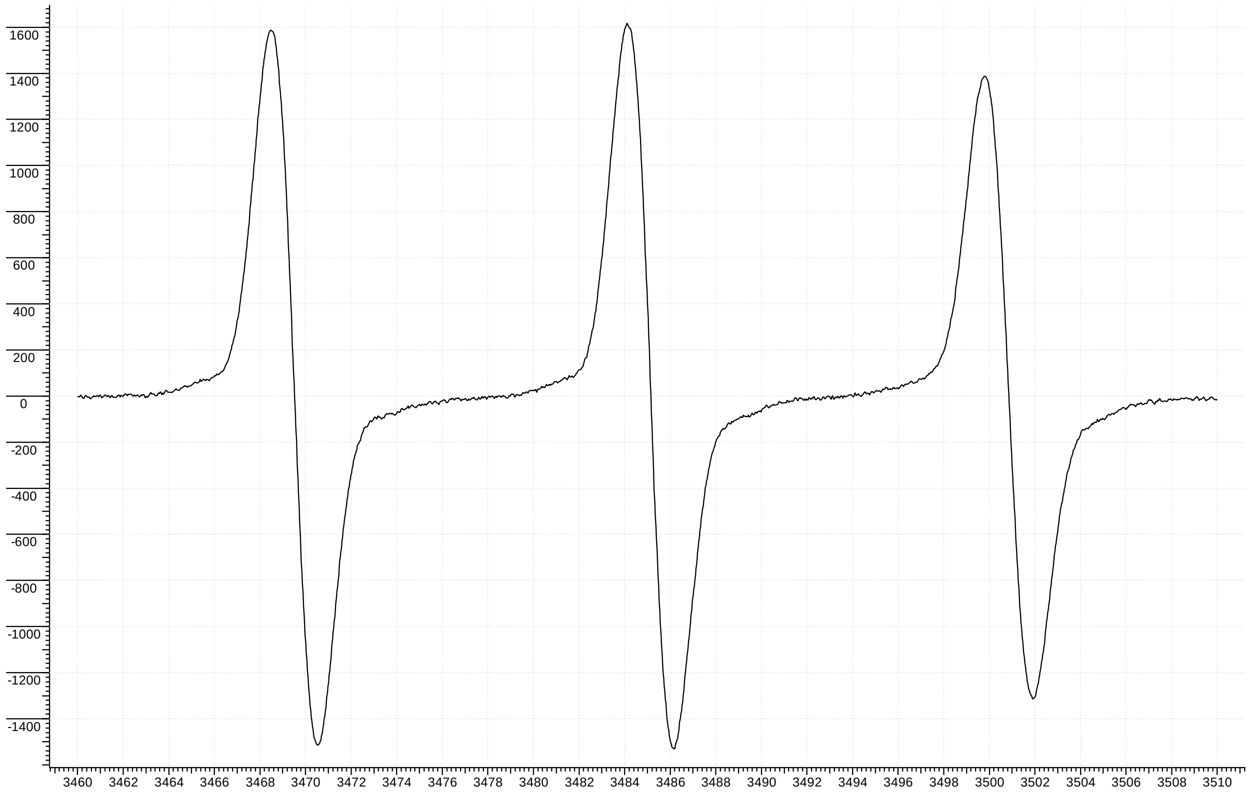

EPR spectrum of 7,7,9-triethyl-9-(3-hydroxypropyl)-1,4-dioxa-8-azaspiro[4.5]decan-8-oxyl (**8b**)

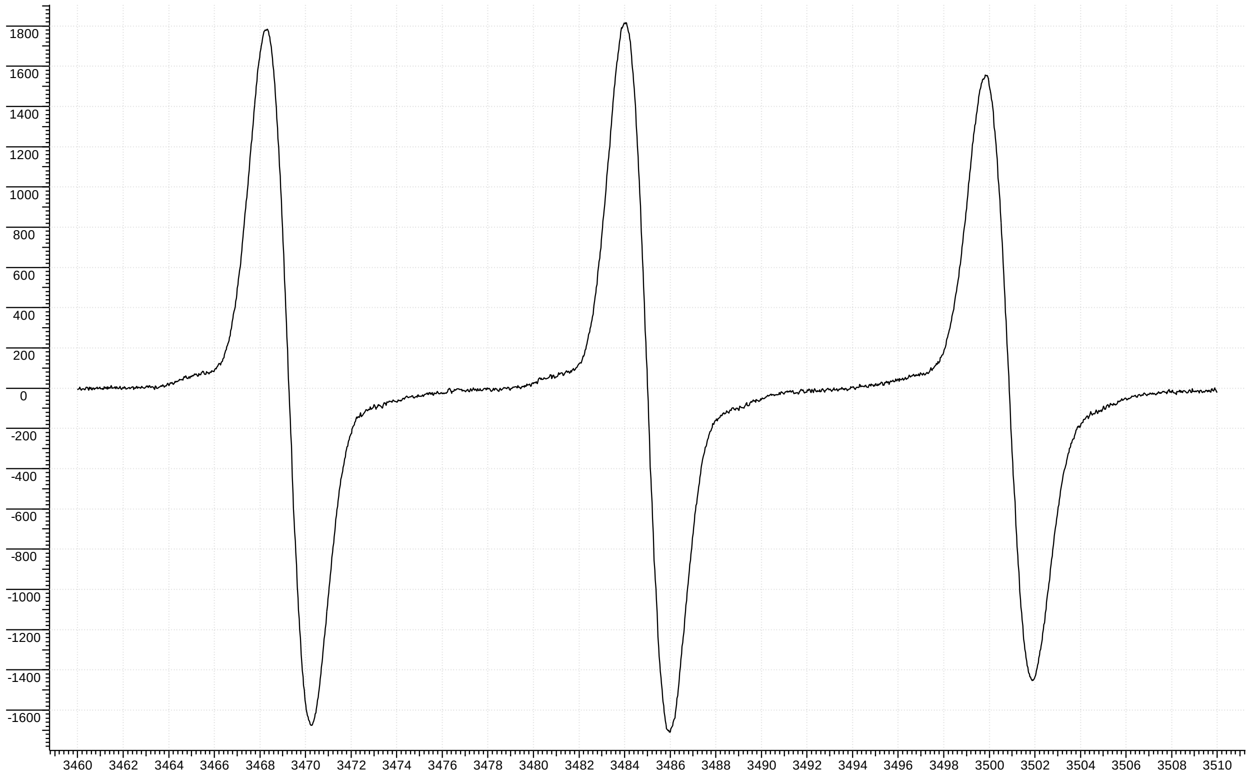

EPR spectrum of 7-(3-((*tert*-butoxycarbonyl)amino)propyl)-7,9,9-triethyl-1,4-dioxa-8-azaspiro[4.5]decan-8-oxyl (**8c**)

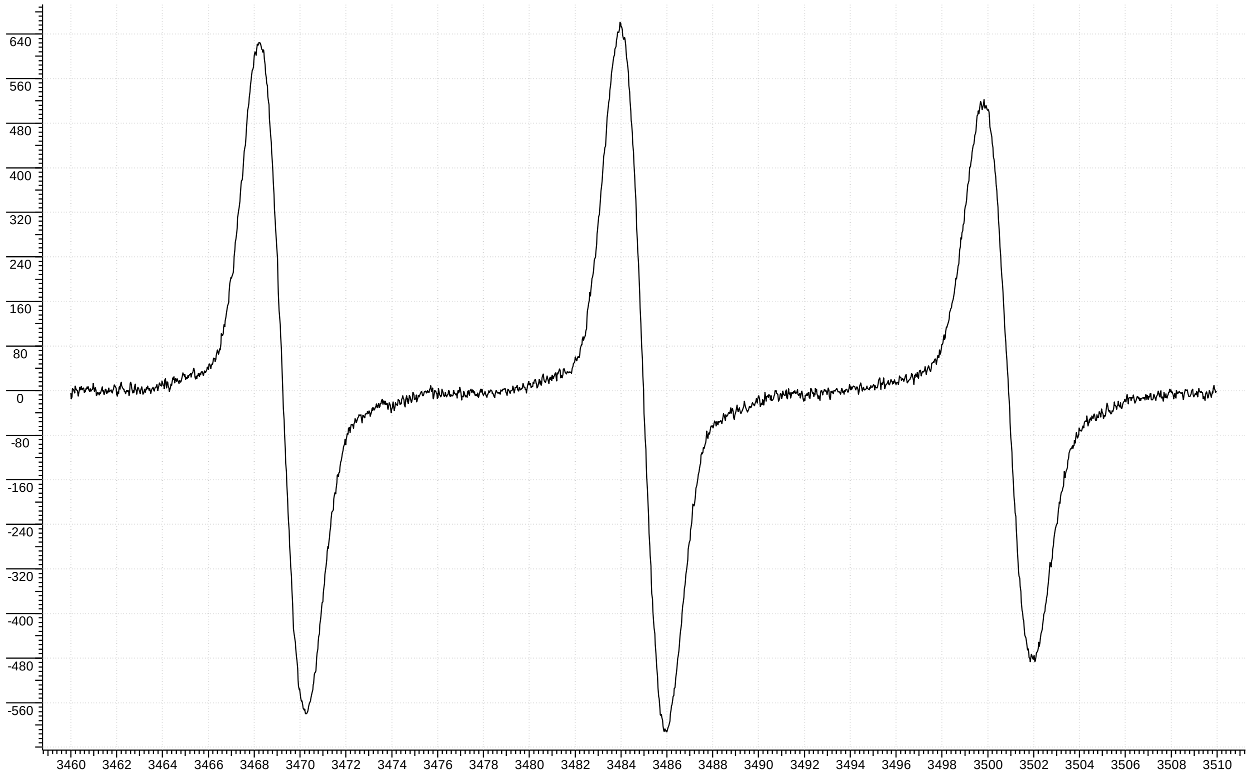

EPR spectrum of 7,7,9,9-tetrapropyl-1,4-dioxa-8-azaspiro[4.5]decane-8-oxyl (**8d**)

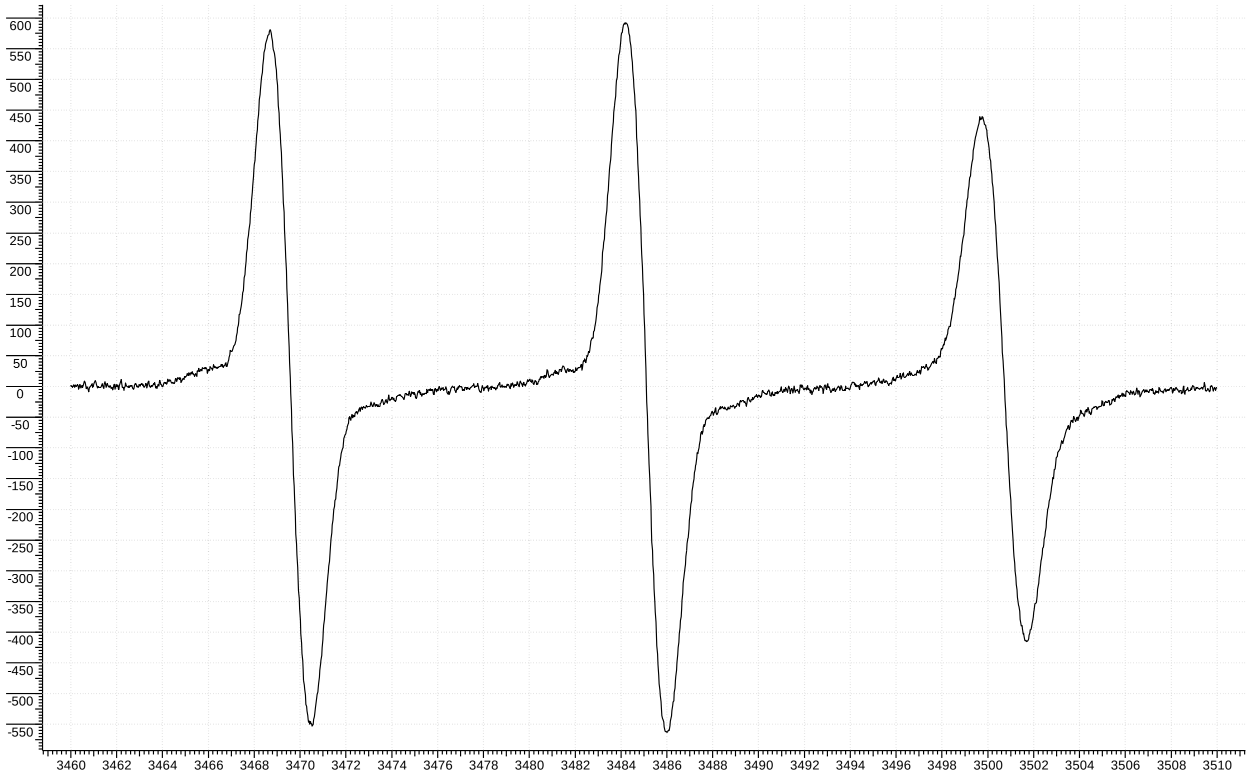

EPR spectrum of 2,2,6,6-tetraethylpiperidin-4-one-1-oxyl (**9a**)

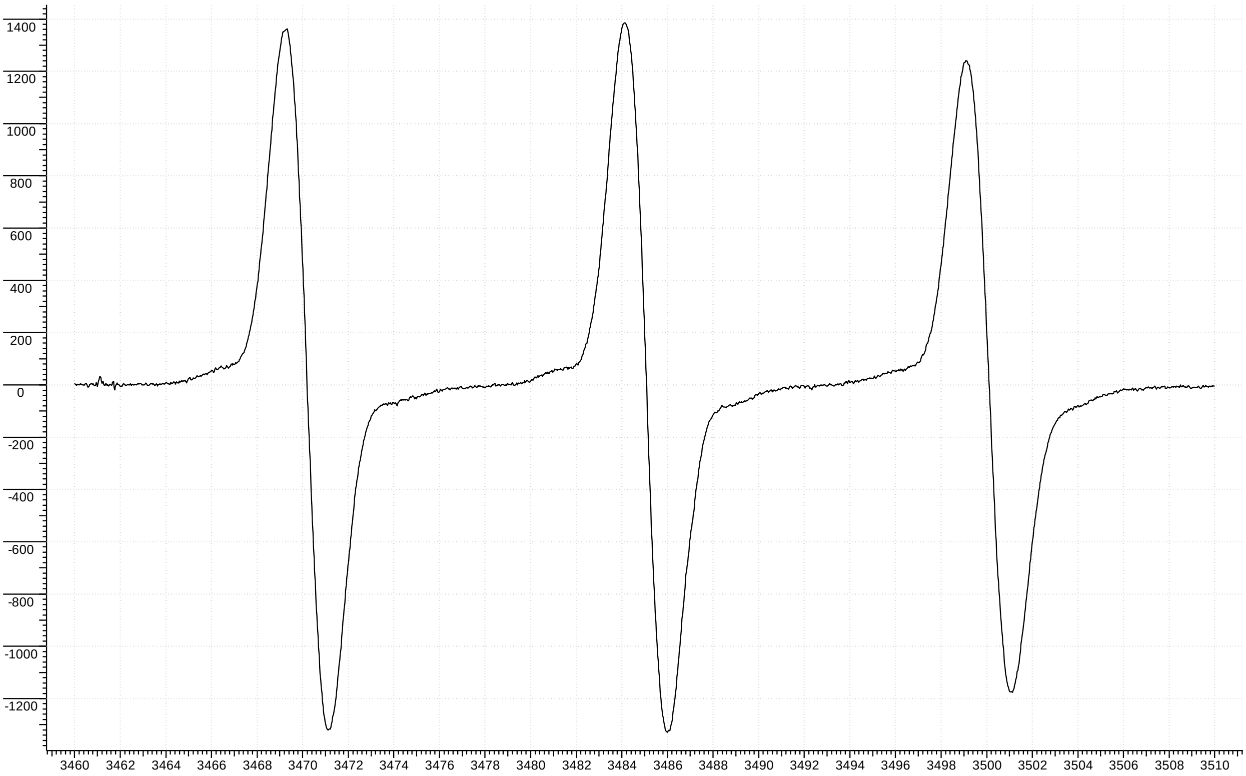

EPR spectrum of 2,2,6,6-tetrapropylpiperidin-4-one-1-oxyl (**9d**)

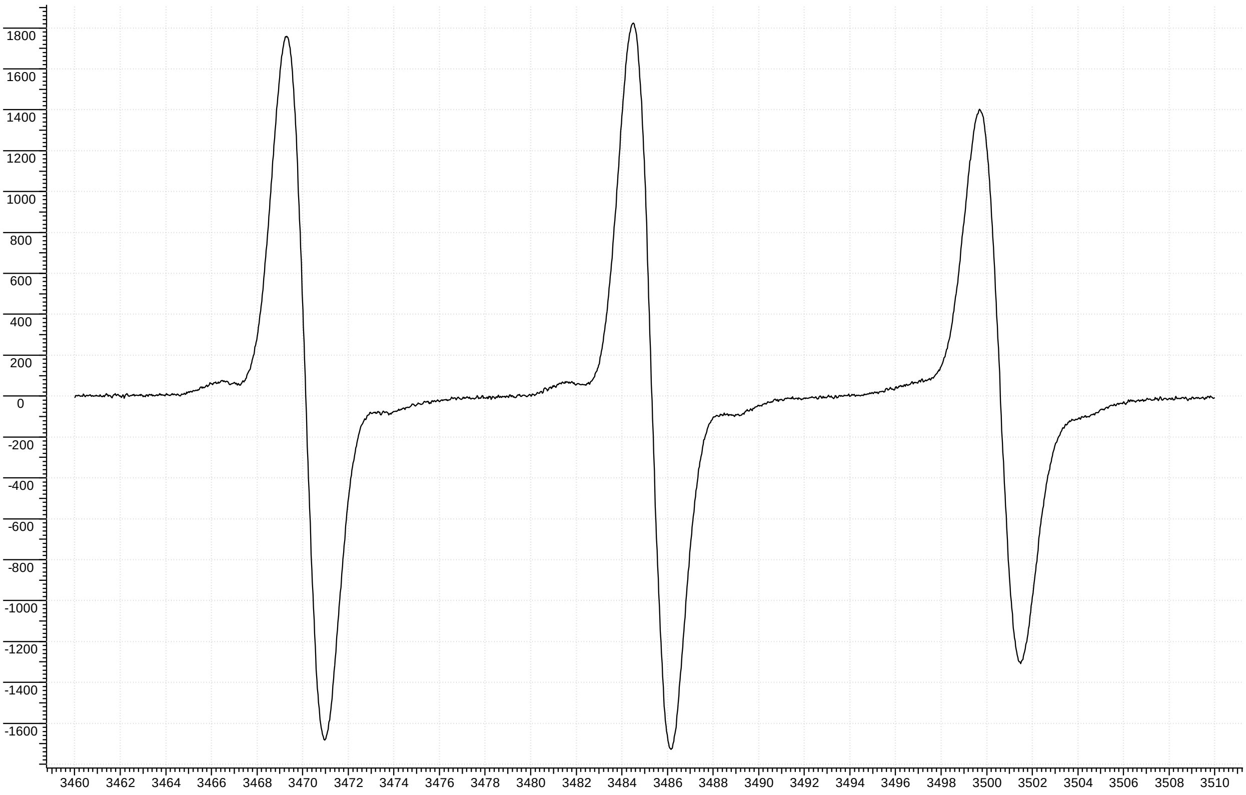

Table S5. Reduction rate constants for nitroxide **7a**,**d**; **8a**,**d**; **9a**,**d**

<table>
<tr><th></th><th>Reduction kinetics of nitroxides</th><th>Second-order reduction constants</th></tr>
<tr><td>7a</td><td>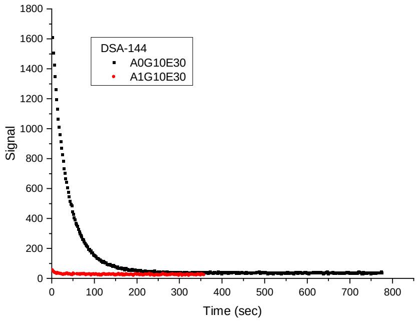
</td><td></td></tr>
<tr><td>7d</td><td>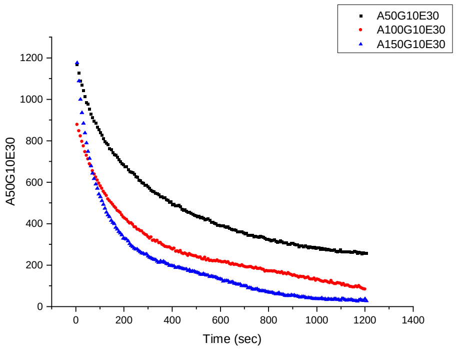
</td><td>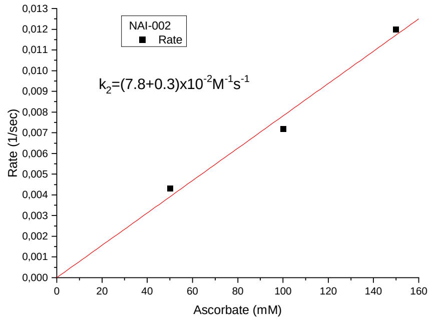
</td></tr>
<tr><td>8a</td><td>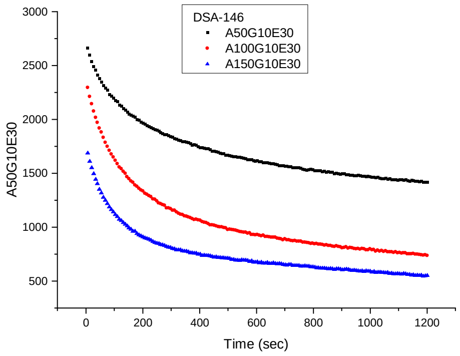
</td><td>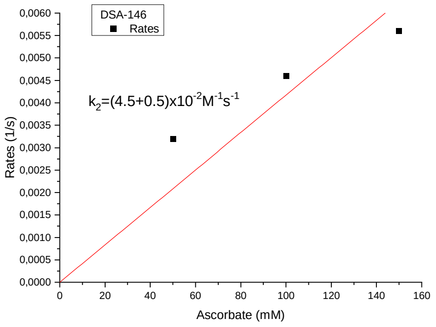
</td></tr>
<tr><td>8d</td><td>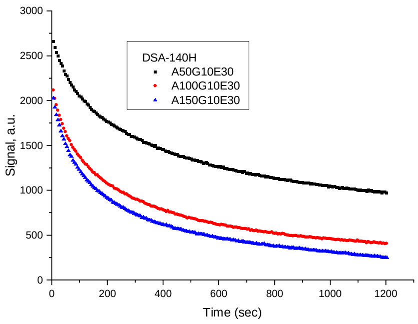
</td><td>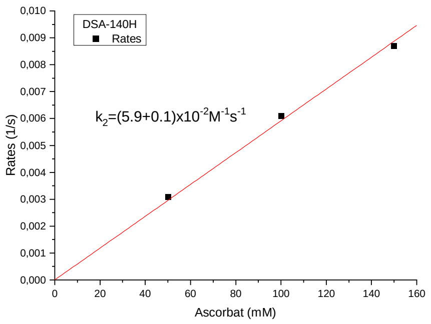
</td></tr>
</table>

9a

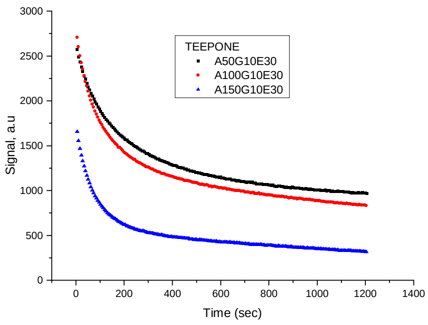
TEEPONE
A50G10E30
A100G10E30
A150G10E30
Signal, a.u
Time (sec)
0
500
1000
1500
2000
2500
3000
0
200
400
600
800
1000
1200
1400

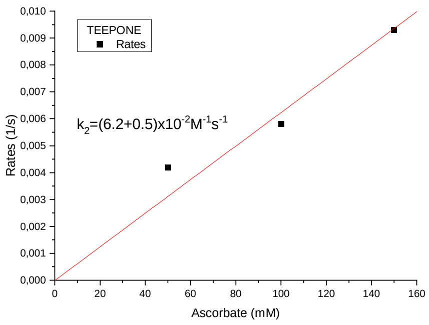
TEEPONE
Rates
k_2=(6.2+0.5)x10^{-2}M^{-1}s^{-1}
Rates (1/s)
Ascorbate (mM)
0,000
0,001
0,002
0,003
0,004
0,005
0,006
0,007
0,008
0,009
0,010
0
20
40
60
80
100
120
140
160

9d

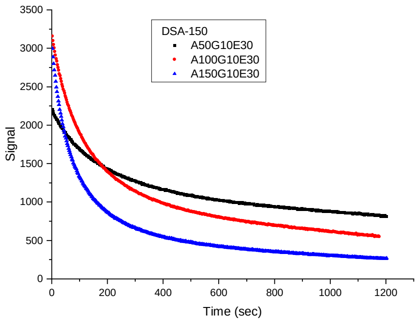
DSA-150
A50G10E30
A100G10E30
A150G10E30
Signal
Time (sec)
0
500
1000
1500
2000
2500
3000
3500
0
200
400
600
800
1000
1200

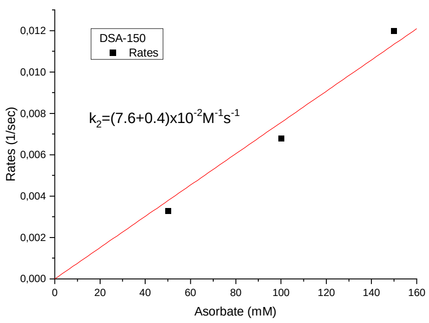
DSA-150
Rates
k_2=(7.6+0.4)x10^{-2}M^{-1}s^{-1}
Rates (1/sec)
Asorbate (mM)
0,000
0,002
0,004
0,006
0,008
0,010
0,012
0
20
40
60
80
100
120
140
160
